# Neurite Outgrowth-Inducing Drimane-Type Sesquiterpenoids Isolated from Cultures of the Polypore *Abundisporus violaceus* MUCL 56355

**DOI:** 10.1021/acs.jnatprod.3c00525

**Published:** 2023-11-01

**Authors:** Winnie Chemutai Sum, Sherif S. Ebada, Marco Kirchenwitz, Lucy Wanga, Cony Decock, Theresia E. B. Stradal, Josphat Clement Matasyoh, Attila Mándi, Tibor Kurtán, Marc Stadler

**Affiliations:** † Department of Microbial Drugs, Helmholtz Centre for Infection Research GmbH (HZI), Inhoffenstraße 7, 38124 Braunschweig, Germany; ‡ Institute of Microbiology, Technische Universität Braunschweig, Spielmannstraße 7, 38106 Braunschweig, Germany; § Department of Pharmacognosy, Faculty of Pharmacy, Ain Shams University, 11566 Cairo, Egypt; ⊥ Department of Cell Biology, Helmholtz Centre for Infection Research, Inhoffenstrasse 7, 38124 Braunschweig, Germany; ∥ Department of Biochemistry, Egerton University, P.O. Box 536, 20115, Njoro, Kenya; ∇ Mycothéque de l’ Universite Catholique de Louvain (BCCM/MUCL), Place Croix du Sud 3, B-1348 Louvain-la-Neuve, Belgium; # Department of Chemistry, Egerton University, P.O. Box 536, 20115, Njoro, Kenya; ¶ Department of Organic Chemistry, University of Debrecen, P.O. Box 400, 4002 Debrecen, Hungary

## Abstract

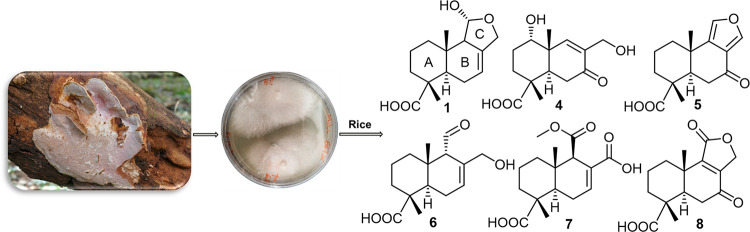

Abundisporin A (**1**), together with seven previously undescribed drimane sesquiterpenes named abundisporins B–H (**2**–**8**), were isolated from a polypore, *Abundisporus violaceus* MUCL 56355 (Polyporaceae), collected in Kenya. Chemical structures of the isolated compounds were elucidated based on exhaustive 1D and 2D NMR spectroscopic measurements and supported by HRESIMS data. The absolute configurations of the isolated compounds were determined by using Mosher’s method for **1**–**4** and TDDFT-ECD calculations for **4** and **5**–**8**. None of the isolated compounds exhibited significant activities in either antimicrobial or cytotoxicity assays. Notably, all of the tested compounds demonstrated neurotrophic effects, with **1** and **6** significantly increasing outgrowth of neurites when treated with 5 ng/mL NGF.

Fungal species from the world’s tropical climates have recently drawn a lot of focus in scientific research due to the potential application of their secondary metabolites in drug research.^1^ Notably, Basidiomycota have proven to be a vast reservoir of biologically active secondary metabolites,^2−4^ especially pleuromutilin derivatives, which are the last class of antibiotics that entered the market for systemic human therapy and have recently received substantial attention.^5,6^

In addition, recent reports reveal the potential application of Basidiomycota metabolites in neurodegenerative disease (NDD) treatment owing to their nerve growth factor (NGF)-potentiating and/or NGF-mimicking effects.^7^ NDDs result in progressive malfunction of the nervous system and affect millions globally, with more than $600 billion incurred in their management.^7^

In our ongoing efforts to find new pharmacologically useful agents, we have recently focused on the African species of this fungal division.^3^ Thus, our current study focuses on the neurotrophic activities of the drimane-type molecules isolated from the polypore *Abundisporus violaceus* MUCL 56355. The research was motivated by previous reports on the neurite-outgrowth-stimulating potential of drimane sesquiterpenoids isolated from *Cyathus africanus* and *C. stercoreus* on NGF-mediated PC-12 cells.^8,9^

The genus *Abundisporus* was first described by Ryvarden in 1998 in the family Polyporaceae.^10^ Currently, it comprises nine species that are distributed mostly in tropical and subtropical regions.^11−13^*Abundisporus* species belong to the numerous taxa of Basidiomycota that have not been studied for the production of secondary metabolites, and hitherto, there are no reports on the chemical composition of the basidiomata or the corresponding mycelial cultures of the genus. We have recently collected and cultured a specimen of *A. violaceus* (Wakef.) Ryvarden, a species that has also been recorded from Zimbabwe, Tanzania, Uganda, Brazil, and China.^14^ In this study, we report spectral data of compounds **1**–**8** in addition to their bioactivity in antimicrobial, cytotoxicity, and neurite outgrowth effects assays.

## Results and Discussion

### Fungal Identification

Morphological examinations revealed that the fungus had an imbricate and laterally fused basidiocarp that upon drying becomes light in weight and hard corky. The hyphal system was dimitic, and the hyphae were generative with clamp connections. The basidiospores were thick-walled, smooth, ellipsoid, and yellowish in color. These characters corroborated with reports of the genus *Abundisporus* in the literature.^11^ Accurate identification was confirmed by DNA sequence analysis of the LSU (large subunit) and the internal transcribed spacer (ITS) regions of the rDNA.

### Structure Elucidation of Drimane-Type Sesquiterpenoid Compounds **1**–**8**

HPLC-DAD/MS screening of the ethyl acetate extract derived from a rice culture of *A. violaceus* MUCL 56355 revealed several protonated molecules, with one at 266 Da as the main product. The extract was subjected to successive chromatographic separation procedures to isolate one known and seven new compounds, trivially named abundisporins A–H (**1**–**8**). The structure of **1** was previously reported by Nozoe et al. in a Japanese patent (1997).^15^ However, no detailed spectral data were reported.1

**Chart 1:**
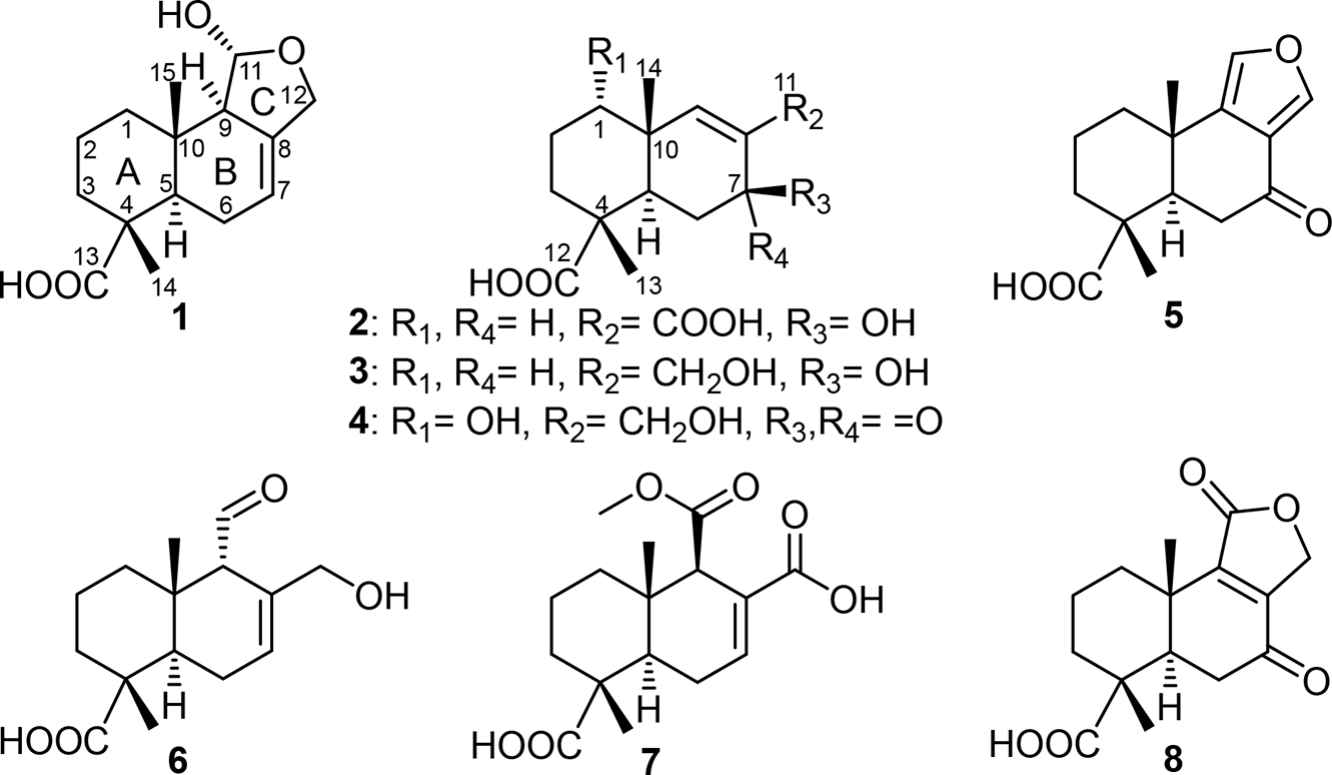


**Figure 1:**
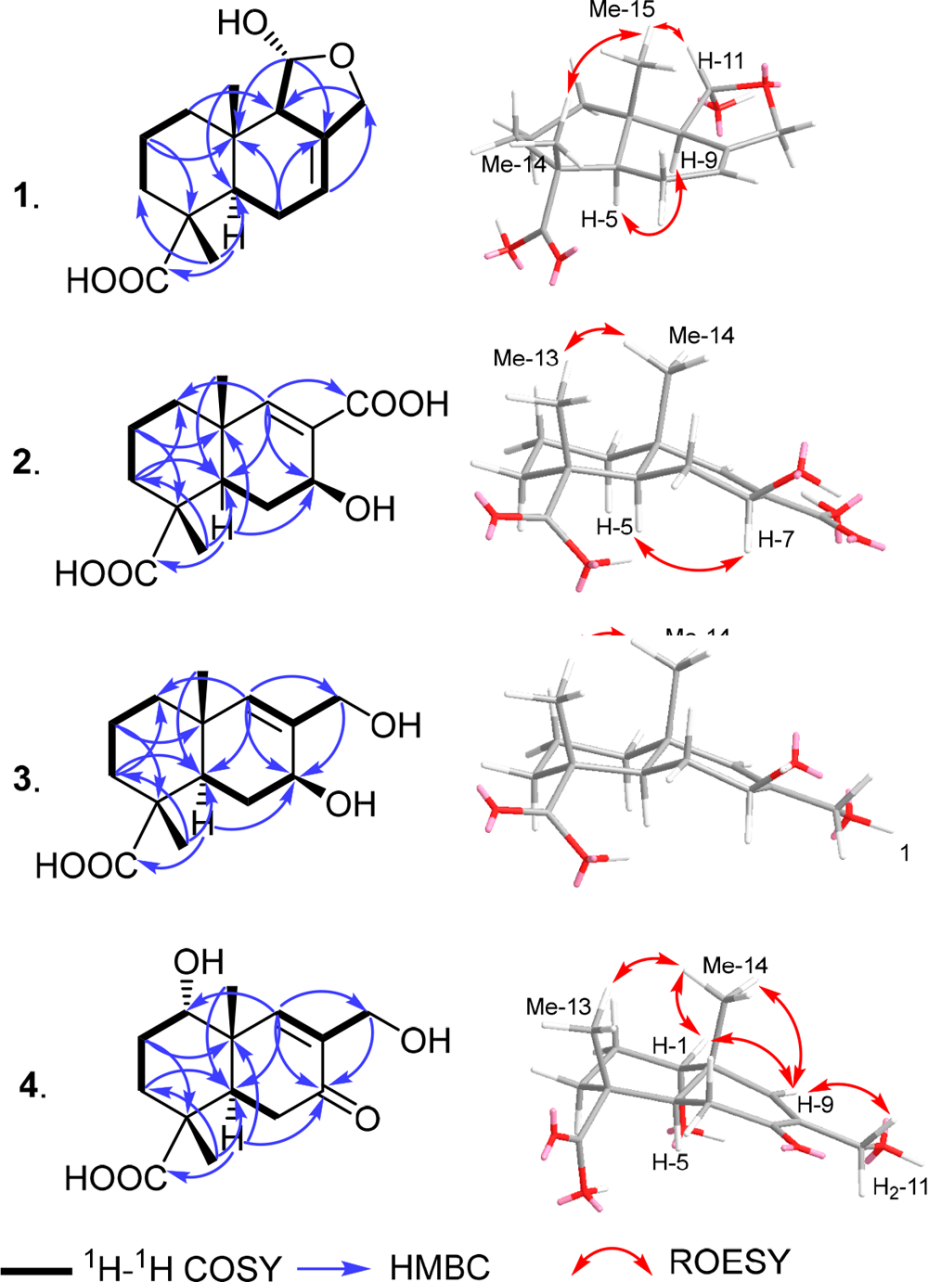
Key ^1^H–^1^H COSY, HMBC, and ROESY correlations of **1**–**4**.

Compound **1** was purified as a brown solid with maximal UV absorption (λ_max_) at 220 nm. The molecular formula of **1** was established to be C_15_H_22_O_4_ based on HRESIMS, which exhibited a sodium adduct at *m*/*z* 289.1406 [M + Na]^+^ (calculated 289.1410) indicating five degrees of unsaturation. The ^13^C NMR and HSQC spectra of **1** (Table 1) revealed 15 carbon resonances including one carboxylic acid carbon at δ_C_ 181.9 (C-13) and two olefinic carbon atoms at δ_C_ 138.1 (C-8) and 117.4 (C-7) accounting for two degrees of unsaturation. The remaining 12 carbon peaks were aliphatic, suggesting that **1** is a tricyclic sesquiterpene derivative. This proposition was further confirmed by ^1^H NMR and ^1^H–^1^H COSY (Figure 1), which revealed two spin systems: one linking three diastereotopic methylene groups at δ_H_ 1.35/δ_H_ 1.85 (H_2_-1), δ_H_ 1.57/δ_H_ 1.66 (H_2_-2), and δ_H_ 1.66/δ_H_ 1.80 (H_2_-3) and the second connecting one methine proton at δ_H_ 2.11 (H-5), one methylene group at δ_H_ 1.86/δ_H_ 2.01 (H_2_-6), and one olefinic proton at δ_H_ 5.48 (H-7) and continuing via long-range correlations to one deshielded methylene group at δ_H_ 4.12/δ_H_ 4.42 (H_2_-12), one methine proton at δ_H_ 2.25 (H-9), and one hemiacetal proton at δ_H_ 5.19 (H-11). A literature search yielded a 1997 patent reporting the planar structure of **1**.^15^

**Table 1:** ^1^H and ^13^C NMR Data of **1**–**4** (500 MHz, Methanol-*d*_4_)

	**1**		**2**		**3**		**4**	
pos.	δ_C_, type	δ_H_ (*J* in Hz)	δ_C_, type	δ_H_ (*J* in Hz)	δ_C_, type	δ_H_ (*J* in Hz)	δ_C_, type	δ_H_ (*J* in Hz)
1	40.1, CH_2_	α 1.35 (dt, 13.4, 3.9)	38.8, CH_2_	α 1.33 (overlapped, m)	39.4, CH_2_	α 1.35 (td, 13.1, 3.8)	75.1, CH	3.49 (dd, 11.2, 4.5)
		β 1.85 (overlapped, m)		β 1.63 (overlapped, m)		β 1.54 (d, 12.8)		
2	18.8, CH_2_	α 1.57 (dp, 10.8, 4.0, 3.6)	18.7, CH_2_	α 1.61 (overlapped, m)	19.3, CH_2_	α 1.60 (overlapped, m)	27.5, CH_2_	α 1.78 (m)
		β 1.66 (overlapped, m)		β 1.79 (overlapped, m)		β 1.75 (overlapped, m)		β 1.86 (m)
3	38.6, CH_2_	α 1.66 (overlapped, m)	37.9, CH_2_	α 1.64 (overlapped, m)	37.9, CH_2_	α 1.62 (overlapped, m),	36.4, CH_2_	α 1.72 (dt, 12.9, 3.3)
		β 1.80 (dd, 13.5, 3.5)		β 1.78 (overlapped, m)		β 1.94 (td, 14.1, 13.4, 4.6)		β 1.95 (td, 13.2, 4.1)
4	46.9, C		47.5, C		47.8, C		46.8, C	
5	45.9, CH	2.11 (dd, 11.6, 5.1)	45.0, CH	2.00 (dd, 13.1, 1.8)	42.0, CH	2.27 (dd, 13.2, 2.0)	45.6, CH	2.16 (dd, 17.0, 3.1)
6	26.0, CH_2_	α 1.86 (m)	31.6, CH_2_	α 1.63 (overlapped, m)	32.2, CH_2_	α 1.50 (dt, 14.0, 1.8)	37.7, CH_2_	α 2.51 (dt, 14.2, 3.1)
		β 2.01 (m)		β 1.79 (overlapped, m)		β 1.83 (td, 13.4, 4.7)		β 2.62 (dd, 17.0, 14.2)
7	117.4, CH	5.48 (tt, 2.9, 1.2)	68.8, CH	4.57 (ddd, 9.5, 7.4, 1.2)	65.7, CH	4.18 (dd, 4.5, 1.4)	201.3, CO	
8	138.1, C		131.6, C		135.8, C		136.2, C	
9	62.2, CH	2.25 (m)	153.4, CH	6.60 (d, 1.2)	140.7, CH	5.50 (d, 1.2)	155.1, CH	7.27 (t, 1.5)
10	34.0, C		36.9, C		36.3, C		43.0, C	
11	100.0, CH	5.19 (d, 5.0)	171.1, CO		64.7, CH_2_	α 3.98 (d, 12.8)	59.9, CH_2_	4.16 (dd, 4.0, 1.5)
						β 4.11 (d, 12.8)		
12	69.1, CH_2_	α 4.12 (dt, 11.6, 1.7)	181.7, CO		182.0, CO		180.7, CO	
		β 4.42 (dddd, 11.5, 4.9, 3.0, 1.9)						
13	181.9, CO		16.6, CH_3_	1.22 (s)	16.7, CH_3_	1.18 (s)	16.4, CH_3_	1.26 (s)
14	17.2, CH_3_	1.24 (s)	20.5, CH_3_	1.14 (s)	20.3, CH_3_	0.98 (s)	13.6, CH_3_	1.12 (s)
15	14.7, CH_3_	0.88 (s)						

The HMBC spectrum revealed correlations from the hemiacetal proton assigned as H-11 to four carbons at δ_C_ 138.1 (C-8), 69.1 (C-12), 62.2 (C-9), and 34.0 (C-10) and also from the deshielded methylene group ascribed as H_2_-12 to δ_C_ 138.1 (C-8), 117.4 (C-7), 100.0 (C-11), and 62.2 (C-9), confirming the presence of a tetrahydrofuran ring as depicted. In addition, the relative configuration of **1** was determined based on the ROESY spectrum (Figure 1), which showed clear NOE correlations from a shielded singlet methyl group at δ_H_ 0.88 (Me-15) to H-11 and to another singlet methyl group at δ_H_ 1.24 (Me-14), indicating that they are all on the same face of the molecule, while H-5 revealed a NOE correlation to H-9 at δ_H_ 2.25 (m), indicating that they are on the opposite face.

To determine the absolute configuration of **1**, we prepared (*R*)- and (*S*)-MTPA esters at the C-11 hydroxy group using Mosher’s method.^16,17^ The obtained esters exhibited positive Δδ^*SR*^ values at H_2_-1, H_2_-2, H-9, Me-14, and Me-15 and negative values at H-7 and H_2_-12, allowing us to assign the configuration at C-11 as (*S*) and thus the absolute configuration of **1** as (4*R*,5*R*,9*R*,10*S*,11*S*). Accordingly, we described in this study detailed 1D (^1^H/^13^C) and 2D NMR spectral data of **1** (Table 1), to which we give a trivial name abundisporin A.

Compound **2** was obtained as a brown solid, and its molecular formula established as C_14_H_20_O_5_ based on HRESIMS, which displayed a protonated molecule at *m*/*z* 269.1379 [M + H]^+^ (calculated 269.1384), indicating five degrees of unsaturation as in **1**. However, the ^1^H and ^13^C NMR data of **2** (Table 1) showed some distinguishing chemical features: an additional carbonyl carbon atom at δ_C_ 171.1 (C-11) replaced the hemiacetal and deshielded methylene protons in **1**. This suggests the absence of the tetrahydrofuran ring in **2**, and hence it has a bicyclic sesquiterpene skeleton. The ^1^H NMR, ^1^H–^1^H COSY, and HSQC spectral data of **2** (Table 1) revealed two main spin systems: the first extends over three adjacent diastereotopic methylene groups (H_2_-1 to H_2_-3) as in **1**, while the second begins at a methine proton at δ_H_ 2.00 (H-5; δ_C_ 45.0), a diastereotopic methylene group at δ_H_ 1.63/δ_H_ 1.79 (H_2_-6; δ_C_ 31.6), and an oxygenated methine proton at δ_H_ 4.57 (H-7; δ_C_ 68.8) and extends via a long-range COSY correlation to an olefinic proton at δ_H_ 6.60 (H-9; δ_C_ 153.4), indicating the presence of a hydroxy group at C-7 and an α,β-unsaturated ketocarbonyl moiety in **2**, respectively. The HMBC spectrum of **2** (Figure 1) revealed key correlations from H-9 and H-7 to a quaternary carbon at δ_C_ 131.6 (C-8) and the carbonyl carbon at δ_C_ 171.1 (C-11), indicating the presence of a carboxylic acid moiety at C-8. A literature search disclosed that compound **2** is a 12-COOH derivative of the plant metabolites epipolypiperic acid^18^ and isopolygonal acid.^19^

The relative configuration of **2** was determined based on the ROESY spectrum (Figure 1), which revealed NOE correlations between two methyl groups (Me-14 and Me-15), indicating that they are both on the same face of the structure, whereas H-5 and H-7 revealed key common NOE correlations indicating that they are on the opposite face.

**Figure 2:**
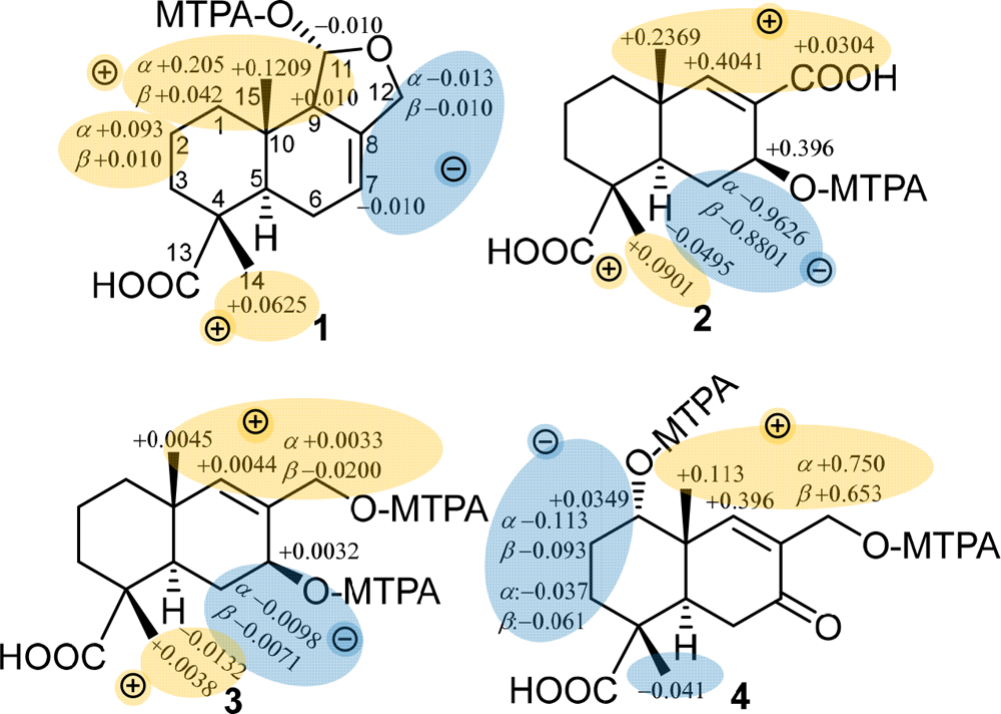
Δδ^*SR*^ values of (*S*)/(*R*)-MTPA esters obtained from abundisporin A (**1**) diagnostic for (11*R*), abundisporins B (**2**) and C (**3**) diagnostic for (7*S*), and abundisporin D (**4**) diagnostic for (1*S*).

The absolute configuration of **2** was determined by the analysis of its corresponding C-7 (*S*)- and (*R*)-MTPA esters following Mosher’s method.^16,17^ The obtained esters (Figure 2) exhibited positive Δδ^*SR*^ values at H-9, H-11, Me-13, and Me-14 and negative Δδ^*SR*^ values at H-5 and H_2_-6. Thus, the configuration at C-7 was assigned as (*S*), and the absolute configuration of **2** was assigned as (4*R*,5*R*,7*S*,10*R*). Compound **2** was identified as a new sesquiterpene derivative, and it was named abundisporin B.

Compound **3** was isolated as a brown oil, and its molecular formula was determined to be C_14_H_22_O_4_ based on HRESIMS, which displayed a sodium adduct at *m*/*z* 277.1405 [M + Na]^+^ (calculated 277.1410), indicating four degrees of unsaturation. The ^13^C NMR spectrum of **3** (Table 1) revealed one carboxylic acid carbon atom at δ_C_ 182.0 (C-12) and two olefinic carbon atoms at δ_C_ 140.7 (C-9) and 135.8 (C-8), accounting for two degrees of unsaturation and suggesting that **3** features a bicyclic sesquiterpene skeleton similar to **2**. The main structural difference was the presence of an oxygenated methylene group at δ_H_ 4.11/δ_H_ 3.98 with a large geminal coupling constant of 12.8 Hz. The position of this methylene group at C-8 was confirmed using HMBC (Figure 1), which showed clear correlations from the methylene protons to three carbon atoms: an oxygenated methine carbon at δ_C_ 65.7 (C-7) and two olefinic carbons at δ_C_ 140.7 (C-8) and 135.8 (C-9). The ROESY spectrum of **3** (Figure 1) disclosed key NOE correlations from H-7 to both H-5 and H_2_-11, indicating that they are all on the same face of the molecule, whereas the two methyl groups Me-14 and Me-15 exhibited an NOE correlation, suggesting that they are on the opposite face.

The absolute configuration of **3** was determined using Mosher’s method.^16,17^ The obtained Δδ^*SR*^ values for **3** (Figure 2) revealed a pattern similar to those calculated for **2**, consistent with an identical absolute configuration for **3** of (4*R*,5*R*,7*S*,10*R*). Compound **3** was identified as a new sesquiterpene derivative that was named abundisporin C.

Compound **4** was obtained as a brown oil, and HRESIMS established its molecular formula to be C_14_H_20_O_5_, the same as **2**, based on a protonated molecule at *m*/*z* 269.1381 [M + H]^+^ (calculated 269.1384). The ^13^C NMR and HSQC spectra of **4** (Table 1) revealed a deshielded ketocarbonyl carbon at δ_C_ 201.3 (C-7) together with two olefinic carbon atoms at δ_C_ 155.1 (C-9) and 136.2 (C-8), suggesting their existence as an α,β-unsaturated ketone moiety. Moreover, the ^1^H NMR data of **4** (Table 1) also revealed the presence of an oxygenated aliphatic methine proton at δ_H_ 3.49 (H-1), which was illustrated by ^1^H–^1^H COSY (Figure 1) to form a spin system with two diastereotopic methylene groups at δ_H_ 1.78/δ_H_ 1.86 (H_2_-2) and δ_H_ 1.72/δ_H_ 1.95 (H_2_-3). This suggested that the additional hydroxy group is attached to C-1 in ring A. In addition, ^1^H NMR and HSQC spectra of **4** also revealed the presence of a deshielded methylene group at δ_H_ 4.16 (H_2_-11; δ_C_ 59.9). Further confirmation of the positions of ketocarbonyl and hydroxy groups was provided by HMBC (Figure 1), which revealed correlations from two methylene groups at δ_H_ 4.16 (H_2_-11) and δ_H_ 2.51/δ_H_ 2.62 (H_2_-6) and two methine protons at δ_H_ 2.16 (H-5) and δ_H_ 7.27 (H-9) to the ketocarbonyl group, thus confirming its position at C-7. The hydroxy group was positioned at C-1 based on key HMBC correlations from H-9, H_2_-2, H_2_-3, and Me-14 to an oxygenated aliphatic carbon at δ_C_ 75.1 (C-1).

The ROESY spectrum of **4** (Figure 1) exhibited key NOE correlations between H-1, Me-13, and Me-14, indicating that they are all on the same face of the molecule and opposite to H-5.

**Figure 3:**
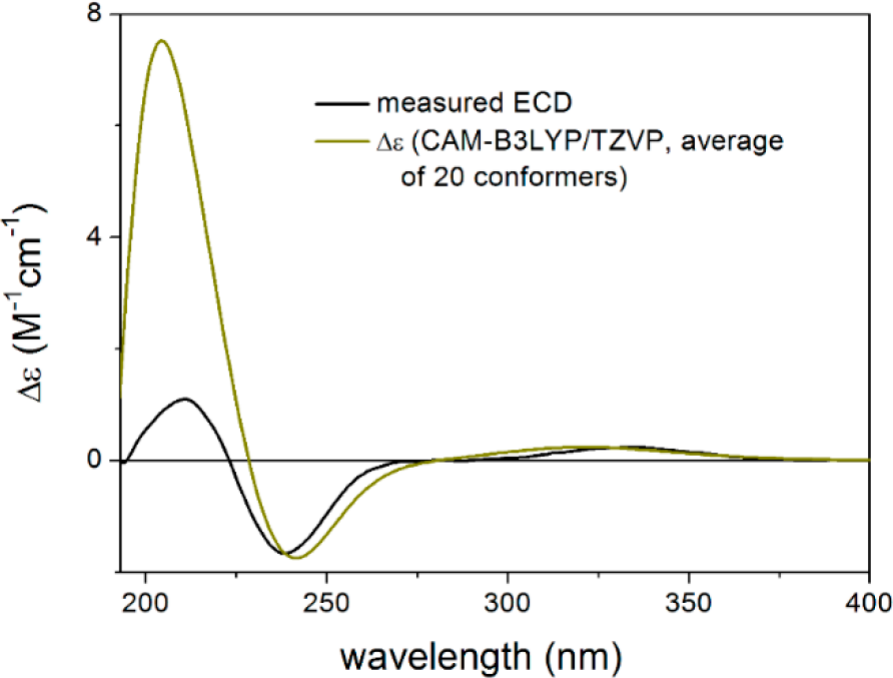
Experimental ECD spectrum of **4** (black) compared with the CAM-B3LYP/TZVP PCM/MeOH ECD spectrum of (1*S*,4*R*,5*R*,10*R*)-**4** (dark yellow). Level of DFT optimization: ωB97X/TZVP PCM/MeOH.

The absolute configuration of **4** was determined by the analysis of its corresponding C-1 (*S*)- and (*R*)-MTPA esters following Mosher’s method.^16,17^ The obtained esters (Figure 2) revealed positive Δδ^*SR*^ values at H_2_-11 and Me-14 and negative Δδ^*SR*^ values at H_2_-1 and H_2_-2. Thus, the configuration at C-1 was assigned as (*S*) and the absolute configuration of **4** as (1*S*,4*R*,5*R*,10*R*). In order to verify the absolute configuration independently from Mosher’s NMR analysis, TDDFT-ECD calculations were performed on the (1*S*,4*R*,5*R*,10*R*)**-4**.^20,21^ The initial 76 Merck molecular force field (MMFF) conformers were reoptimized at the ωB97X/TZVP^22^ PCM/MeOH level yielding 20 low-energy conformers above 1% Boltzmann population (Figure S35). Boltzmann-averaged ECD spectra computed at four different levels of theory reproduced all transitions of the experimental ECD spectrum, suggesting an absolute configuration for **4** of (1*S*,4*R*,5*R*,10*R*) (Figure 3). It is interesting to note that depending on how the 8-OH coordinates to the C-7 carbonyl oxygen with an intramolecular hydrogen bond, there are two major groups of conformers with considerably different computed ECD spectra (group A: conformers A, C, D, F, I, L, O, and P; group B: conformers B, E, G, H, J, K, M, Q, and R). This manifested in oppositely signed ECD transitions or shoulders at about 325 and 240 nm, while the positive Cotton effect (CE) at around 220 nm seems to be unchanged, and it can serve for the solid assignment of the absolute configuration (Figures S35 and S36). Accordingly, compound **4** was confirmed to be a new sesquiterpene derivative that was named abundisporin D.

**Figure 4:**
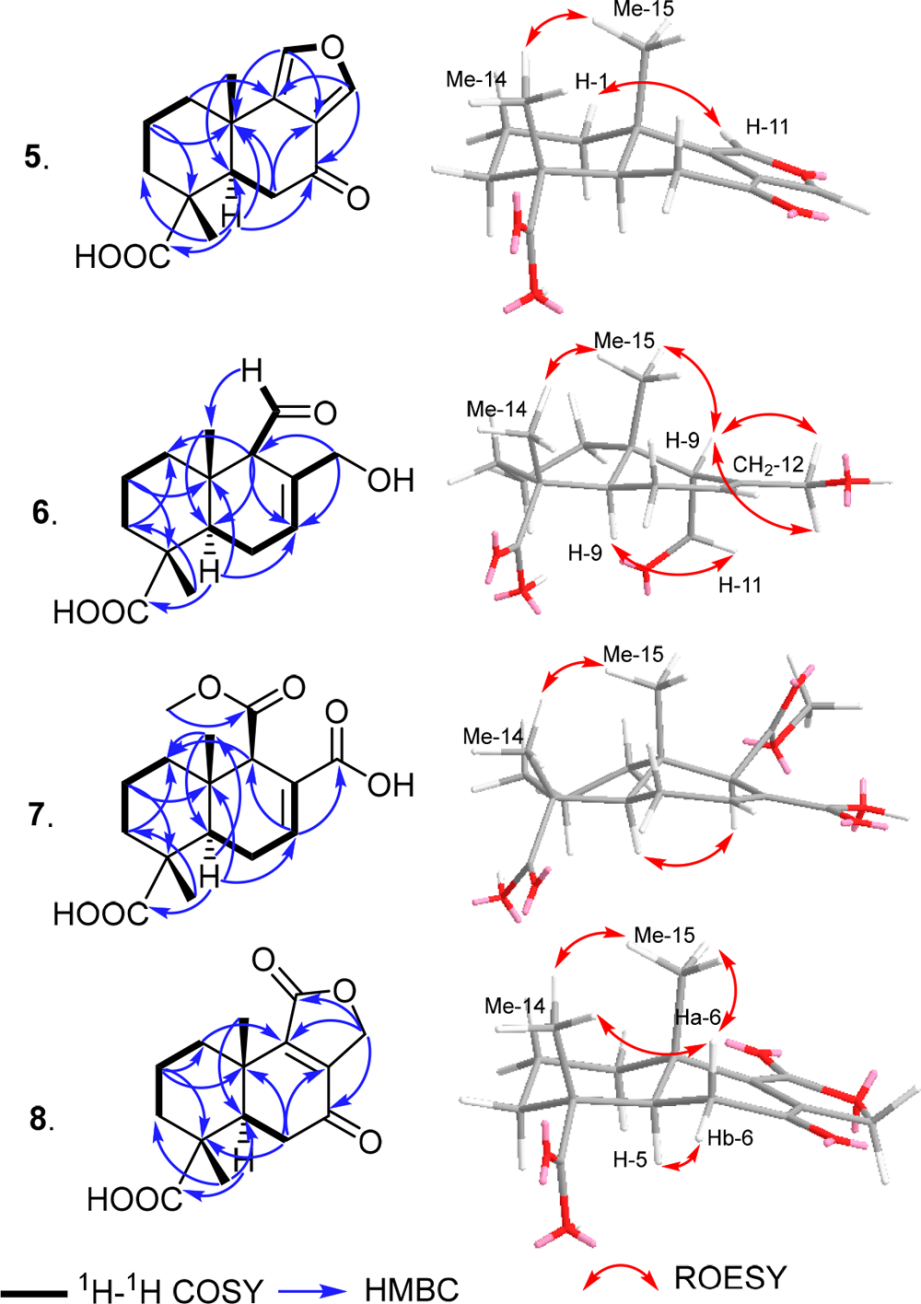
Key ^1^H–^1^H COSY, HMBC, and ROESY correlations of **5**–**8**.

Compound **5** was isolated as a brown solid with the molecular formula C_15_H_18_O_4_ based on the HRESIMS spectrum, showing a protonated molecule at *m*/*z* 263.1277 [M + H]^+^ (calculated 263.1278) indicating seven degrees of unsaturation. The ^1^H NMR, ^1^H–^1^H COSY, and HSQC spectra of **5** (Table 2) revealed two doublet deshielded proton resonances at δ_H_ 7.43 and 8.03 with a common coupling constant of 1.5 Hz directly correlated to two deshielded olefinic carbon atoms at δ_C_ 138.0 and 145.9, respectively. These results suggested a probable furan moiety as ring C, supported by different maximal UV absorptions (λ_max_) at 228 and 278 nm, consistent with a different chromophore. The ^13^C NMR data of **5** (Table 2), like those of **4**, revealed four quaternary carbon atoms including a ketocarbonyl at δ_C_ 197.3 (C-7), a carboxycarbonyl at δ_C_ 181.1 (C-13), and two aliphatic carbon atoms at δ_C_ 47.2 (C-4) and 34.5 (C-10). It also revealed two quaternary olefinic carbon atoms at δ_C_ 139.5 (C-9) and 124.1 (C-8). Further confirmation of the structure of **5** was provided by its HMBC spectrum (Figure 4), which exhibited clear correlations from the two doublet deshielded protons at δ_H_ 8.03 (H-12) to C-9 and C-11 and δ_H_ 7.43 (H-11) to C-9 and C-12 together with common correlations to the ketocarbonyl carbon atom at C-7, confirming the presence of ring C as a furan moiety resembling the previously reported semisynthetic fungal metabolite 7-ketoeuryfuran.^23,24^

**Table 2:** ^1^H and ^13^C NMR Data of **5**–**8** (500 MHz, Methanol-*d*_4_)

	**5**		**6**		**7**		**8**	
pos.	δ_C_, type	δ_H_ (*J* in Hz)	δ_C_, type	δ_H_ (*J* in Hz)	δ_C_, type	δ_H_ (*J* in Hz)	δ_C_, type	δ_H_ (*J* in Hz)
1	38.6, CH_2_	1.66 (td, 12.8, 3.9, 2H)	37.9, CH_2_	α 1.40 (td, 12.9, 4.1)	40.8, CH_2_	α 1.44 (td, 13.1, 4.3)	34.0, CH_2_	α 1.47 (td, 13.1, 4.0)
				β 1.57 (overlapped, m)		β 1.87 (dt, 13.4, 2.3)		β 2.62 (m)
2	18.9, CH_2_	α 1.71 (overlapped, m)	18.9, CH_2_	α 1.58 (overlapped, m)	18.8, CH_2_	1.60 (m)	18.4, CH_2_	α 1.69 (overlapped, m)
		β 1.83 (overlapped, m)		β 1.75 (overlapped, m)				β 1.84 (overlapped, m)
3	38.1, CH_2_	α 1.75 (overlapped, m)	38.8, CH_2_	α 1.69 (overlapped, m)	38.3, CH_2_	α 1.67 (m)	37.8, CH_2_	α 1.71 (overlapped, m)
		β 1.83 (overlapped, m)		β 1.78 (overlapped, m)		β 1.79 (td, 12.8, 4.7)		β 1.85 (ddt, 8.6, 6.8, 2.2)
4	47.2, C		47.0, C		47.1, C		47.4, C	
5	47.0, CH	2.57 (dd, 13.7, 2.7)	41.1, CH	2.58 (dd, 11.3, 5.7)	44.7, CH	2.09 (dd, 11.8, 4.0)	47.5, CH	2.73 (dd, 11.0, 3.2)
6	39.8, CH_2_	α 2.21 (dt, 17.4, 2.7)	26.4, CH_2_	α 2.05 (m)	26.5, CH_2_	α 2.03 (m)	39.0, CH_2_	α 2.27 (dd, 14.4, 11.0)
		β 2.70 (dd, 17.5, 13.8)		β 2.10 (m)		β 2.24 (dddd, 18.5, 11.9, 4.1, 2.2)		β 2.74 (d, 14.4, 3.2)
7	197.3, CO		128.0, CH	5.98 (ddt, 4.3, 2.8, 1.3)	140.6, CH	6.99 (dt, 5.9, 2.2)	196.8, CO	
8	124.1, C		132.7, C		130.5, C		151.3, C	
9	139.5, C		65.0, CH	2.52 (dd, 4.8, 1.7)	59.1, CH	3.23 (dt, 4.3, 2.2)	152.1, C	
10	34.5, C		36.6, C		36.5, C		37.3, C	
11	138.0, CH	7.43 (d, 1.5)	203.8, CO	9.67 (d, 4.8)	174.7, CO		172.9, CO	
12	145.9, CH	8.03 (d, 1.5)	65.6, CH_2_	α 3.78 (dd, 13.1, 1.7)	170.7, CO		68.8, CH_2_	α 4.85 (d, 17.5)
				β 3.83 (dd, 13.0, 1.4)				β 4.89 (d, 17.5)
13	181.1, CO		181.8, CO		181.7, CO		181.2, CO	
14	16.8, CH_3_	1.31 (s)	17.6, CH_3_	1.26 (s)	17.6, CH_3_	1.28 (s)	16.6, CH_3_	1.31 (s)
15	23.6, CH_3_	1.32 (d, 0.7)	21.9, CH_3_	1.01 (d, 0.7)	16.2, CH_3_	0.93 (s)	18.4, CH_3_	1.34 (s)
16					51.9, CH_3_	3.64 (s)		

The relative configuration of **5** was determined using the ROESY spectrum (Figure 4), which revealed a key NOE correlation between two methyl groups at δ_H_ 1.31 (δ_C_ 16.8) and δ_H_ 1.32 (δ_C_ 23.6), assigned as Me-14 and Me-15, respectively, indicating that they are both on the same face of the molecule, with H-5 (δ_H_ 2.57, dd, *J* = 13.7, 2.7 Hz; δ_C_ 47.0) on the opposite face. Based on the common biosynthetic origin of compounds **1**–**5** and key NOE correlations similar to those previously observed for abundisporin A (**1**), the absolute configuration of **5** was suggested to be (4*R*,5*R*,10*S*).

**Figure 5:**
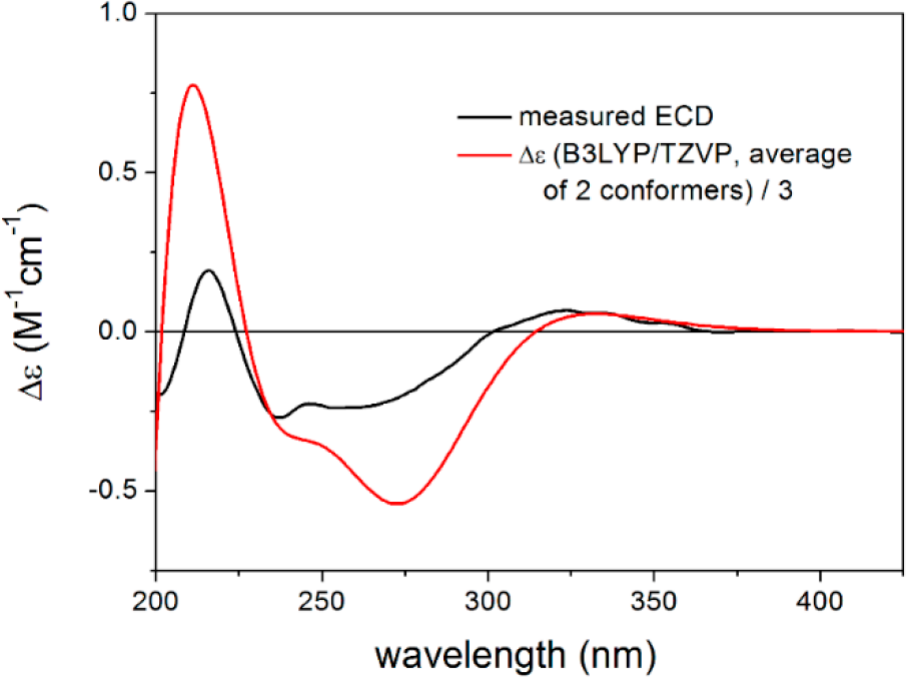
Experimental ECD spectrum of **5** (black) compared with the B3LYP/TZVP PCM/MeOH ECD spectrum of (4*R*,5*R*,10*S*)-**5** (red). Level of DFT optimization: ωB97X/TZVP PCM/MeOH.

In order to verify the absolute configuration assumed by the biosynthetic origin, the TDDFT-ECD protocol was applied on (4*R*,5*R*,10*S*)-**5**. Boltzmann-averaged ECD spectra of the two low-energy ωB97X/TZVP conformers obtained from the reoptimization of the seven initial MMFF conformers reproduced well the sign of all the transitions of the experimental ECD spectrum at all the applied combinations of theoretical levels (Figure 5). Thus, the ECD calculations confirmed the (4*R*,5*R*,10*S*) absolute configuration. According to the obtained results, compound **5** was identified as a new sesquiterpene derivative comprising a condensed furan moiety, and it was trivially named as abundisporin E.

Compound **6** was obtained as a brown solid, and its HRESIMS revealed a sodium adduct at *m*/*z* 289.1406 [M + Na]^+^ consistent with a molecular formula of C_15_H_22_O_4_ (calculated 289.1406) and indicating five degrees of unsaturation, similar to abundisporin A (**1**). Unlike abundisporin A, the ^1^H and ^13^C NMR and HSQC spectra of **6** (Table 2) revealed a deshielded proton at δ_H_ 9.67 (H-11) that was directly correlated to a ketocarbonyl carbon at δ_C_ 203.8, indicating an aldehyde moiety. The HMBC spectrum of **6** (Figure 4) exhibited key correlations from the aldehyde proton to four carbon peaks at δ_C_ 132.7 (C-8), 65.0 (C-9), 36.6 (C-10) and δ_C_ 21.9 (C-15), hence confirming the position of the aldehyde group at C-9.

**Figure 6:**
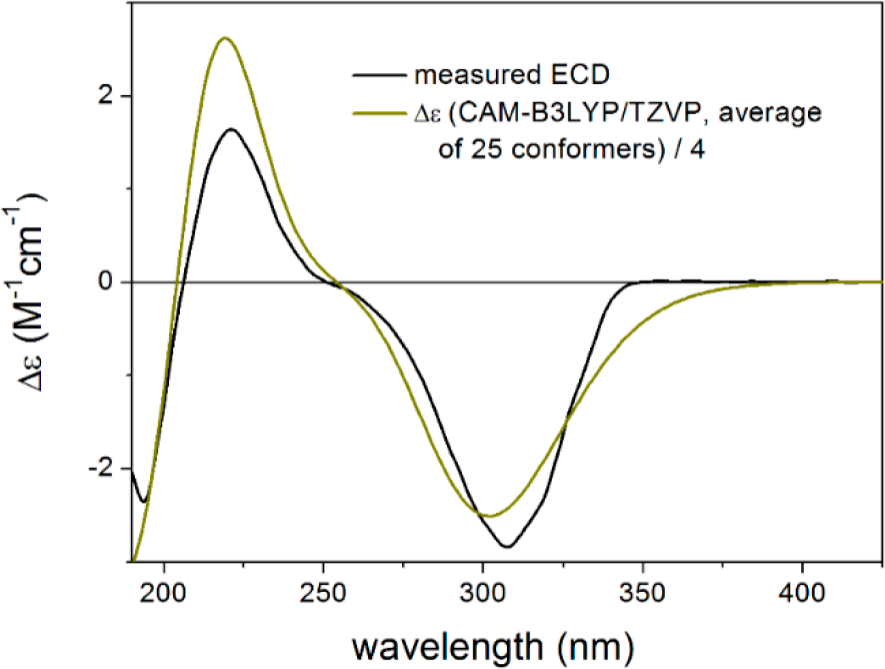
Experimental ECD spectrum of **6** (black) compared with the CAM-B3LYP/TZVP PCM/MeOH ECD spectrum of (4*R*,5*R*,9*S*,10*S*)-**6** (dark yellow). Level of DFT optimization: ωB97X/TZVP PCM/MeOH.

The relative configuration of **6** was defined based on its ROESY spectrum (Figure 4), which included NOE correlations between Me-15 (δ_H_ 1.01) and Me-14 (δ_H_ 1.26), indicating that they are both on the same face of the molecule, and between H-5 (δ_H_ 2.58) and H-11, indicating that they are on the opposite face. Given their structural similarity to and presumed shared biogenesis with the drimane-type sesquiterpenes, compounds **1**–**6** were expected to possess the same (4*R*,5*R*,10*S*) configuration, and the configuration of **6** at C-9 was subsequently deduced to be (9*S*). TDDFT-ECD calculations were performed to confirm this independently. MMFF conformational analysis of (4*R*,5*R*,9*S*,10*S*)-**6** yielded 61 initial conformers, the ωB97X/TZVP PCM/MeOH reoptimization of which resulted in 25 low-energy conformers over 1% Boltzmann population. Boltzmann-averaged ECD spectra obtained at various levels of theory gave nice agreement with the experimental ECD spectrum, verifying the (4*R*,5*R*,9*S*,10*S*) absolute configuration (Figure 6). Therefore, compound **6** was identified as a new sesquiterpene derivative, and it was given the trivial name abundisporin F.

Compound **7** was purified as a brown solid, and its molecular formula was determined to be C_16_H_22_O_6_ based on HRESIMS, which revealed a protonated molecule at *m*/*z* 311.1487 [M + H]^+^ (calculated 311.1489), indicating six degrees of unsaturation. The ^1^H and ^13^C NMR and HSQC spectral data of **7** (Table 2) showed the presence of a deshielded proton at δ_H_ 6.99 (H-7), which is directly correlated to a deshielded olefinic carbon at δ_C_ 140.6 (C-7) together with the presence of two carboxycarbonyl carbons at δ_C_ 174.7 (C-11) and 170.7 (C-12) in addition to the common carboxylic acid carbon at δ_C_ 181.7 (C-13) in compounds **1**–**6**. The ^1^H NMR spectrum of **7** (Table 2) also disclosed the presence of a methoxy group at δ_H_ 3.64 (Me-16), which was directly correlated to an oxygenated primary carbon at δ_C_ 51.9 ppm. The HMBC spectrum (Figure 4) revealed key correlations from both the methoxy group and a methine proton at δ_H_ 3.23 (H-9; δ_C_ 59.1) to a carboxycarbonyl carbon at δ_C_ 174.7 (C-11), indicating its existence as a methyl ester moiety at C-9. In addition, HMBC correlations from the deshielded proton at δ_H_ 6.99 (H-7) and two diastereotopic methylene protons at δ_H_ 2.03/δ_H_ 2.24 (H_2_-6) to a carboxycarbonyl carbon (C-12) confirm its presence as a free carboxylic acid functionality.

The relative configuration of **7** was determined based on the NOE correlations as revealed by its ROESY spectrum (Figure 4). Correlations between Me-14 (δ_H_ 1.28) and Me-15 (δ_H_ 0.93) indicated that they are on the same face, whereas correlations between H-5 (δ_H_ 2.09) and H-9 (δ_H_ 3.23) indicate that they are on the opposite face.

**Figure 7:**
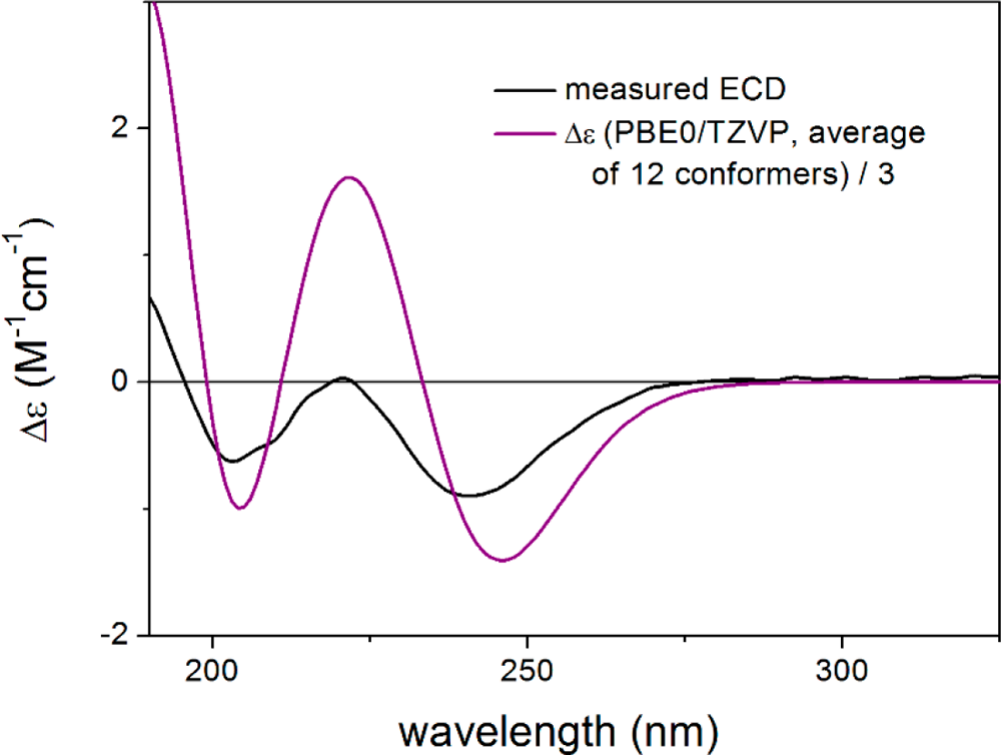
Experimental ECD spectrum of **7** (black) compared with the PBE0/TZVP PCM/MeOH ECD spectrum of (4*R*,5*R*,9*R*,10*S*)-**7** (purple). Level of DFT optimization: ωB97X/TZVP PCM/MeOH.

The absolute configuration of **7** was determined to be (4*R*,5*R*,9*R*,10*S*) on the basis of its structural similarity to abundisporin F (**6**) and by comparing its NOE correlations with those obtained from **1**. This absolute configuration was also independently confirmed by TDDFT-ECD calculations (Figure 7). The ωB97X/TZVP PCM/MeOH reoptimization of the initial 18 MMFF conformers of (4*R*,5*R*,9*R*,10*S*)-**7** resulted in 12 low-energy conformers, the Boltzmann averaged ECD spectra of which gave acceptable agreement with the experimental spectrum overestimating the small positive transition at ∼225 nm. This derived probably from the lower predicted populations of conformers B and E, in which the ester carbonyl had a different orientation, and they had no intense computed ECD transitions at about 225 nm. Thus, the absolute configuration of **7** was determined as (4*R*,5*R*,9*R*,10*S*). Based on the obtained results, compound **7** was concluded to be a new sesquiterpene derivative featuring a methyl ester moiety and was named abundisporin G.

Compound **8** was purified as a brown solid, and its molecular formula was established to be C_15_H_18_O_5_ based on HRESIMS, displaying a protonated molecule and a sodium adduct at *m*/*z* 279.1229 [M + H]^+^ (calculated 279.1227) and at *m*/*z* 301.1047 [M + Na]^+^ (calculated 301.1046), respectively, indicating seven degrees of unsaturation. The UV spectrum of **8** revealed a maximal absorption (λ_max_) at 248 nm, suggesting a possible lactone moiety in its structure.^25^ The ^13^C NMR data of **8** (Table 2) revealed the presence of a ketocarbonyl carbon (δ_C_ 196.8, C-7) along with a second carbonyl carbon at δ_C_ 172.9 (C-11), two deshielded olefinic quaternary carbons at δ_C_ 152.1 (C-9) and δ_C_ 151.3 (C-8) together with the common carboxylic acid carbon at δ_C_ 181.2 (C-13). The ^1^H NMR, HMBC, and HSQC spectra of **8** (Figure 4) revealed the presence of a deshielded diastereotopic methylene group at δ_H_ 4.85/δ_H_ 4.89 (H_2_-12) with the characteristic large geminal coupling constant (*J* value) of 17.5 Hz and directly correlated to a carbon peak at δ_C_ 68.8 ppm. The HMBC spectrum of **8** (Figure 4) revealed key correlations from H_2_-12 to C-8, C-9, and C-11, indicating that ring C is a lactone in compound **8**. Other HMBC correlations from two diastereotopic methylene groups at δ_H_ 2.27/δ_H_ 2.74 (H_2_-6) and δ_H_ 4.85/δ_H_ 4.89 (H_2_-12) to the ketocarbonyl carbon atom at δ_C_ 196.8 indicated its location at C-7.

**Figure 8:**
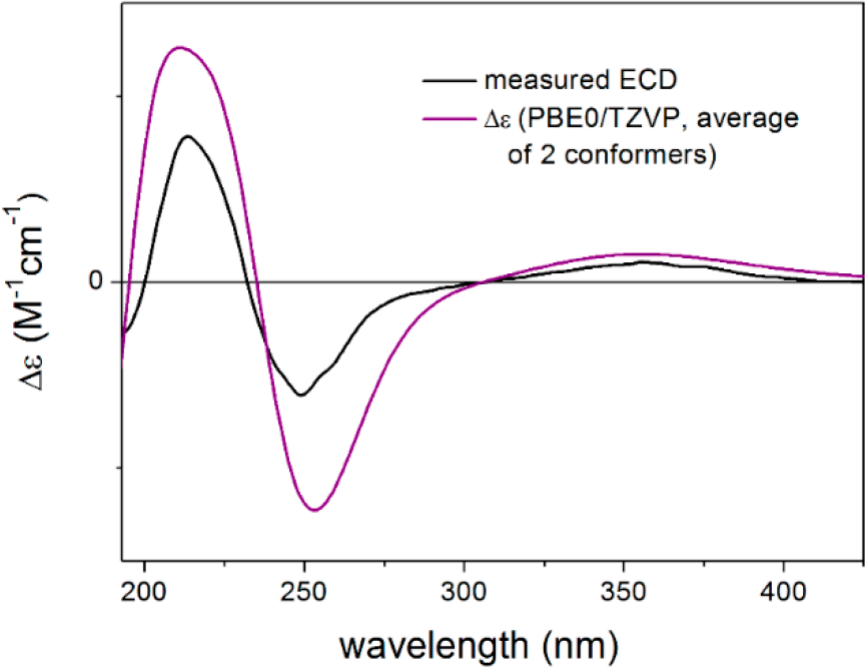
Experimental ECD spectrum of **8** (black) compared with the PBE0/TZVP PCM/MeOH ECD spectrum of (4*R*,5*R*,10*S*)-**8** (purple). Level of DFT optimization: ωB97X/TZVP PCM/MeOH.

The relative configuration of **8** was determined based on the ROESY spectrum (Figure 4), which revealed similar NOE correlations to other abunisporins, including between Me-14 (δ_H_ 1.31), Me-15 (δ_H_ 1.34), and Hβ-6 (δ_H_ 2.74), indicating that they are all on the same face of the molecule, and between H-5 (δ_H_ 2.73) and Hα-6 (δ_H_ 2.27), suggesting they are on the opposite face. Based on the structural similarity of **8** to abundisporin E (**5**) (both possess the same number of chiral centers at comparable carbons in their drimane skeleton) in combination with their close optical rotation values and by comparing their NOE correlations with those obtained from abundisporin A (**1**), the absolute configuration of **8** was determined to be (4*R*,5*R*,10*S*). The absolute configuration of **8** was also confirmed independently by TDDFT-ECD calculations of the (4*R*,5*R*,10*S*) stereoisomer. The ωB97X/TZVP PCM/MeOH reoptimization of the initial 10 MMFF conformers yielded two low-energy conformers over 1% Boltzmann population (Figure S69), the averaged ECD spectra of which reproduced all the experimental ECD transitions of **8** (Figure 8) at four levels of theory.

Furthermore, the two major conformers with a sum Boltzmann population of 99.3% exhibited very similar computed ECD spectra, allowing solid assignment of the absolute configuration of **8** as (4*R*,5*R*,10*S*). A literature search of **8** revealed that it is a 7-keto derivative of the plant metabolite caloterpene,^26^ and it was named abundisporin H.

### Biological Activity of Compounds **1**–**8**

**Figure 9:**
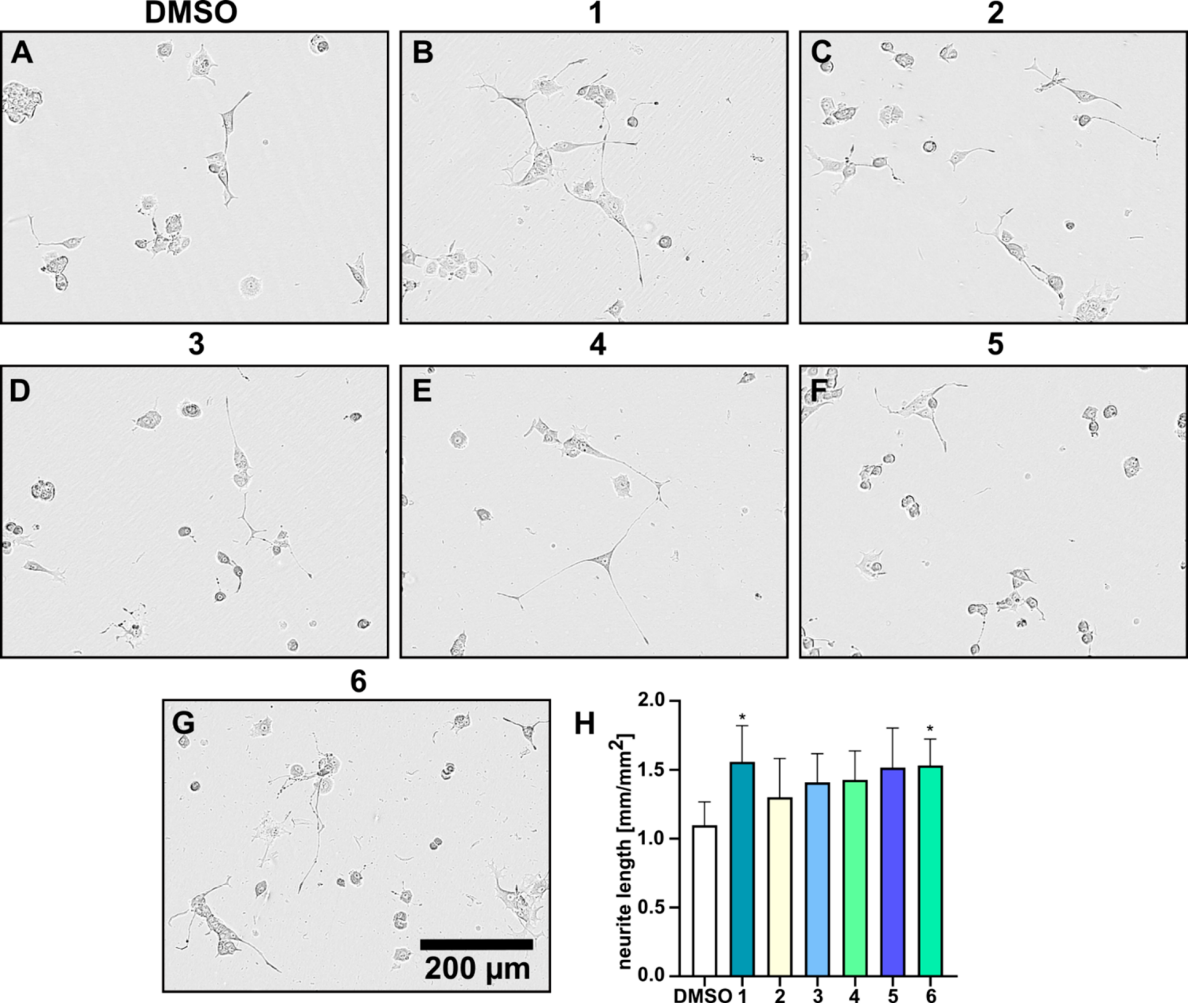
Neurite outgrowth activity of drimane-type sesquiterpenoid compounds at 5 μg/mL. PC-12 cells were treated with 5 ng/mL of NGF and (A) DMSO and (B–G) drimane-type sesquiterpenoid compounds **1**–**6**. Phase contrast images show the neurite outgrowth after 48 h in PC-12 cells. (H) Data shown in bar graph originate from five independent experiments ± s.e.m. *p < 0.1, one-tailed *t*-test.

All the isolated compounds were devoid of any significant activities in both the antimicrobial (12 strains) and cytotoxicity assays (two cell lines). Similar compounds have been reported to exhibit low to no activities in equivalent studies.^27^ Supported by the reported neurite outgrowth activity of other drimane sesquiterpenoids isolated from *Cyathus africanus* and *C. stercoreus*,^8,9^ we decided to assess **1**–**6** in a neurite outgrowth assay in rat pheochromocytoma cells (PC-12), a well-established model system.^7^ To measure the NGF-enhancing activity of compounds **1**–**6**, PC-12 cells were treated with 5 ng/mL of NGF in the presence or absence of the compound at a concentration of 5 μg/mL, respectively, and the mean neurite length was measured after 48 h of treatment (Figure 9). Compounds **7** and **8** were not tested due to inadequate isolated amounts.

Compounds **1** and **6** significantly increased neurite outgrowth compared to DMSO-treated cells. In addition, treatment with compounds **2**–**5** increased the neurite outgrowth to some extent. We hereby show that drimane-type sesquiterpenoid compounds have an NGF-enhancing effect, highlighting their neurotrophic potential.

Hitherto, we have confirmed the neurotrophic potential of drimane-type sesquiterpenoids via stimulation of neural-like PC-12 cell differentiation *in vitro*. The novel structures add to the knowledge base on neurotrophic sesquiterpenoids in addition to those already reported in the literature.^8,9,28^ Apart from the NGF-mediated neurotrophic potential of drimanes reported herein and corroborating previous studies,^8,9^ a drimane-type lactone isolated from *Inonotus obliquus* also induced neuroprotection from H_2_O_2_-induced injury in SH-SY5Y cells.^28^ Thus, the structure–activity relationship of drimane derivatives and their corresponding mechanisms of action in neuroprotection remain to be established. It will be particularly interesting to study whether the molecules act as NGF substitutes and/or induce NGF synthesis, thus aiding in the development of new neuroprotective medicines.

## Experimental Section

### General Experimental Procedures

Optical rotations were recorded on an Anton Paar MCP-150 polarimeter with a sodium D line at 589 nm and 100 mm path length. UV/Vis spectra were obtained using a Shimadzu UV 2450 spectrophotometer. ECD spectra were recorded with a Jasco J-815 spectropolarimeter.

NMR spectra were recorded in methanol-*d*_4_ using Avance III 500 (Bruker, ^1^H 500 MHz, ^13^C 125 MHz) or Avance III 700 (Bruker, ^1^H 700 MHz, ^13^C 175 MHz) spectrometers referenced to the residual solvent peak (3.31 and 49.00 ppm for ^1^H and ^13^C NMR, respectively). Multiplicities of carbon signals were determined from multiplicity edited DEPT-HSQC.

HPLC-DAD/MS was obtained by using an amaZon speed ETD ion trap mass spectrometer (Bruker Daltonics) in positive and negative ionization modes. HR-(+) ESIMS spectra were recorded by using a maXis ESI-TOF (time-of-flight) mass spectrometer (Bruker) connected to an Agilent 1260 series HPLC-UV system equipped with a C18 Acquity UPLC BEH column (Waters). The solvent phase consisted of solvent A (deionized H_2_O + 0.1% formic acid [FA] [v/v]) and solvent B (acetonitrile [MeCN] + 0.1% FA [v/v]) and a separation gradient of 5% B for 0.5 min, 5–100% B over 19.5 min, and holding at 100% B for 5 min with a flow rate of 0.6 mL/min at 40 °C and UV/Vis detection at 200–600 nm. Molecular formulas of the detected compounds were calculated using the Smart Formula algorithm of Compass Data Analysis software (Bruker, version 4.4). Analytical HPLC was performed using a Dionex UltiMate 3000 UHPLC (Thermo Fisher Scientific Inc.) equipped with a C18 Acquity UPLC BEH column (Waters) using the same solvents, gradient system, flow rate, and UV/Vis detection as in HPLC-HRESIMS.

Solvents and chemicals were sourced from AppliChem GmbH, Avantor Performance Materials, Carl Roth GmbH & Co. KG, and Merck. Deionized water was prepared using a Purelab flex water purification system (Veolia Water Technologies).

### Fungal Specimen

The fruiting bodies of *A. violaceus* MUCL 56355 were collected at Mt. Elgon, Kenya, in April 2017, on a dead fallen trunk, by one of the authors (C.D.). The mycelial cultures were established from the basiomata flesh immediately after collection and maintained on YMG media (yeast extract [4 g], glucose [4 g], malt extract [10 g], and agar [20 g] in 1 L of deionized water). The herbarium specimen and the corresponding mycelial cultures were deposited at the BCCM/MUCL (Mycothéque de l’Université Catholique de Louvain, Louvain-la-Neuve, Belgium), under the designation number MUCL 56355. The fungus was identified by morphological methods and molecular sequencing of the LSU and ITS rDNA (comprising the 5.8S region and the internal transcribed spacers ITS1 and ITS2) rRNA genes.^29^ The genomic DNA sequences for the ITS and LSU loci can be retrieved from GenBank under accession numbers FJ411100 and FJ393867, respectively.

### Fungal Culture Preparation, Fermentation, and Extraction of Metabolites

Sterile agar plates consisting of YMG medium were used to inoculate the initial basidiomata pieces and subsequent subculturing to establish axenic mycelial cultures.

Ten plugs (5 mm diameter each) of fully grown *A. violaceus* MUCL 56355 mycelia on YMG agar plates were inoculated into 10 × 500 mL Erlenmeyer culture flasks, containing sterile rice media (composed of 90 g of rice in 90 mL of deionized water). These were incubated at 24 °C in the dark for 68 days, after which the secondary metabolites were extracted. Fungal cultures were extracted by initially soaking them overnight in acetone with mild shaking at 120 rpm. The residue was then separated from acetone by filtration and the solvent evaporated. The semidried extract was reconstituted in distilled water and partitioned with an equal amount of ethyl acetate according to the protocol by Chepkirui et al. (2018).^30^ The aqueous phase was extracted thrice and discarded, whereas the organic phase was filtered through anhydrous Na_2_SO_4_ and evaporated under reduced pressure on a rotary evaporator (Heidolph), to yield 4.0 g of dried extract.

### Isolation and Physicochemical Properties of **1**–**8**

The HPLC-DAD/MS analysis of the ethyl acetate extract obtained from *A. violaceus* MUCL 56355 fermentation in rice media revealed the presence of hitherto undescribed secondary metabolites. The total extract (4.0 g) was fractionated using preparative HPLC (PLC 2020; Gilson). Deionized H_2_O + 0.1% FA (v/v) (solvent A) and MeCN + 0.1% FA (v/v) (solvent B) were used as the eluents with a C_18_ VP-Nucleodur column 100-5 (250 × 40 mm, 7 μm: Machery-Nagel), a flow rate of 40 mL/min, and UV detection at 190, 210, and 280 nm.

Elution occurred as follows: 5% B for 10 min, 5–10% B over 10 min, 10–65% B over 35 min, 65–100% B over 5 min, and a final hold at 100% B for 5 min to yield **1** (12.6 mg, *t*_R_ = 49 min), **2** (10.2 mg, *t*_R_ = 50 min), **3** (10.6 mg, *t*_R_ = 48 min), **4** (8.8 mg, *t*_R_ = 53 min), **5** (10.5 mg, *t*_R_ = 43 min), **6** (7.2 mg, *t*_R_ = 37 min), **7** (2.98 mg, *t*_R_ = 46 min), and **8** (0.71 mg, *t*_R_ = 47 min).

Compound **3** (10.6 mg) was further purified using a semipreparative RP-HPLC Vanquish Core HPLC system (Thermo Fisher Scientific) equipped with a VP Nucleodur 100-5 C18ec (250 × 10 mm, 5 μm: Machery-Nagel) stationary phase. The same eluents were used at a flow rate of 4 mL/min with UV detection at 210 nm. Elution occurred as follows: 5% for 10 min, 5–30% B over 3 min, 30–34% B over 35 min, 34–100% B over 2 min, and a final hold for 5 min at 100% B to yield pure **3** (4.7 mg, *t*_R_ = 24–26 min).

#### Abundisporin A (**1**):

brown solid; [α]^20^_D_ −6 (*c* 0.1, MeOH); UV/Vis (MeOH) λ_max_ (log ε) = 202.0 (0.6) nm; ECD (MeOH, λ [nm] (Δε), *c* = 3.75 mM) 220 (+0.30), 200 (−1.17); ^1^H NMR (CD_3_OD, 500 MHz) and ^13^C NMR (CD_3_OD, 125 MHz) see Table 1; HR-(+)ESIMS *m*/*z* 249.1485 [M – H_2_O + H]^+^ (calcd 249.1485 for C_15_H_21_O_3_^+^), 289.1406 [M + Na]^+^ (calcd 289.1410 for C_15_H_22_NaO_4_^+^), 555.2925 [2M + Na]^+^ (calcd 555.2928 for C_30_H_44_NaO_8_^+^); *t*_R_ = 6.40 min.

#### Abundisporin B (**2**):

brown solid; [α]^20^_D_ +7(*c* 0.1, MeOH); UV/Vis (MeOH) λ_max_ (log ε) = 216.0 (0.9) nm; ECD (MeOH, λ [nm] (Δε), *c* = 3.73 mM) 313 (−0.15), 233 (+0.78); ^1^H NMR (CD_3_OD, 500 MHz) and ^13^C NMR (CD_3_OD, 125 MHz) see Table 1; HR-(+)ESIMS *m*/*z* 251.1275 [M – H_2_O + H]^+^ (calcd 251.1278 for C_14_H_19_O_4_^+^), 269.1379 [M + H]^+^ (calcd 269.1384 for C_14_H_21_O_5_^+^), 291.1199 [M + Na]^+^ (calcd 291.1203 for C_14_H_20_NaO_5_^+^), 537.2693 [2M + H]^+^ (calcd 537.2694 for C_28_H_41_O_10_^+^), 559.2511 [2M + Na]^+^ (calcd 559.2514 for C_28_H_40_NaO_10_^+^); *t*_R_ = 6.27 min.

#### Abundisporin C (**3**):

brown oil; [α]^20^_D_ +38 (*c* 0.1, MeOH); UV/Vis (MeOH) λ_max_ (log ε) = 200.5 (0.7) nm; ECD (MeOH, λ [nm] (Δε), *c* = 3.93 mM) 233 (+0.12), 194 (+2.53); ^1^H NMR (CD_3_OD, 500 MHz) and ^13^C NMR (CD_3_OD, 125 MHz) see Table 1; HR-(+)ESIMS *m*/*z* 277.1405 [M + Na]^+^ (calcd 277.1410 for C_14_H_22_NaO_4_^+^), 531.2927 [2M + Na]^+^ (calcd 531.2932 for C_28_H_44_NaO_8_^+^); *t*_R_ = 6.24 min.

#### Abundisporin D (**4**):

brown oil; [α]^20^_D_ −20 (*c* 0.1, MeOH); UV/Vis (MeOH) λ_max_ (log ε) = 233.0 (0.6) nm; ECD (MeOH, λ [nm] (Δε), *c* = 3.73 mM) 336 (+0.23), 238 (−1.67), 211 (+1.10); ^1^H NMR (CD_3_OD, 500 MHz) and ^13^C NMR (CD_3_OD, 125 MHz) see Table 1; HR-(+)ESIMS *m*/*z* 251.1274 [M – H_2_O + H]^+^ (calcd 251.1278 for C_14_H_19_O_4_^+^), 269.1381 [M + H]^+^ (calcd 269.1384 for C_14_H_21_O_5_^+^), 291.1200 [M + Na]^+^ (calcd 291.1203 for C_14_H_20_NaO_5_^+^), 559.2511 [2M + Na]^+^ (calcd 559.2514 for C_28_H_40_NaO_10_^+^); *t*_R_ = 3.26 min.

#### Abundisporin E (**5**):

brown solid; [α]^20^_D_ −12 (*c* 0.1, MeOH); UV/Vis (MeOH) λ_max_ (log ε) = 224.5 (0.5), 201.5 (0.6) nm; ECD (MeOH, λ [nm] (Δε), *c* = 3.81 mM) 324 (+0.07), 258sh (−0.24), 237 (−0.27), 216 (+0.19); ^1^H NMR (CD_3_OD, 500 MHz) and ^13^C NMR (CD_3_OD, 125 MHz) see Table 2; HR-(+)ESIMS *m*/*z* 263.1277 [M + H]^+^ (calcd 263.1278 for C_15_H_19_O_4_^+^), 525.2482 [2M + H]^+^ (calcd 525.2482 for C_30_H_37_O_8_^+^), 285.1096 [M + Na]^+^ (calcd 285.1097 for C_15_H_18_NaO_4_^+^), 547.2303 [2M + Na]^+^ (calcd 5547.2302 for C_30_H_36_NaO_8_^+^); *t*_R_ = 8.32 min.

#### Abundisporin F (**6**):

brown solid; [α]^20^_D_ +218 (*c* 0.1, MeOH); UV/Vis (MeOH) λ_max_ (log ε) = 202.5 (0.5) nm; ECD (MeOH, λ [nm] (Δε), *c* = 3.75 mM) 307 (−2.84), 221 (+1.64); ^1^H NMR (CD_3_OD, 500 MHz) and ^13^C NMR (CD_3_OD, 125 MHz) see Table 2; HR-(+)ESIMS *m*/*z* 249.1483 [M – H_2_O + H]^+^ (calcd 249.1485 for C_15_H_21_O_3_^+^), 289.1406 [M + Na]^+^ (calcd 289.1434 for C_15_H_22_NaO_4_^+^), 555.2925 [2M + Na]^+^ (calcd 555.2928 for C_30_H_44_NaO_8_^+^); *t*_R_ = 6.59 min.

#### Abundisporin G (**7**):

brown solid; [α]^20^_D_ +189 (*c* 0.1, MeOH); UV/Vis (MeOH) λ_max_ (log ε) = 210.0 (0.8) nm; ECD (MeOH, λ [nm] (Δε), *c* = 3.22 mM) 322 (+0.05), 241 (−0.90), 211 (+0.03), 203 (−0.63); ^1^H NMR (CD_3_OD, 500 MHz) and ^13^C NMR (CD_3_OD, 125 MHz) see Table 2; HR-(+)ESIMS *m*/*z* 293.1374 [M – H_2_O + H]^+^ (calcd 293.1374 for C_16_H_21_O_5_^+^), 311.1487 [M + H]^+^ (calcd 311.1489 for C_16_H_23_O_6_^+^), 333.1301 [M + Na]^+^ (calcd 333.1309 for C_16_H_22_NaO_6_^+^), 621.2901 [2M + H]^+^ (calcd 621.2905 for C_32_H_45_O_12_^+^), 643.2714 [2M + Na]^+^ (calcd 643.2714 for C_32_H_44_NaO_12_^+^); *t*_R_ = 7.28 min.

#### Abundisporin H (**8**):

brown solid; [α]^20^_D_ −14 (*c* 0.1, MeOH); UV/Vis (MeOH) λ_max_ (log ε) = 241.5 (0.8) nm; ECD (MeOH, λ [nm] (Δε), *c* = 3.59 mM) 356 (+0.21), 249 (−1.22), 213 (+1.56), 193 (−0.56); ^1^H NMR (CD_3_OD, 500 MHz) and ^13^C NMR (CD_3_OD, 125 MHz) see Table 2; HR-(+)ESIMS *m*/*z* 279.1229 [M + H]^+^ (calcd 279.1227 for C_15_H_19_O_5_^+^), 301.1047 [M + Na]^+^ (calcd 301.1046 for C_15_H_18_NaO_5_^+^), 557.2383 [2M + H]^+^ (calcd 557.2381 for C_30_H_37_O_10_^+^), 579.2200 [2M + Na]^+^ (calcd 579.2201 for C_30_H_36_NaO_10_^+^); *t*_R_ = 6.65 min.

### Preparation of (*R*)- and (*S*)-MTPA Ester Derivatives of **1**–**4**

An aliquot of 0.5 mg of each compound was first dissolved in 300 μL of deuterated pyridine-*d*_5_. Subsequently, 4 μL of (*R*)-(−)-α-methoxy-α-(trifluoromethyl)phenylacetyl chloride was added into the solution and left for 1 h at room temperature. The reaction was monitored by analytical HPLC-MS. If necessary, another 2 μL of (*R*)-MTPA was added when HPLC-MS analysis showed that the reaction was not complete. Immediately after reaction completion, the samples were transferred into 3.0 mm NMR tubes. This was followed by measurements of the ^1^H NMR and ^1^H,^1^H–COSY spectra. The same procedure was repeated with a second 0.5 mg aliquot of each compound using (*S*)-(+)-α-methoxy-α-(trifluoromethyl)phenylacetyl chloride. The resulting Δδ^*SR*^ values were calculated and interpreted as previously described.^16,17^

### Antimicrobial Assay

The determination of minimum inhibitory concentration (MIC) of the isolated compounds was carried out in 96-well microtiter plates according to our standard protocol.^31^ The tests were done against a panel of clinically relevant microorganisms (bacteria: *Staphylococcus aureus* [DSM 346], *Bacillus subtilis* [DSM 10], *Acinetobacter baumanii* [DSM 30008], *Escherichia coli* [DSM 1116], *Chromobacterium violaceum* [DSM 30191], *Pseudomonas aeruginosa* [PA14], and *Mycolicibacterium smegmatis* [ATCC 700084] and fungi: *Candida albicans* [DSM 1665], *Mucor hiemalis* [DSM 2656], *Schizosaccharomyces pombe* [DSM 70572], *Rhodotorula glutinis* [DSM 10134], and *Pichia anomala* [DSM 6766]). The metabolites were diluted in methanol in the range 66.7–0.52 μg/mL. The MIC was thereafter recorded as the lowest concentration under which no growth of the aforementioned test strains was visualized, following an overnight incubation. Ciprofloxacin, kanamycin, gentamycin, and oxytetracycline as well as nystatin were used as positive controls against bacterial pathogens and fungi, respectively.

### Cytotoxicity Assay

*In vitro* cytotoxicity (IC_50_) assessments were carried out on the isolated compounds based on an MTT (3-(4,5-dimethylthiayol-2-yl)-2,5-diphenyltetrazolium bromide) test in 96-well plates, using the cell lines KB3.1 (human endocervical adenocarcinoma) and L929 (mouse fibroblasts), in accordance with our previously established methods.^30,31^ Epothilone B was used as a positive control.

### Neurite Outgrowth Assays

Neurite outgrowth assays were performed as previously reported.^7,8,32^ Briefly, PC-12 cells were incubated in growth media (RPMI-1640, 10% heat-inactivated horse serum, 5% fetal bovine serum, 2 mM l-glutamine, 1× penicillin–streptomycin (P/S)) and seeded for neurite outgrowth experiments on collagen type IV (C5533, Sigma-Aldrich)-coated 96-well plates, at a concentration of 1.5 × 10^4^ cells per well. Cells were incubated for 6 h at 37 °C with 7.5% CO_2_ to attach the cells to the culture vessel. Treatment with compounds was initiated by exchanging the culture media with fresh differentiation media (RPMI-1640, 1% heat-inactivated horse serum, 2 mM l-glutamine, and 1× P/S) containing compounds **1**–**6** (5 μg/mL) supplemented with NGF (5 ng/mL). DMSO served as the control. Cells were assessed by phase contrast imaging after 48 h by an IncuCyte S3 live-cell analysis system (Sartorius). Neurite length was determined using the IncuCyte NeuroTrack software module.

### Statistical Analysis

The data obtained from neurite outgrowth assays were analyzed on Prism V8 software (Graph Pad Software Inc.), employing the Student *t*-test statistical method. Data are displayed as the mean ± SEM.

### Computational Section

Mixed torsional/low-mode conformational searches were carried out by means of the Macromodel 10.8.011 software using the MMFF with an implicit solvent model for CHCl_3_ applying a 21 kJ/mol energy window.^33^ Geometry reoptimizations of the resultant conformers [ωB97X/TZVP with the PCM solvent model for MeOH] and TDDFT ECD calculations were performed with Gaussian 09.^34^ For ECD, various functionals (B3LYP, BH&HLYP, CAM-B3LYP, PBE0) and the TZVP basis set were used with the same solvent model as in the preceding DFT optimization step. ECD spectra were generated as the sum of Gaussians with 3600 cm^–1^ half-height widths, using dipole-velocity-computed rotational strengths.^35^ Boltzmann distributions were estimated from the ωB97X energies. The MOLEKEL program was used for visualization of the results.^36^

## Data Availability

The NMR data for **1**–**8** have been deposited in the Natural Products Magnetic Resonance Database (NP-MRD; www.np-mrd.org) and can be found at NP0331915 (**1**), NP0331917 (**2**), NP0331912 (**3**), NP0331914 (**4**), NP0331911 (**5**), NP0331918 (**6**), NP0331913 (**7**), NP0331916 (**8**).

## Supporting Information Available

Photos of *Abundisporus violaceus* MUCL 56355, evolutionary relationship of *A. violaceus* (MUCL 56355) and related Polyporaceae taxa obtained from GenBank, LRESIMS, HRESIMS, 1D (^1^H/^13^C), 2D (^1^H–^1^H COSY, HMBC, HSQC, and ROESY) NMR spectra of compounds **1**–**8** (PDF)
