# Supplementary material for: Neurite Outgrowth-Inducing Drimane-Type Sesquiterpenoids Isolated from Cultures of the Polypore Abundisporus violaceus MUCL 56355
Source: J Nat Prod. 2023 Nov 1;86(11):2457–67. doi: 10.1021/acs.jnatprod.3c00525 (PMC10683085; doi:10.1021/acs.jnatprod.3c00525)
Supplement: Supplementary file 1 — np3c00525_si_001.pdf [file np3c00525_si_001.pdf]

**SUPPORTING INFORMATION FOR**

# Neurite outgrowth-inducing Drimane-type Sesquiterpenoids Isolated from Cultures of the Polypore *Abundisporus violaceus* MUCL 56355

Winnie Chemutai Sum,[†,‡] Sherif S. Ebada,[†,§] Marco Kirchenwitz,[⊥] Lucy Wanga,[‖] Cony Decock,[∇] Theresia E. B. Stradal,[⊥] Josphat Clement Matasyoh,[#] Attila Mándi,[¶] Tibor Kurtán*,[¶] and Marc Stadler*,[†,‡]

[†] Department of Microbial Drugs, Helmholtz Centre for Infection Research GmbH (HZI), Inhoffenstraße 7, 38124 Braunschweig, Germany; winnie.sumchemutai@helmholtz-hzi.de; sherif.elsayed@helmholtz-hzi.de

[‡] Institute of Microbiology, Technische Universität Braunschweig, Spielmannstraße 7, 38106 Braunschweig, Germany

[§] Department of Pharmacognosy, Faculty of Pharmacy, Ain Shams University, 11566 Cairo, Egypt; sherif_elsayed@pharma.asu.edu.eg

[⊥] Department of Cell Biology, Helmholtz Centre for Infection Research, Inhoffenstrasse 7, 38124 Braunschweig, Germany; marco.kirchenwitz@helmholtz-hzi.de; theresia.stradal@helmholtz-hzi.de

[‖] Department of Biochemistry, Egerton University, P.O. Box 536, 20115, Njoro, Kenya; lucywanga15@gmail.com

[∇] Mycothéque de l' Universite Catholique de Louvain (BCCM/MUCL), Place Croix du Sud 3, B-1348 Louvain-la-Neuve, Belgium; cony.decock@uclouvain.be

[#] Department of Chemistry, Egerton University, P.O. Box 536, 20115, Njoro, Kenya; jmatasyoh@egerton.ac.ke

[¶] Department of Organic Chemistry, University of Debrecen, P. O. Box 400, 4002 Debrecen, Hungary; mandi.attila@science.unideb.hu; kurtan.tibor@science.unideb.hu

* Correspondence; kurtan.tibor@science.unideb.hu (T.K.); Marc.Stadler@helmholtz-hzi.de (M.S.) Tel.: +49-531-6181-4240; Fax +49-531-6181-9499

## TABLE OF CONTENTS


| # | Contents | Page |
|---|---|---|
| 1 | Figure S1. *Abundisporus violaceus* fungus growing on the bark of a tree (a) and its mycelial growth on YMG media (b). | S4 |
| 2 | Figure S2. Evolutionary relationship of *A. violaceus* MUCL 56355 and related Polyporaceae taxa obtained from GenBank. | S4 |
| 3 | Figure S3. HPLC chromatogram and LRESIMS spectrum of **1**. | S5 |
| 4 | Figure S4. HPLC chromatogram and HRESIMS spectrum of **1**. | S6 |
| 5 | Figure S5. $^{1}$H NMR spectrum of **1** in methanol-$d_4$ at 500 MHz. | S7 |
| 6 | Figure S6. $^{13}$C NMR spectrum of **1** in methanol-$d_4$ at 125 MHz. | S8 |
| 7 | Figure S7. $^{1}$H-$^{1}$H COSY spectrum of **1** in methanol-$d_4$ at 500 MHz. | S9 |
| 8 | Figure S8. HMBC spectrum of **1** in methanol-$d_4$ at 500 MHz. | S10 |
| 9 | Figure S9. HSQC spectrum of **1** in methanol-$d_4$ at 500 MHz. | S11 |
| 10 | Figure S10. ROESY spectrum of **1** in methanol-$d_4$ at 700 MHz. | S12 |
| 11 | Figure S11. HPLC chromatogram and LRESIMS spectrum of **2**. | S13 |
| 12 | Figure S12. HPLC chromatogram and HRESIMS spectrum of **2**. | S14 |
| 13 | Figure S13. $^{1}$H NMR spectrum of **2** in methanol-$d_4$ at 500 MHz. | S15 |
| 14 | Figure S14. $^{13}$C NMR spectrum of **2** in methanol-$d_4$ at 125 MHz. | S16 |
| 15 | Figure S15. $^{1}$H-$^{1}$H COSY spectrum of **2** in methanol-$d_4$ at 500 MHz. | S17 |
| 16 | Figure S16. HMBC spectrum of **2** in methanol-$d_4$ at 500 MHz. | S18 |
| 17 | Figure S17. HSQC spectrum of **2** in methanol-$d_4$ at 500 MHz. | S19 |
| 18 | Figure S18. ROESY spectrum of **2** in methanol-$d_4$ at 500 MHz. | S20 |
| 19 | Figure S19. HPLC chromatogram and LRESIMS spectrum of **3**. | S21 |
| 20 | Figure S20. HPLC chromatogram and HRESIMS spectrum of **3**. | S22 |
| 21 | Figure S21. $^{1}$H NMR spectrum of **3** in methanol-$d_4$ at 500 MHz. | S23 |
| 22 | Figure S22. $^{13}$C NMR spectrum of **3** in methanol-$d_4$ at 125 MHz. | S24 |
| 23 | Figure S23. $^{1}$H-$^{1}$H COSY spectrum of **3** in methanol-$d_4$ at 500 MHz. | S25 |
| 24 | Figure S24. HMBC spectrum of **3** in methanol-$d_4$ at 500 MHz. | S26 |
| 25 | Figure S25. HSQC spectrum of **3** in methanol-$d_4$ at 500 MHz. | S27 |
| 26 | Figure S26. ROESY spectrum of **3** in methanol-$d_4$ at 500 MHz. | S28 |
| 27 | Figure S27. HPLC chromatogram and LRESIMS spectrum of **4**. | S29 |
| 28 | Figure S28. HPLC chromatogram and HRESIMS spectrum of **4**. | S30 |
| 29 | Figure S29. $^{1}$H NMR spectrum of **4** in methanol-$d_4$ at 500 MHz. | S31 |
| 30 | Figure S30. $^{13}$C NMR spectrum of **4** in methanol-$d_4$ at 125 MHz. | S32 |
| 31 | Figure S31. $^{1}$H-$^{1}$H COSY spectrum of **4** in methanol-$d_4$ at 500 MHz. | S33 |
| 32 | Figure S32. HMBC spectrum of **4** in methanol-$d_4$ at 500 MHz. | S34 |
| 33 | Figure S33. HSQC spectrum of **4** in methanol-$d_4$ at 500 MHz. | S35 |
| 34 | Figure S34. ROESY spectrum of **4** in methanol-$d_4$ at 500 MHz. | S36 |
| 35 | Figure S35. Low-energy ωB97X/TZVP PCM/MeOH conformers of (1*S*,4*R*,5*R*,10*R*)-**4**. | S37 |
| 36 | Figure S36. Experimental ECD spectrum of **4** (black) compared with the CAM-B3LYP/TZVP PCM/MeOH ECD spectra of the lowest-energy conformers of the two distinct conformer clusters of (1*S*,4*R*,5*R*,10*R*)-**4** (red: conformer A, blue: conformer B). Level of DFT optimization: ωB97X/TZVP PCM/MeOH. Bars represent the computed rotational strength values for conformers A (red) and B (blue). | S38 |
| 37 | Figure S37. HPLC chromatogram and LRESIMS spectrum of **5**. | S39 |
| 38 | Figure S38. HPLC chromatogram and HRESIMS spectrum of **5**. | S40 |
| 39 | Figure S39. $^{1}$H NMR spectrum of **5** in methanol-$d_4$ at 500 MHz. | S41 |
| 40 | Figure S40. $^{13}$C NMR spectrum of **5** in methanol-$d_4$ at 125 MHz. | S42 |
| 41 | Figure S41. $^{1}$H-$^{1}$H COSY spectrum of **5** in methanol-$d_4$ at 500 MHz. | S43 |
| 42 | Figure S42. HMBC spectrum of **5** in methanol-$d_4$ at 500 MHz. | S44 |
| 43 | Figure S43. HSQC spectrum of **5** in methanol-$d_4$ at 500 MHz. | S45 |

| | | |
|---|---|---|
| **44** | Figure S44. ROESY spectrum of **5** in methanol-$d_4$ at 700 MHz. | **S46** |
| **45** | Figure S45. Low-energy ωB97X/TZVP PCM/MeOH conformers of (4*R*,5*R*,10*S*)-**5**. | **S47** |
| **46** | Figure S46. HPLC chromatogram and LRESIMS spectrum of **6**. | **S48** |
| **47** | Figure S47. HPLC chromatogram and HRESIMS spectrum of **6**. | **S49** |
| **48** | Figure S48. $^1$H NMR spectrum of **6** in methanol-$d_4$ at 500 MHz. | **S50** |
| **49** | Figure S49. $^{13}$C NMR spectrum of **6** in methanol-$d_4$ at 125 MHz. | **S51** |
| **50** | Figure S50. $^1$H-$^1$H COSY spectrum of **6** in methanol-$d_4$ at 500 MHz. | **S52** |
| **51** | Figure S51. HMBC spectrum of **6** in methanol-$d_4$ at 500 MHz. | **S53** |
| **52** | Figure S52. HSQC spectrum of **6** in methanol-$d_4$ at 500 MHz. | **S54** |
| **53** | Figure S53. ROESY spectrum of **6** in methanol-$d_4$ at 700 MHz. | **S55** |
| **54** | Figure S54. Low-energy ωB97X/TZVP PCM/MeOH conformers of (4*R*,5*R*,9*S*,10*S*)-**6**. | **S56** |
| **55** | Figure S55. HPLC chromatogram and LRESIMS spectrum of **7**. | **S57** |
| **56** | Figure S56. HPLC chromatogram and HRESIMS spectrum of **7**. | **S58** |
| **57** | Figure S57. $^1$H NMR spectrum of **7** in methanol-$d_4$ at 500 MHz. | **S59** |
| **58** | Figure S58. $^{13}$C NMR spectrum of **7** in methanol-$d_4$ at 125 MHz. | **S60** |
| **59** | Figure S59. $^1$H-$^1$H COSY spectrum of **7** in methanol-$d_4$ at 500 MHz. | **S61** |
| **60** | Figure S60. HMBC spectrum of **7** in methanol-$d_4$ at 500 MHz. | **S62** |
| **61** | Figure S61. HSQC spectrum of **7** in methanol-$d_4$ at 500 MHz. | **S63** |
| **62** | Figure S62. ROESY spectrum of **7** in methanol-$d_4$ at 500 MHz. | **S64** |
| **63** | Figure S63. Low-energy ωB97X/TZVP PCM/MeOH conformers of (4*R*,5*R*,9*R*,10*S*)-**7**. | **S65** |
| **64** | Figure S64. HPLC chromatogram and LRESIMS spectrum of **8**. | **S66** |
| **65** | Figure S65. HPLC chromatogram and HRESIMS spectrum of **8**. | **S67** |
| **66** | Figure S66. $^1$H NMR spectrum of **8** in methanol-$d_4$ at 500 MHz. | **S68** |
| **67** | Figure S67. $^{13}$C NMR spectrum of **8** in methanol-$d_4$ at 125 MHz. | **S69** |
| **68** | Figure S68. $^1$H-$^1$H COSY spectrum of **8** in methanol-$d_4$ at 500 MHz. | **S70** |
| **69** | Figure S69. HMBC spectrum of **8** in methanol-$d_4$ at 500 MHz. | **S71** |
| **70** | Figure S70. HSQC spectrum of **8** in methanol-$d_4$ at 500 MHz. | **S72** |
| **71** | Figure S71. ROESY spectrum of **8** in methanol-$d_4$ at 700 MHz. | **S73** |
| **72** | Figure S72. Low-energy ωB97X/TZVP PCM/MeOH conformers of (4*R*,5*R*,10*S*)-**8**. | **S74** |
| **73** | Figure S73. $^1$H NMR spectrum of **1** in pyridine-$d_5$ at 500 MHz. | **S75** |
| **74** | Figure S74. $^1$H NMR spectrum of 11-*O*-(*S*)-MTPA ester of **1** in pyridine-$d_5$ at 500 MHz. | **S76** |
| **75** | Figure S75. $^1$H NMR spectrum of 11-*O*-(*R*)-MTPA ester of **1** in pyridine-$d_5$ at 500 MHz. | **S77** |
| **76** | Figure S76. $^1$H NMR spectrum of **2** in pyridine-$d_5$ at 500 MHz. | **S78** |
| **77** | Figure S77. $^1$H NMR spectrum of 11-*O*-(*S*)-MTPA ester of **2** in pyridine-$d_5$ at 500 MHz. | **S79** |
| **78** | Figure S78. $^1$H NMR spectrum of 11-*O*-(*R*)-MTPA ester of **2** in pyridine-$d_5$ at 500 MHz. | **S80** |
| **79** | Figure S79. $^1$H NMR spectrum of **3** in pyridine-$d_5$ at 500 MHz. | **S81** |
| **80** | Figure S80. $^1$H NMR spectrum of 11-*O*-(*S*)-MTPA ester of **3** in pyridine-$d_5$ at 500 MHz. | **S82** |
| **81** | Figure S81. $^1$H NMR spectrum of 11-*O*-(*R*)-MTPA ester of **3** in pyridine-$d_5$ at 500 MHz. | **S83** |
| **82** | Figure S82. $^1$H NMR spectrum of **4** in pyridine-$d_5$ at 500 MHz. | **S84** |
| **83** | Figure S83. $^1$H NMR spectrum of 11-*O*-(*S*)-MTPA ester of **4** in pyridine-$d_5$ at 500 MHz. | **S85** |
| **84** | Figure S84. $^1$H NMR spectrum of 11-*O*-(*R*)-MTPA ester of **4** in pyridine-$d_5$ at 500 MHz. | **S86** |

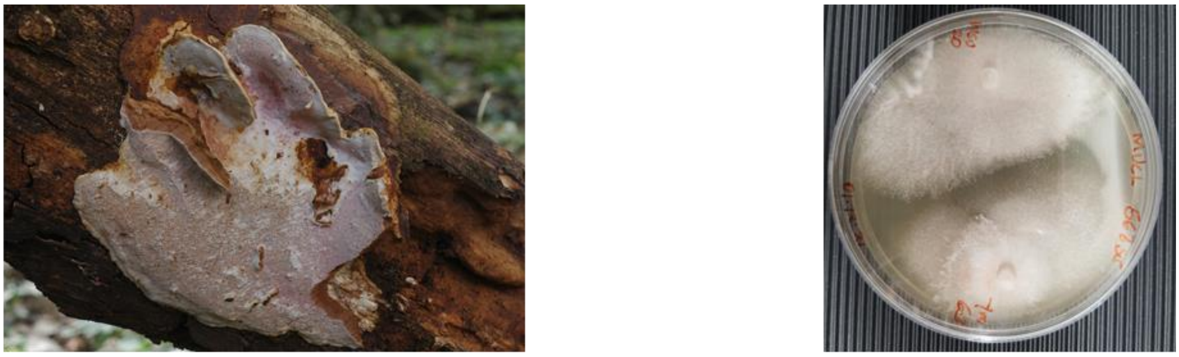

a b

Figure S1. *Abundisporus violaceus* fungus growing on the bark of a tree (a) and its mycelial growth on YMG media (b).

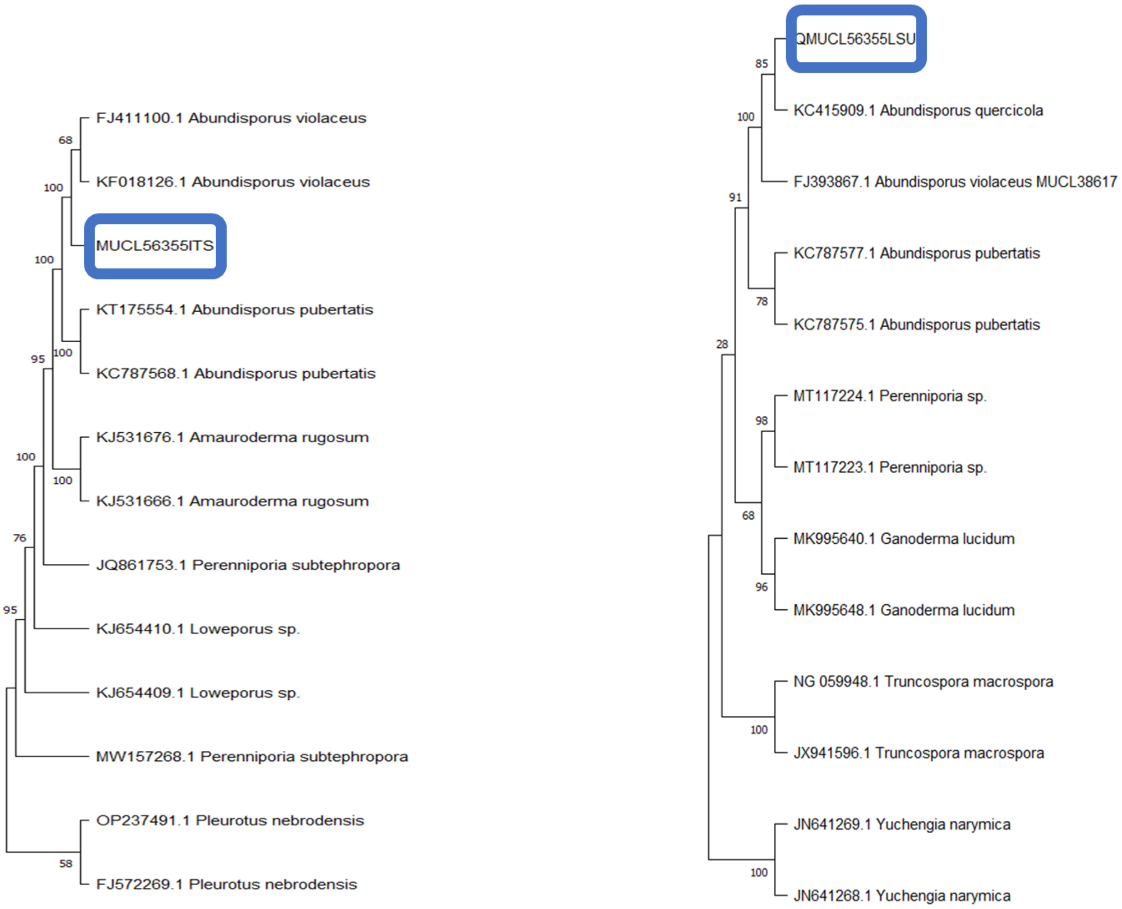


a b

Figure S2. Evolutionary relationship of *A. violaceus* MUCL 56355 and related polyporaceae taxa obtained from GenBank. This was inferred using Neighbor-Joining method (1) and the analyses involved 13 nucleotide sequences each: (a) ITS and (b) LSU sequences. The percentage of replicate trees in which the associated taxa clustered together in the bootstrap test (100 replicates) are shown next to the branches (2). The evolutionary distances were computed using the Maximum Composite Likelihood method (3) and are in the units of the number of base substitutions per site. The evolutionary analyses were conducted in MEGA11 (4).

# Generic Display Report

| | | | |
|---|---|---|---|
| **Analysis Info** | | Acquisition Date | 02.03.2022 18:27:45 |
| Analysis Name | \\Neon\MWISCOM\PEOPLE\sel22_Sherif Elsayed\Abundisporus\Amazon\A. violaceus AmaZon\Abund B1F9F2_BB6_01_10300.d | | |
| Method | Screening_Set_05_iso-uv210_10300.m | Operator | lab |
| Sample Name | Abund B1F9F2 | Instrument | amaZon speed |
| Comment | | | |

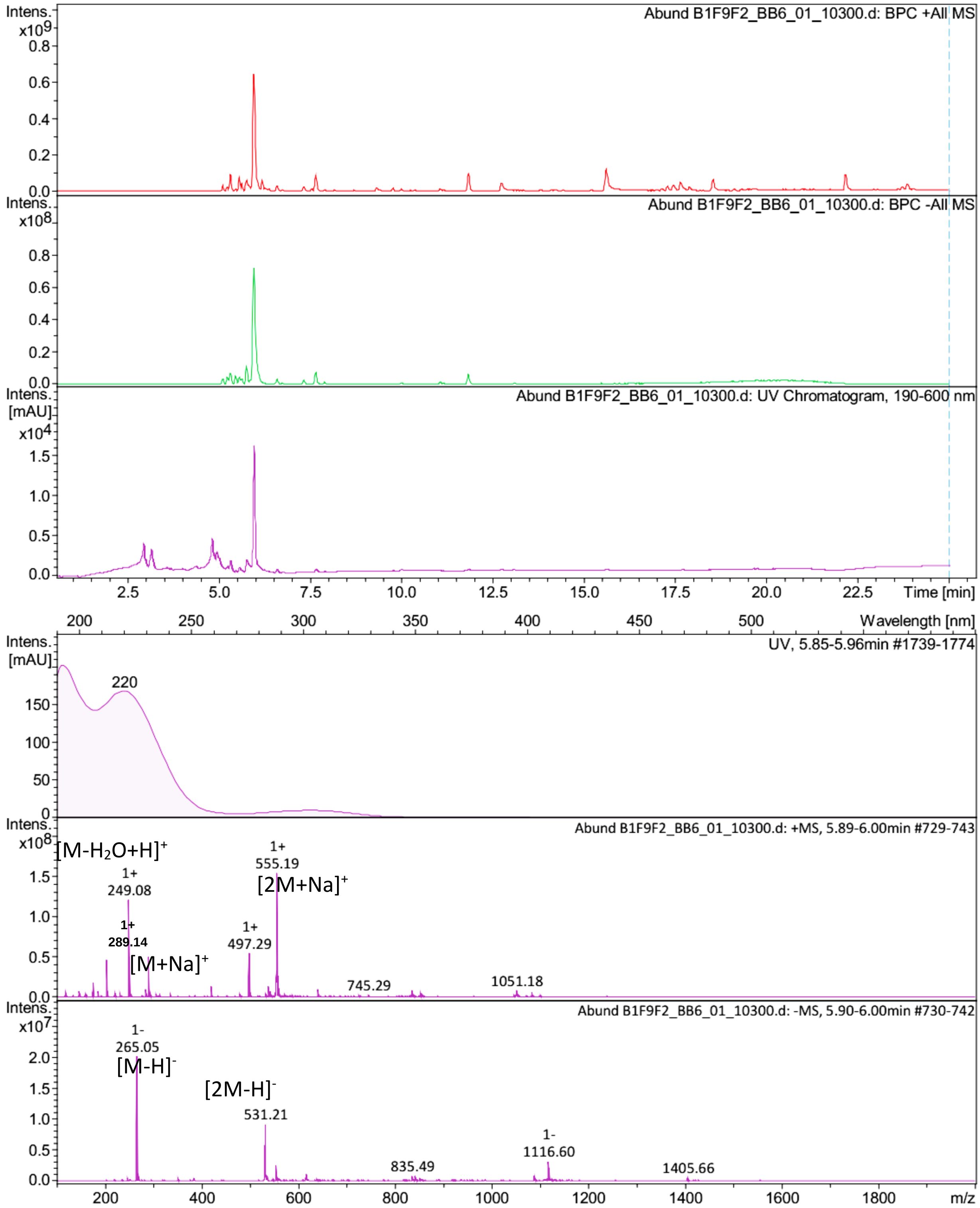




Figure S3. LRESIMS of **1**.

# Generic Display Report

**Analysis Info**

| | | | |
|---|---|---|---|
| Analysis Name | \\Neon\MWISCOM\PEOPLE\sel22_Sherif Elsayed\Abundisporus\Maxis\A. violaceus MaXis\Abund B2F11_P1-B-5_01_9622.d | Acquisition Date | 28.02.2022 15:24:35 |
| Method | [illegible]ng_ms_100_2500_line.m | Operator | ate06 |
| Sample Name | Abund B2F11 | Instrument | maXis |
| Comment | Screening01<br>Waters Acquity UPLC BEH C_18 1,7um 2.1x50mm | | |

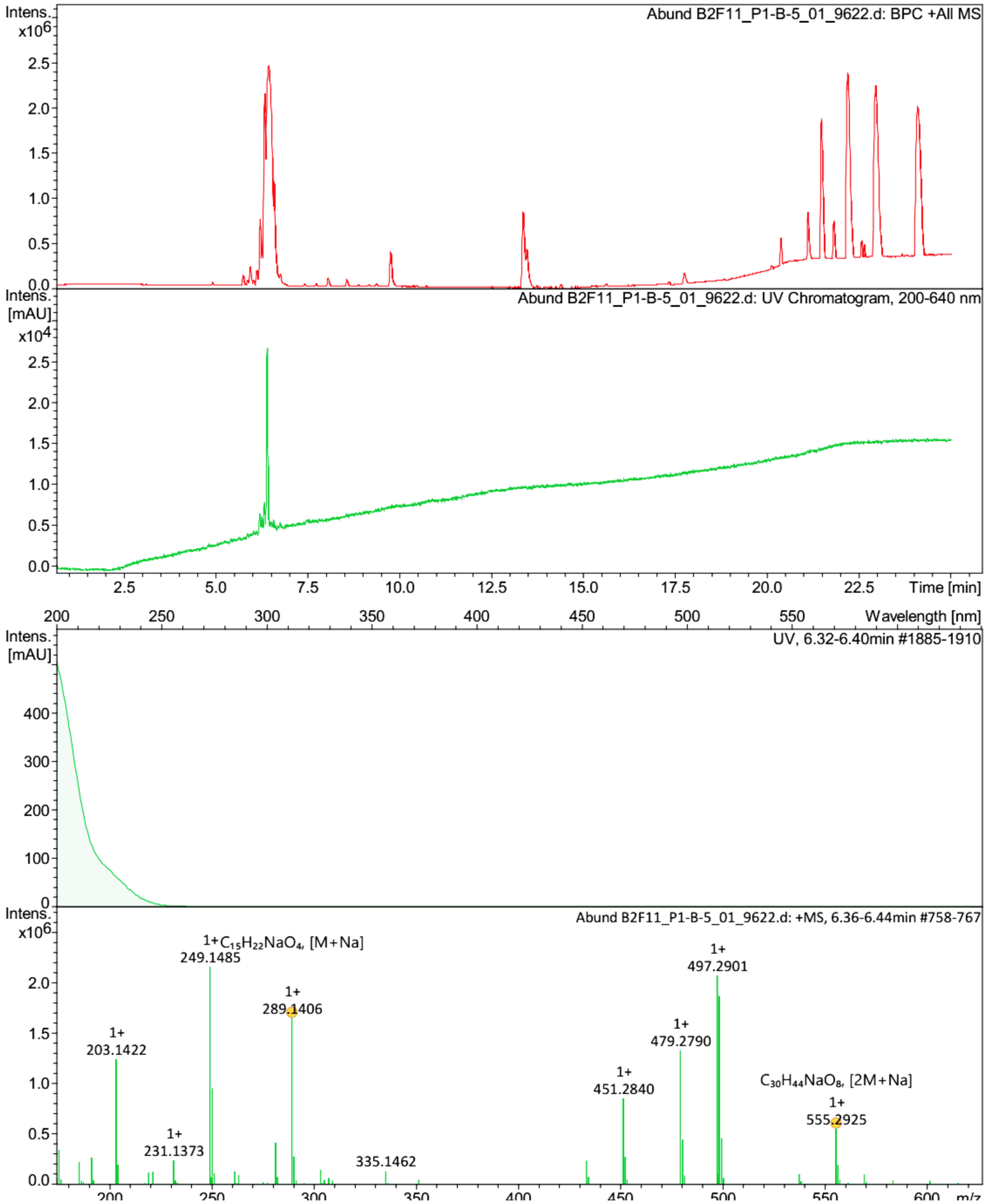




Figure S4. HRESIMS of **1**.

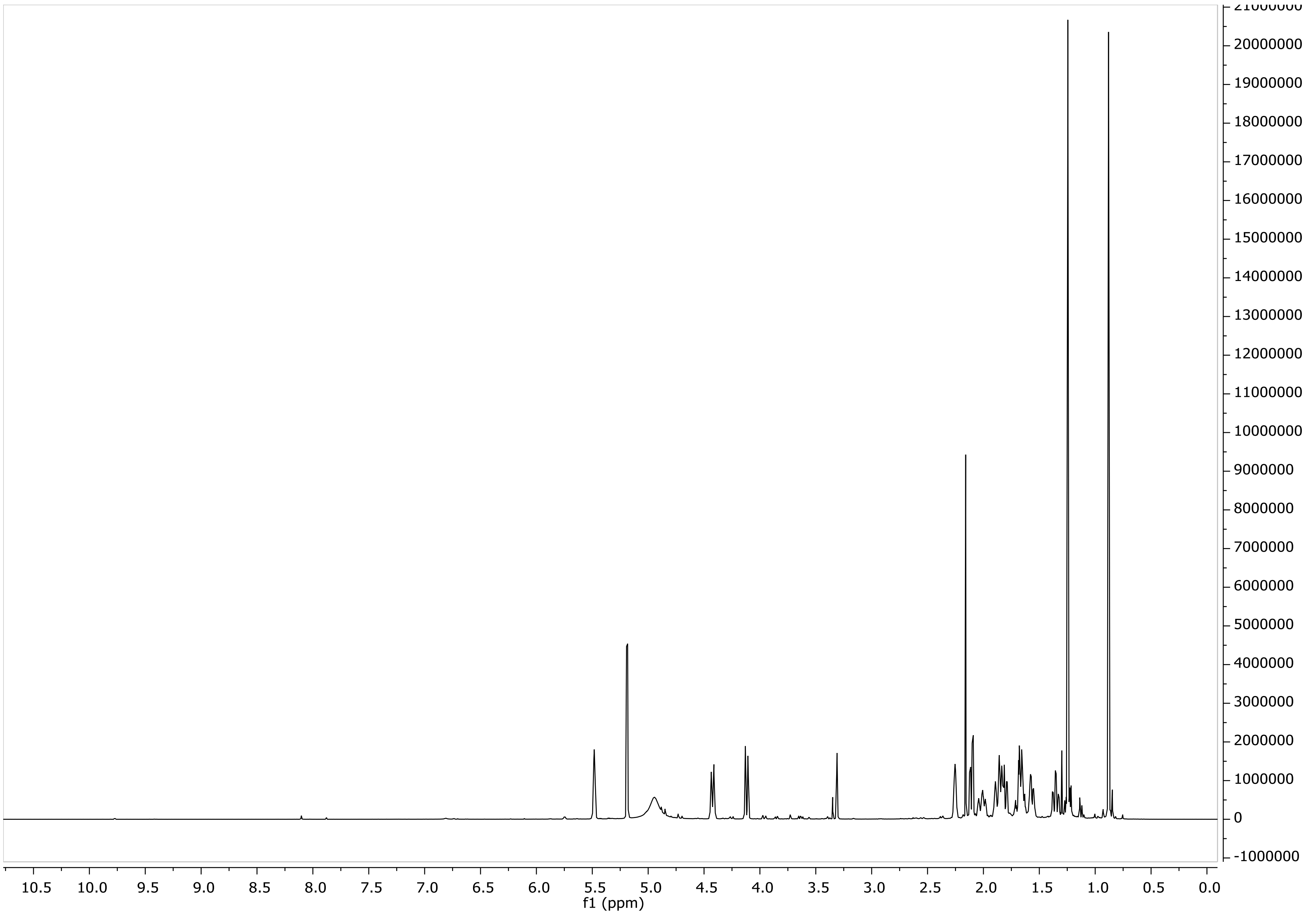


Figure S5. $^{1}$H NMR spectrum of **1** in methanol-$d_4$ at 500 MHz.

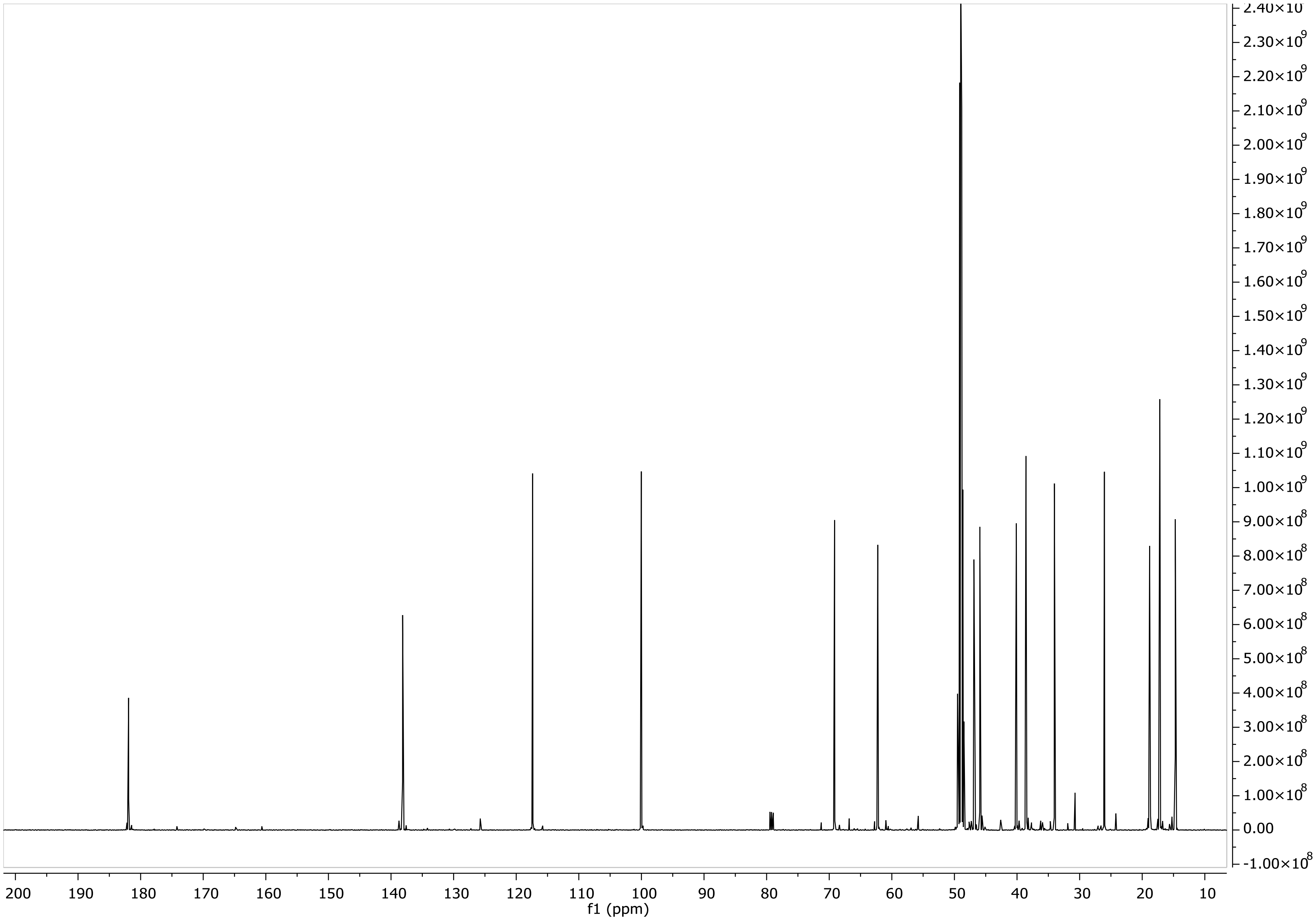


Figure S6. $^{13}C$ NMR spectrum of **1** in methanol-$d_4$ at 125 MHz.

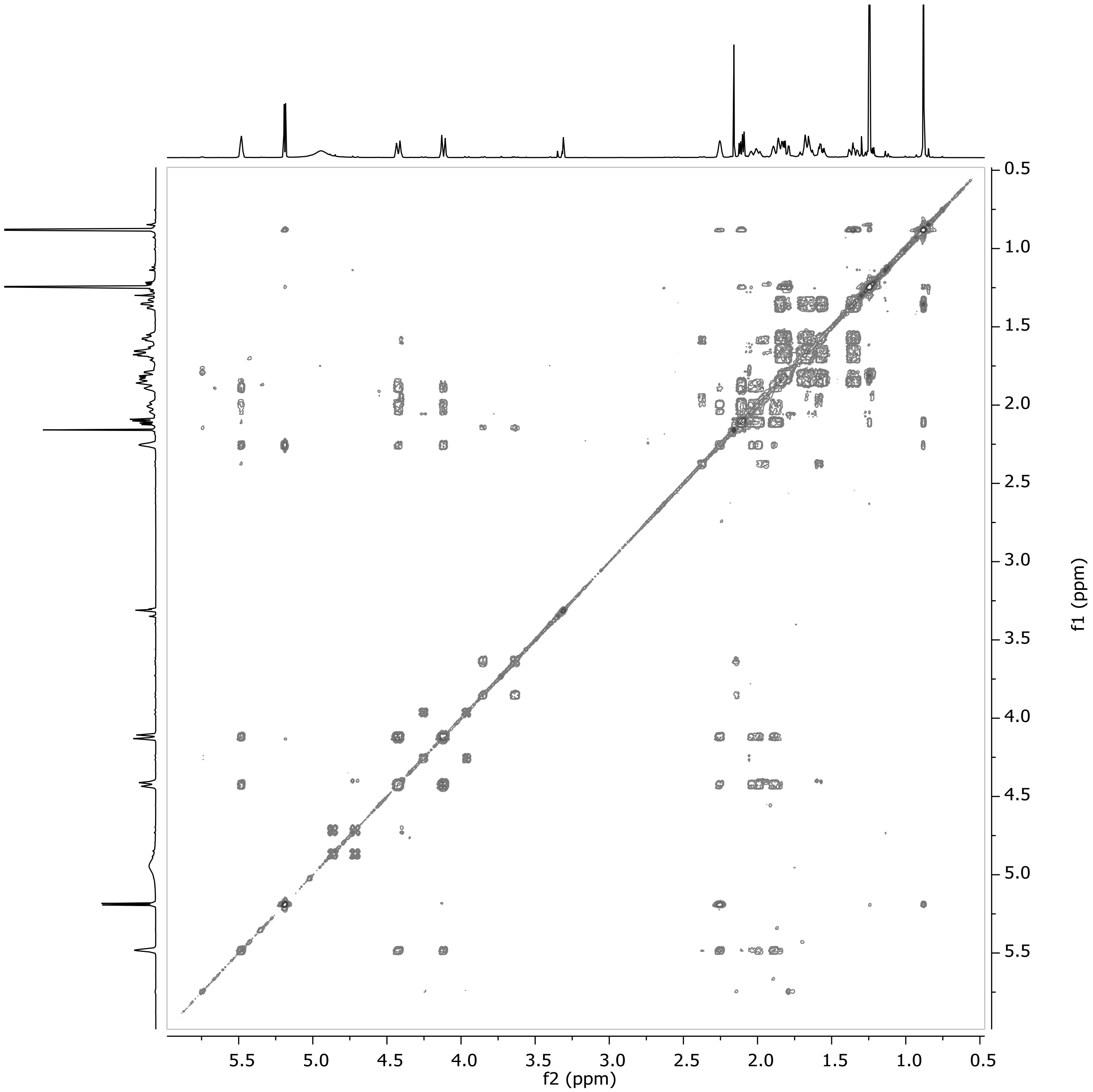


Figure S7. $^{1}$H-$^{1}$H COSY spectrum of **1** in methanol-$d_4$ at 500 MHz.

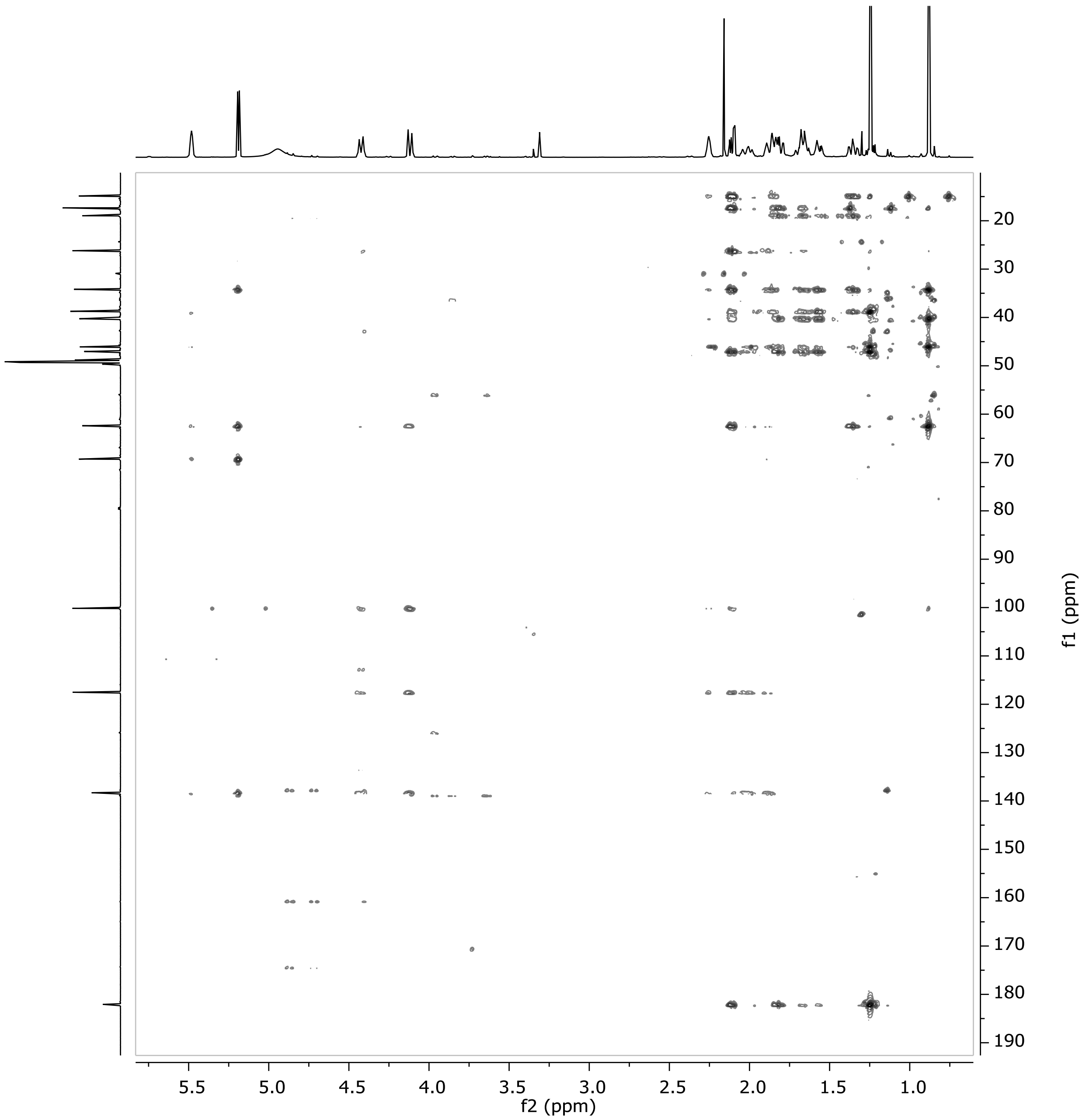


Figure S8. HMBC spectrum of **1** in methanol-$d_4$ at 500 MHz.

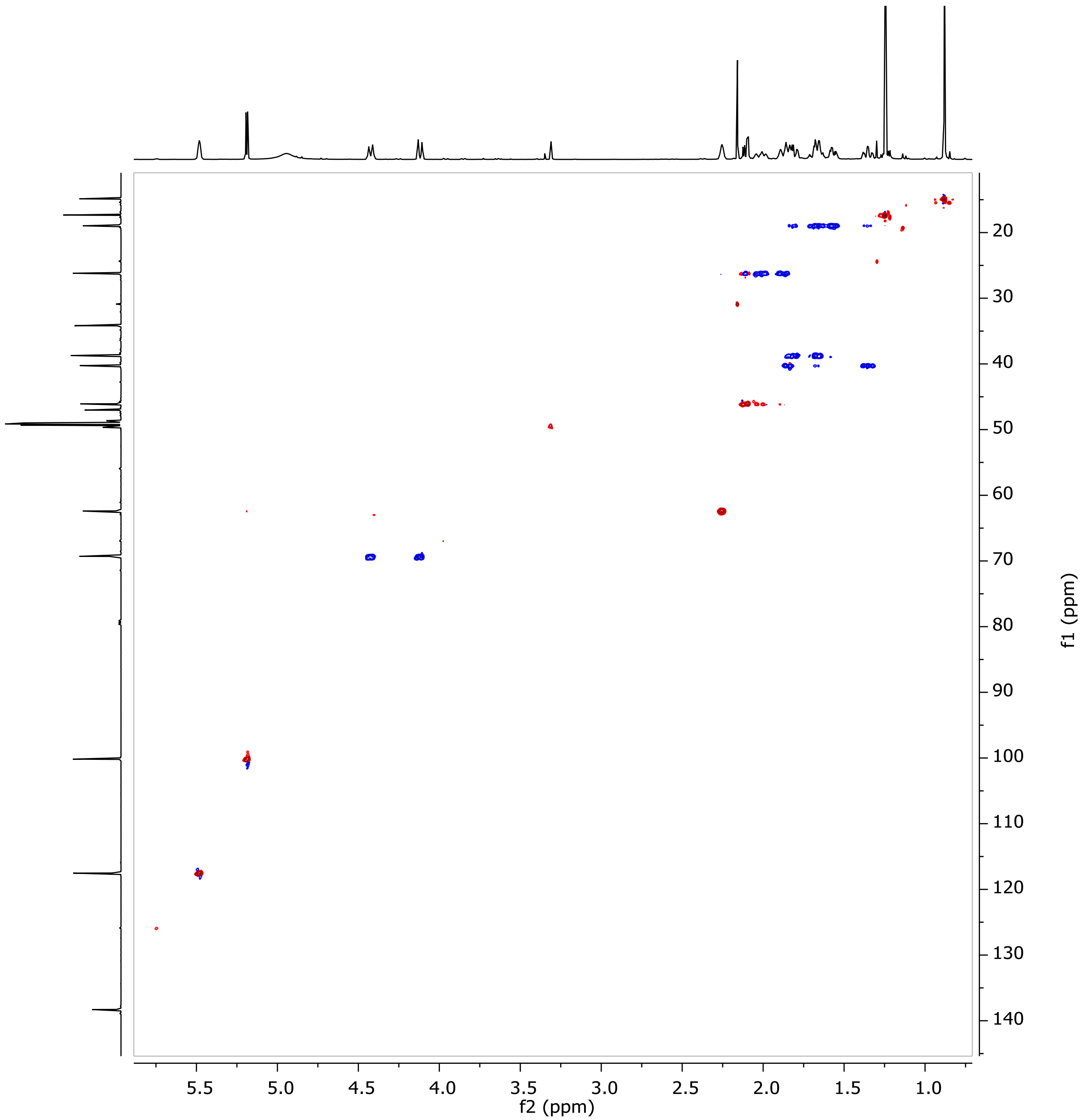


Figure S9. HSQC spectrum of **1** in methanol-$d_4$ at 500 MHz.

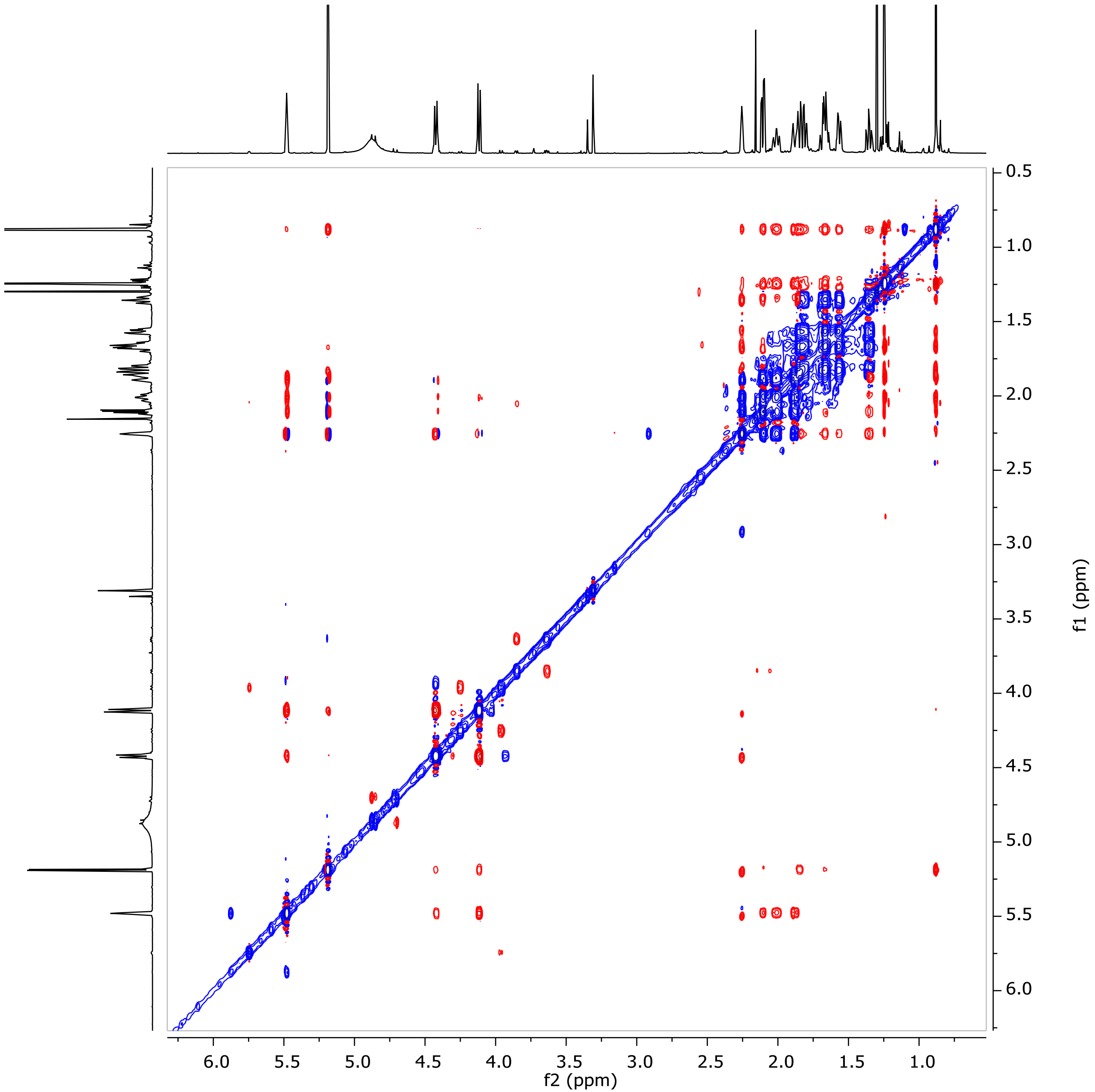


Figure S10. ROESY spectrum of **1** in methanol-$d_4$ at 700 MHz.

# Generic Display Report

**Analysis Info**

| | | | |
|---|---|---|---|
| Analysis Name | \\Neon\MWISCOM\PEOPLE\sel22_Sherif Elsayed\Abundisporus\Amazon\A. violaceus AmaZon\Abund B2F9_GB1_01_37153.d | Acquisition Date | 23.02.2022 08:59:08 |
| Method | | Operator | esu |
| Sample Name | Abund B2F9 | Instrument | amaZon speed |
| Comment | | | |

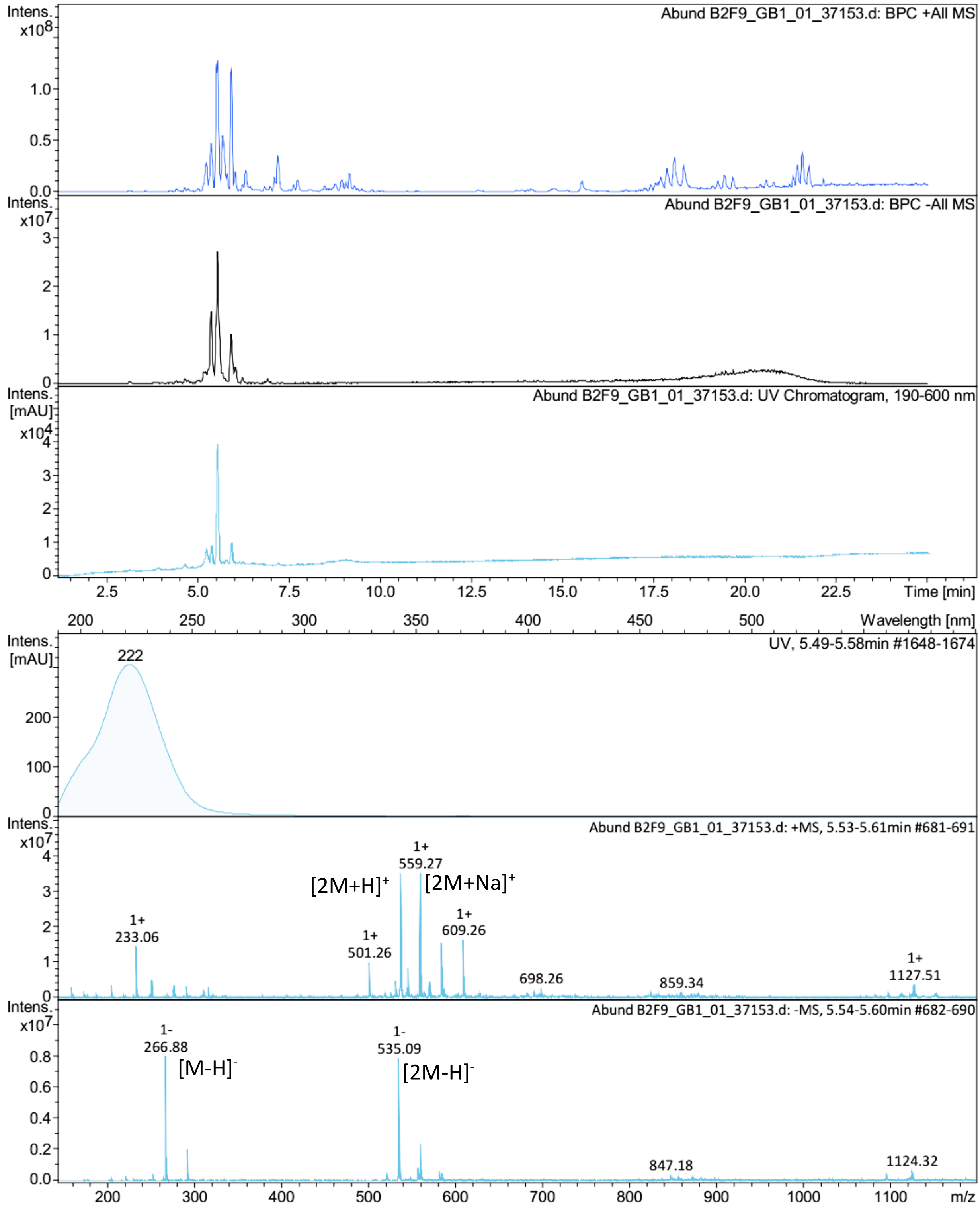




Figure S11. LRESIMS of **2**.

# Generic Display Report

**Analysis Info**

| | | | |
|---|---|---|---|
| Analysis Name | \\Neon\MWISCOM\PEOPLE\sel22_Sherif Elsayed\Abundisporus\Maxis\A. violaceus MaXis\Abund B2F9_P1-B-3_01_9620.d | Acquisition Date | 28.02.2022 14:22:38 |
| Method | [illegible]screening_ms_100_2500_line.m | Operator | ate06 |
| Sample Name | Abund B2F9 | Instrument | maXis |
| Comment | Screening01<br>Waters Acquity UPLC BEH C_18 1,7um 2.1x50mm | | |

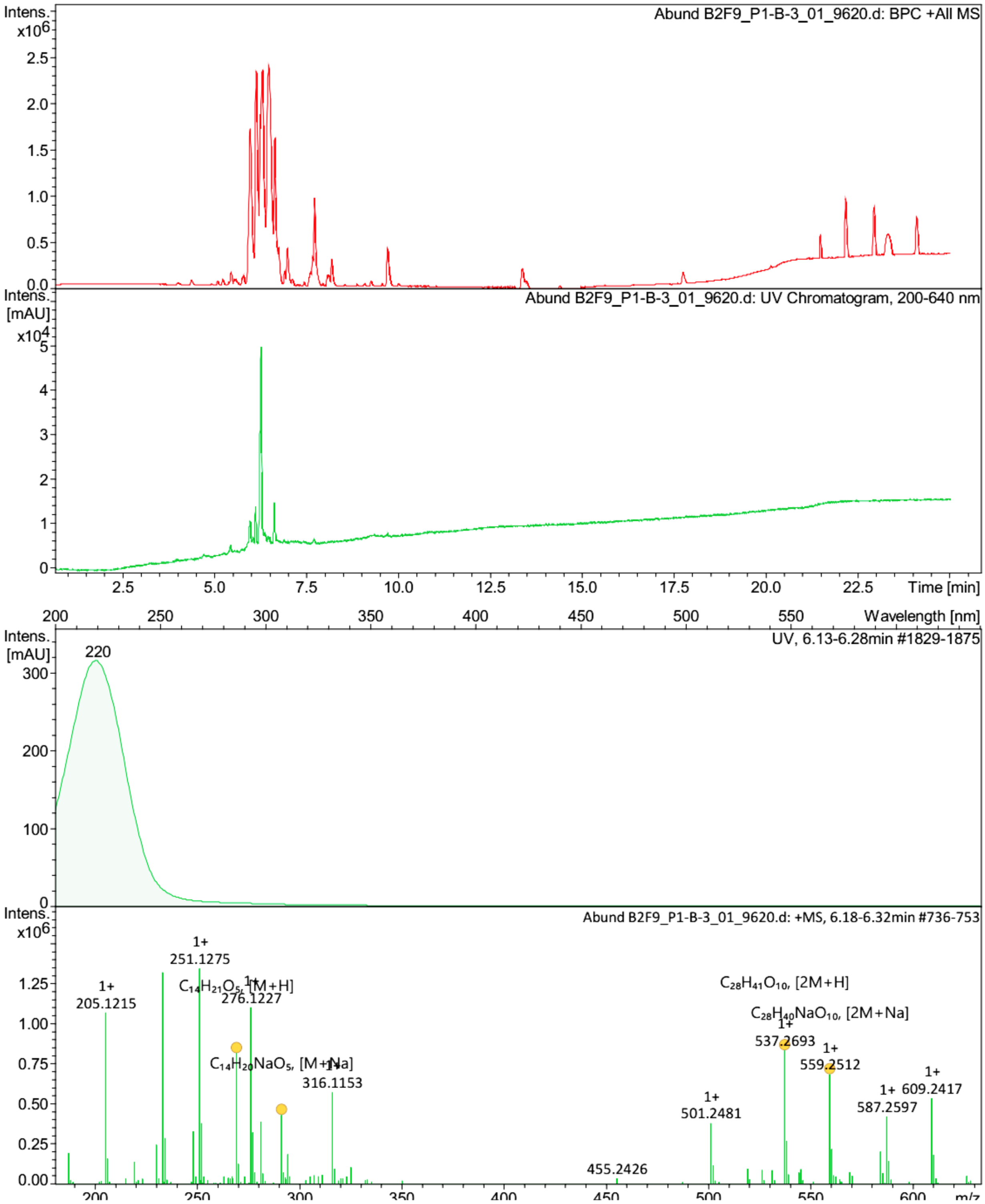




Figure S12. HRESIMS of **2**.

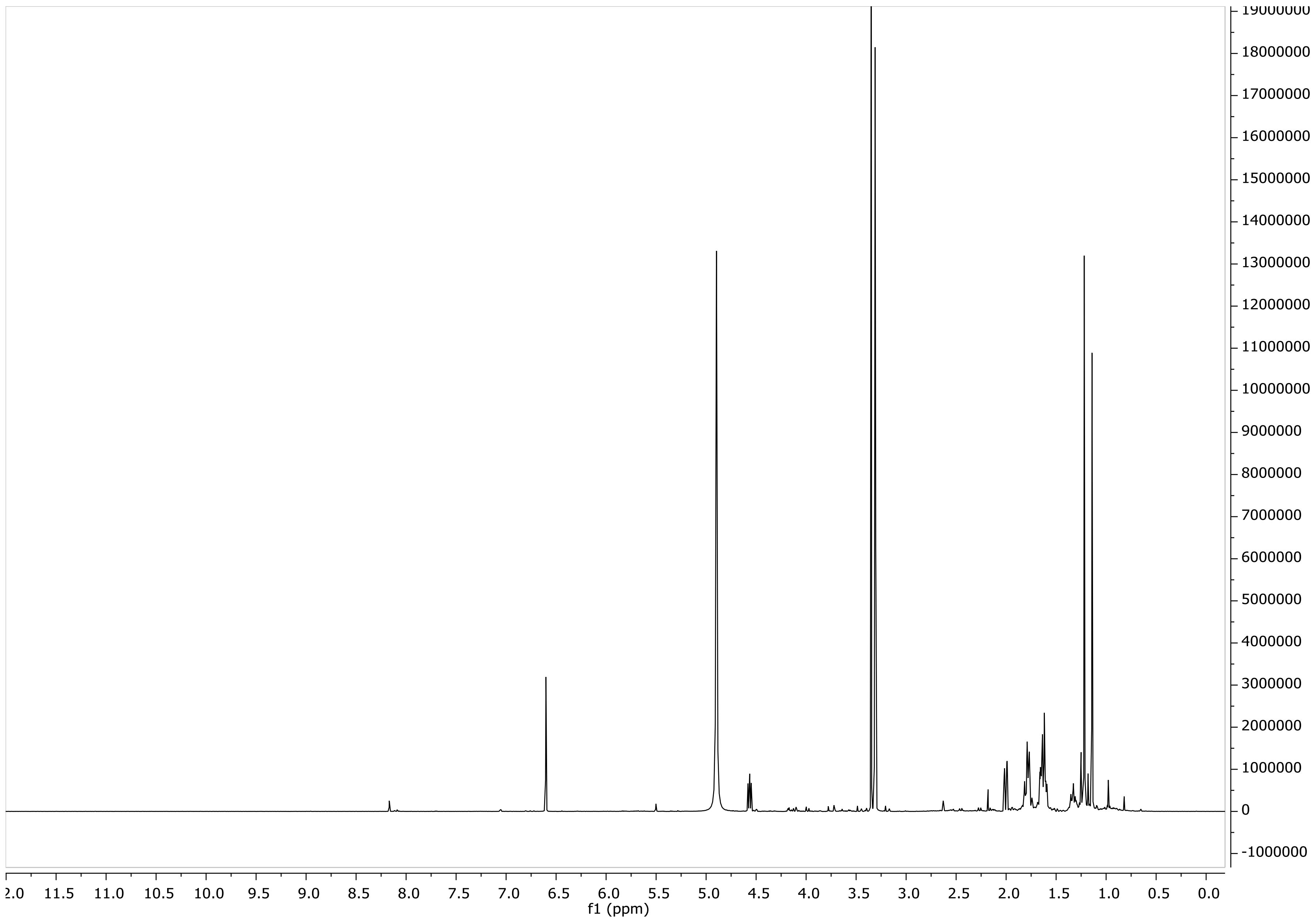


Figure S13. $^{1}$H NMR spectrum of **2** in methanol-$d_4$ at 500 MHz.

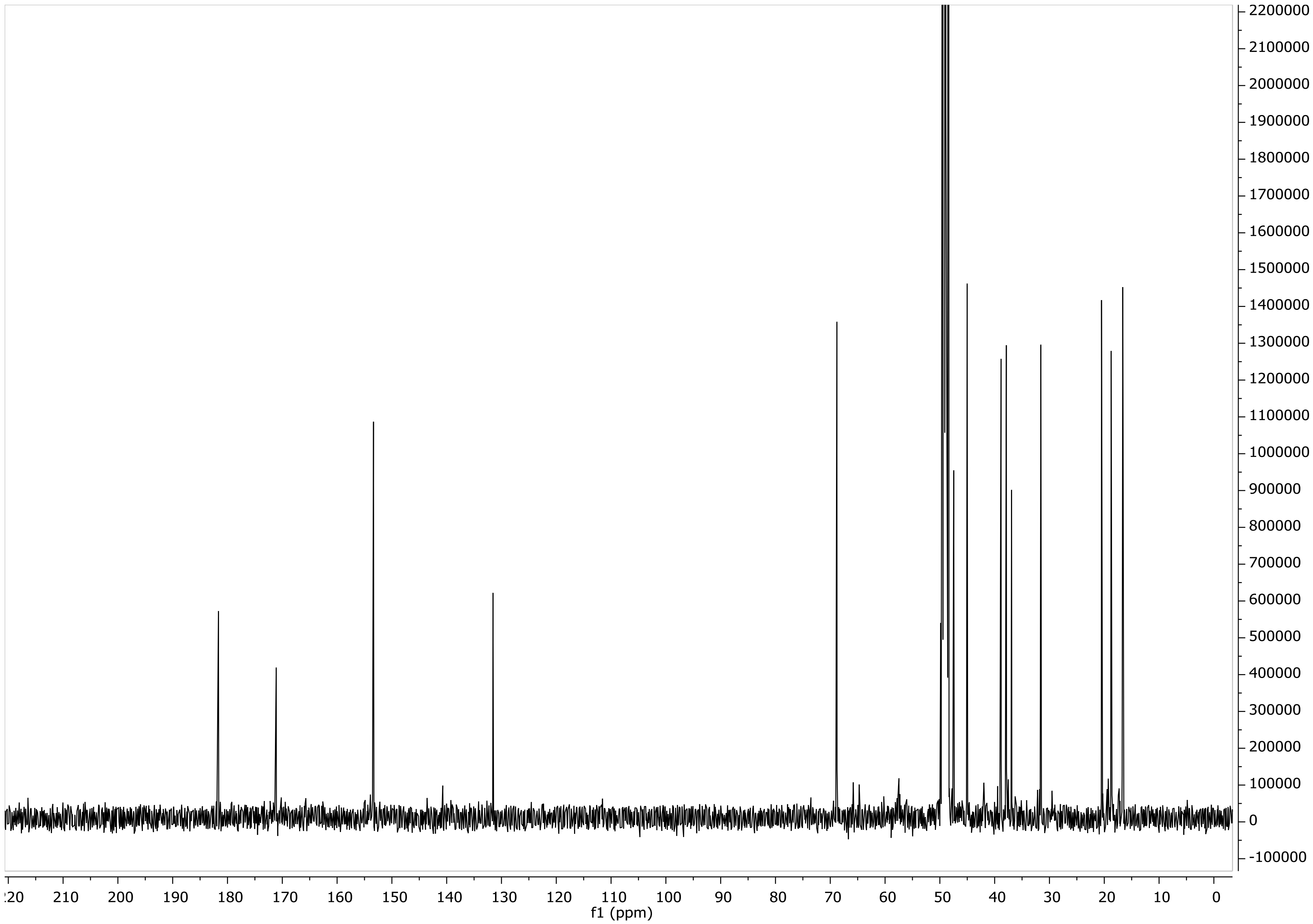


Figure S14. $^{13}$C NMR spectrum of **2** in methanol-$d_4$ at 125 MHz.

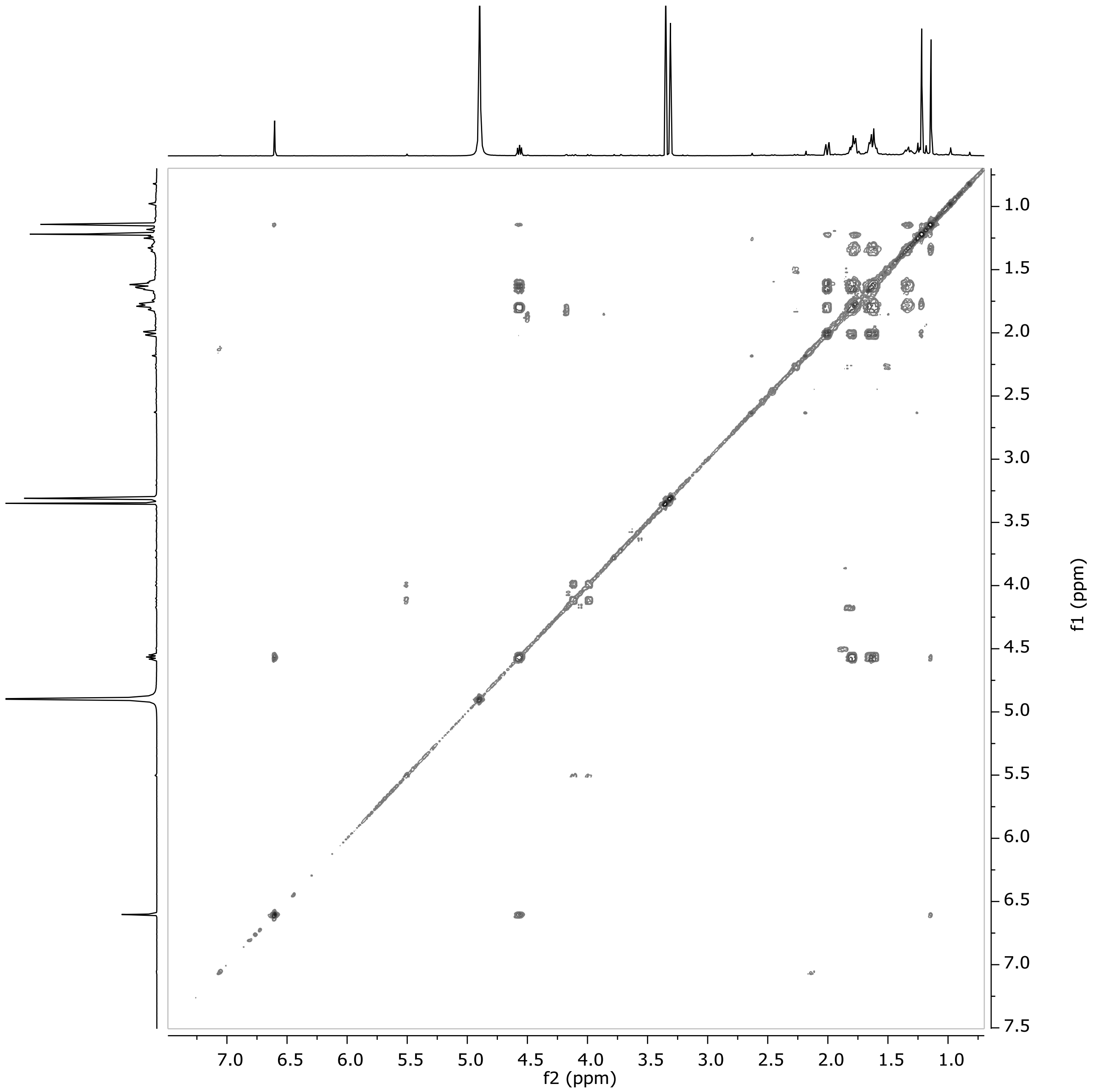


Figure S15. $^{1}$H-$^{1}$H COSY spectrum of **2** in methanol-$d_4$ at 500 MHz.

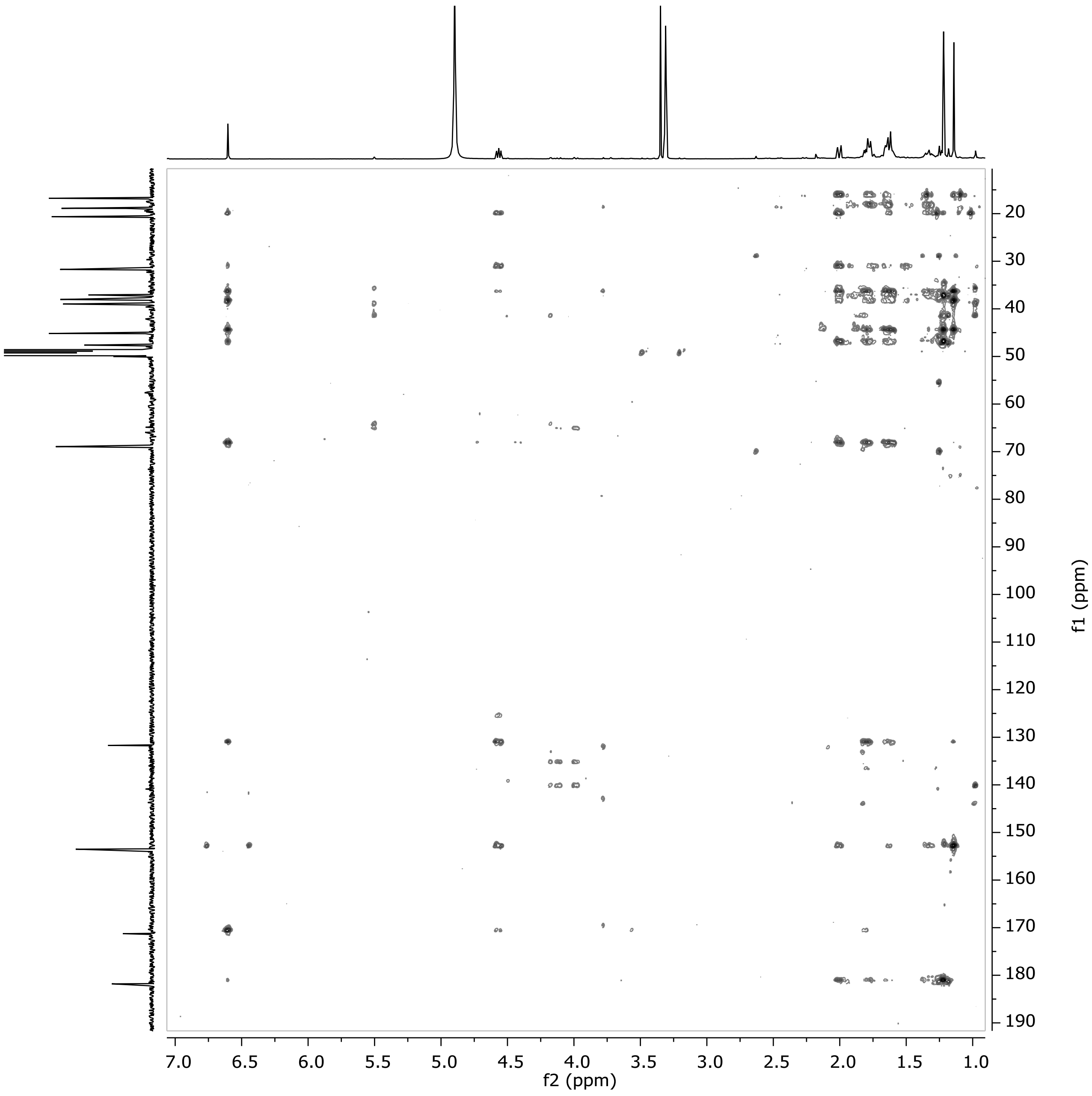


Figure S16. HMBC spectrum of **2** in methanol-$d_4$ at 500 MHz.

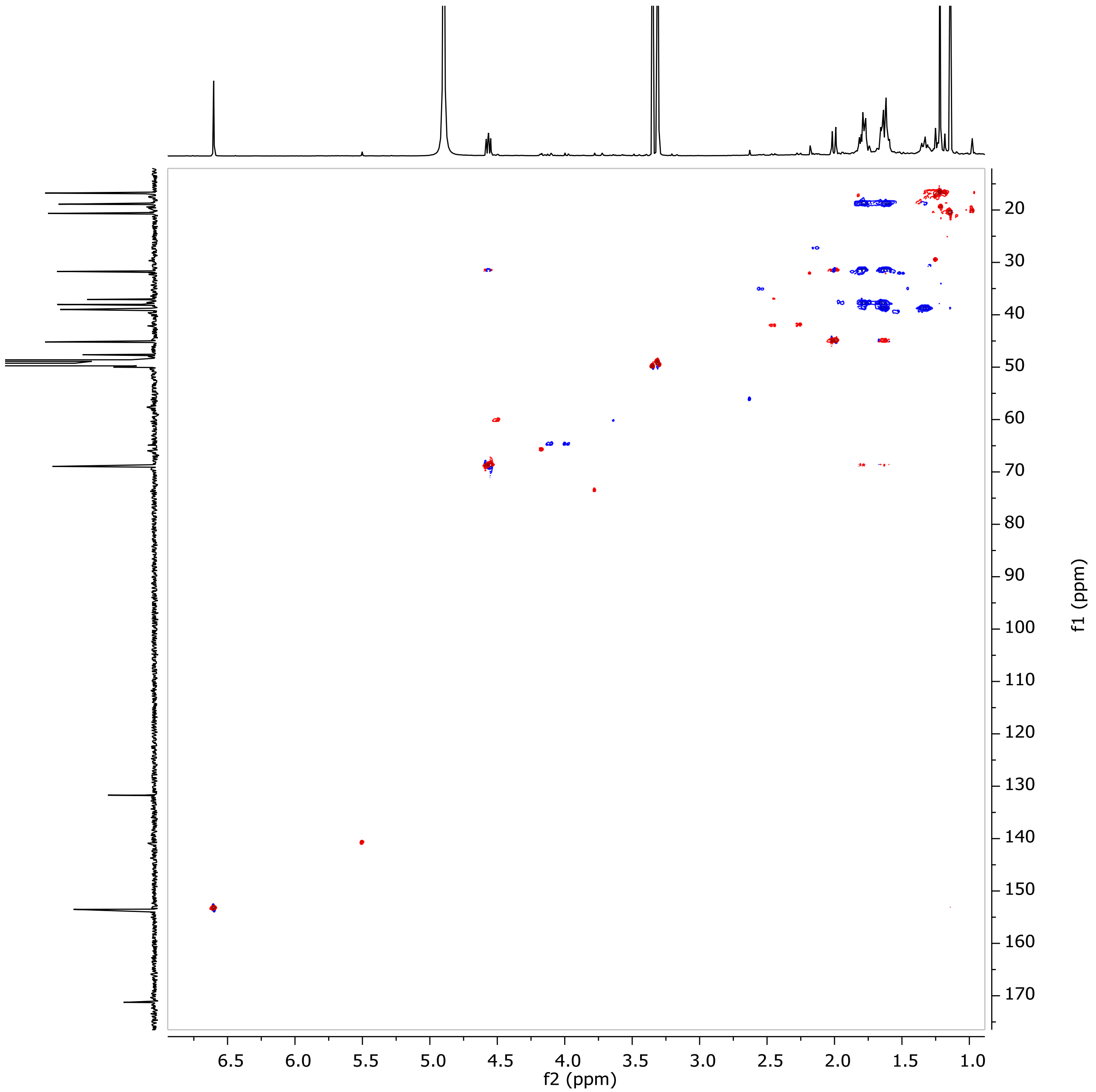


Figure S17. HSQC spectrum of **2** in methanol-$d_4$ at 500 MHz.

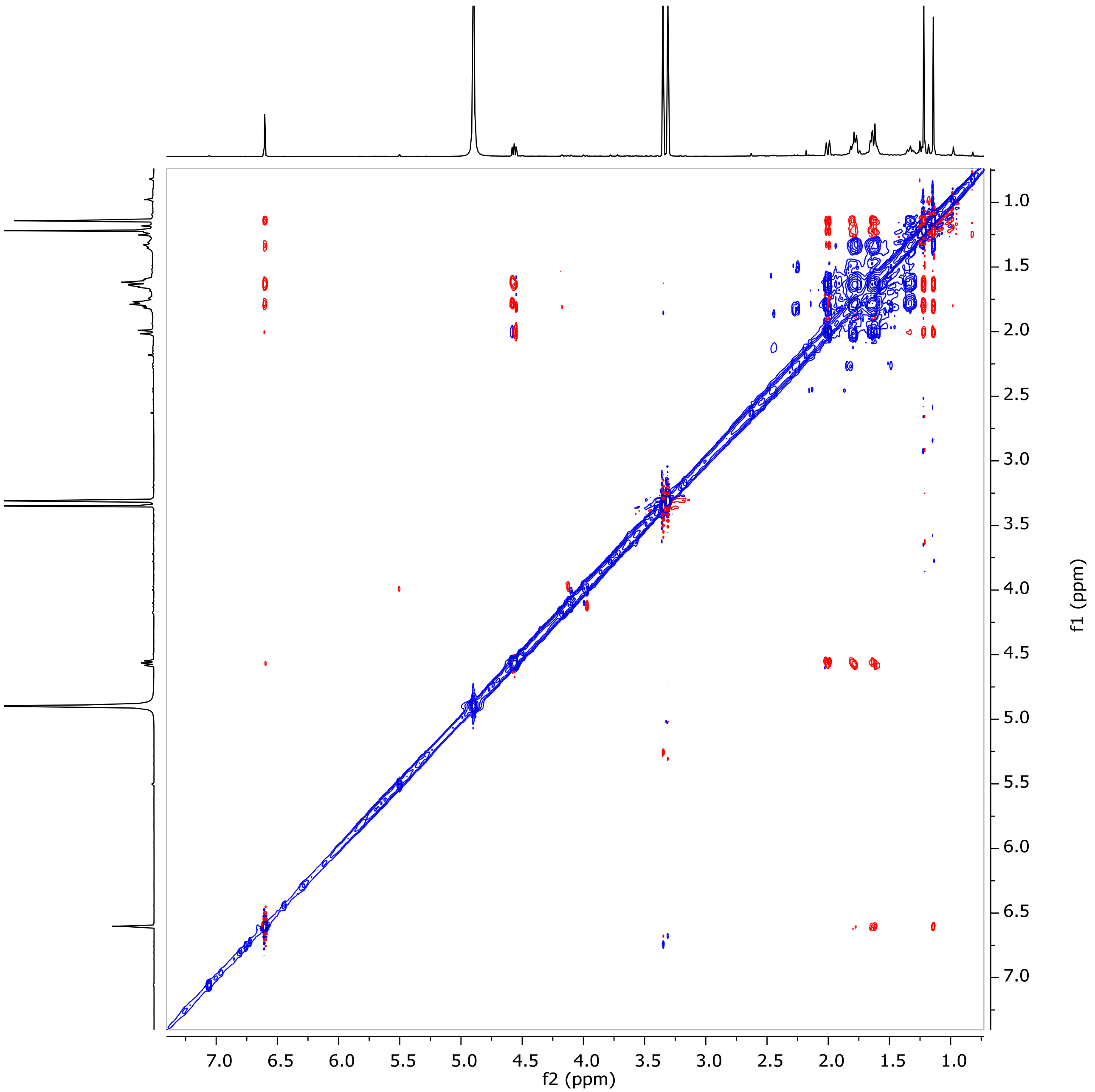


Figure S18. ROESY spectrum of **2** in methanol-$d_4$ at 500 MHz.

# Generic Display Report

| **Analysis Info** | | Acquisition Date | 02.03.2022 21:28:42 |
|---|---|---|---|
| Analysis Name | \\Neon\MWISCOM\PEOPLE\sel22_Sherif Elsayed\Abundisporus\Amazon\A. violaceus AmaZon\Abund | | |
| Method | [illegible]_10305.m | Operator | lab |
| Sample Name | Abund B1F5F3 | Instrument | amaZon speed |
| Comment | | | |

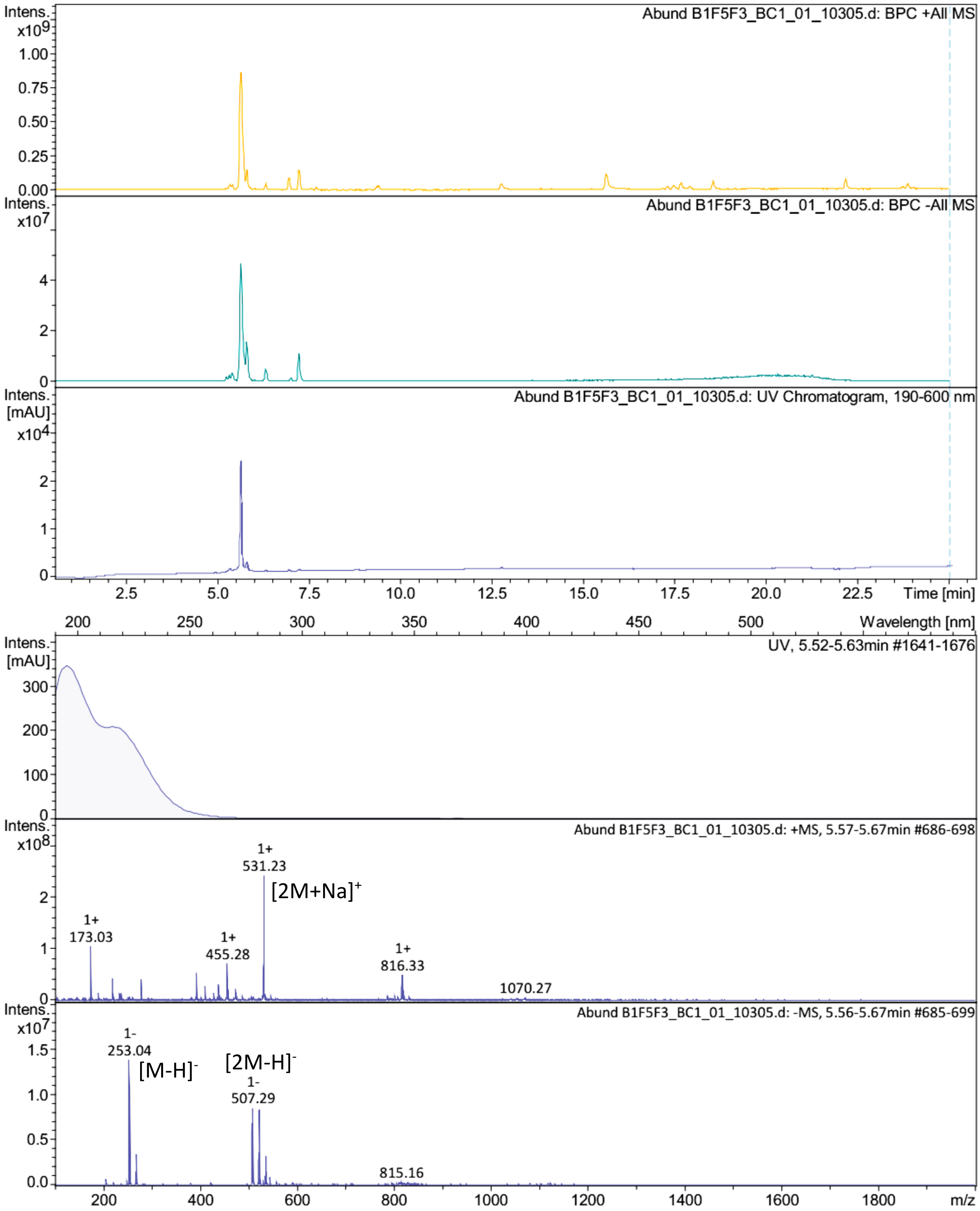


Bruker Compass DataAnalysis 4.4 printed: 11.04.2023 18:17:34 by: mk-mwisnmr Page 1 of 1

Figure S19. LRESIMS of **3**.

# Generic Display Report

**Analysis Info**

| | | | |
|---|---|---|---|
| | | Acquisition Date | 09.03.2022 12:12:05 |
| Analysis Name | \\Neon\MWISCOM\PEOPLE\sel22_Sherif Elsayed\Abundisporus\Maxis\A. violaceus MaXis\Abund B1F5F3_P1-B-2_01_9678.d | | |
| Method | pos_saure_1800_screening_ms_100_2500_line.m | Operator | ate06 |
| Sample Name | Abund B1F5F3 | Instrument | maXis |
| Comment | Screening01<br>Waters Acquity UPLC BEH C_18 1,7um 2.1x50mm | | |

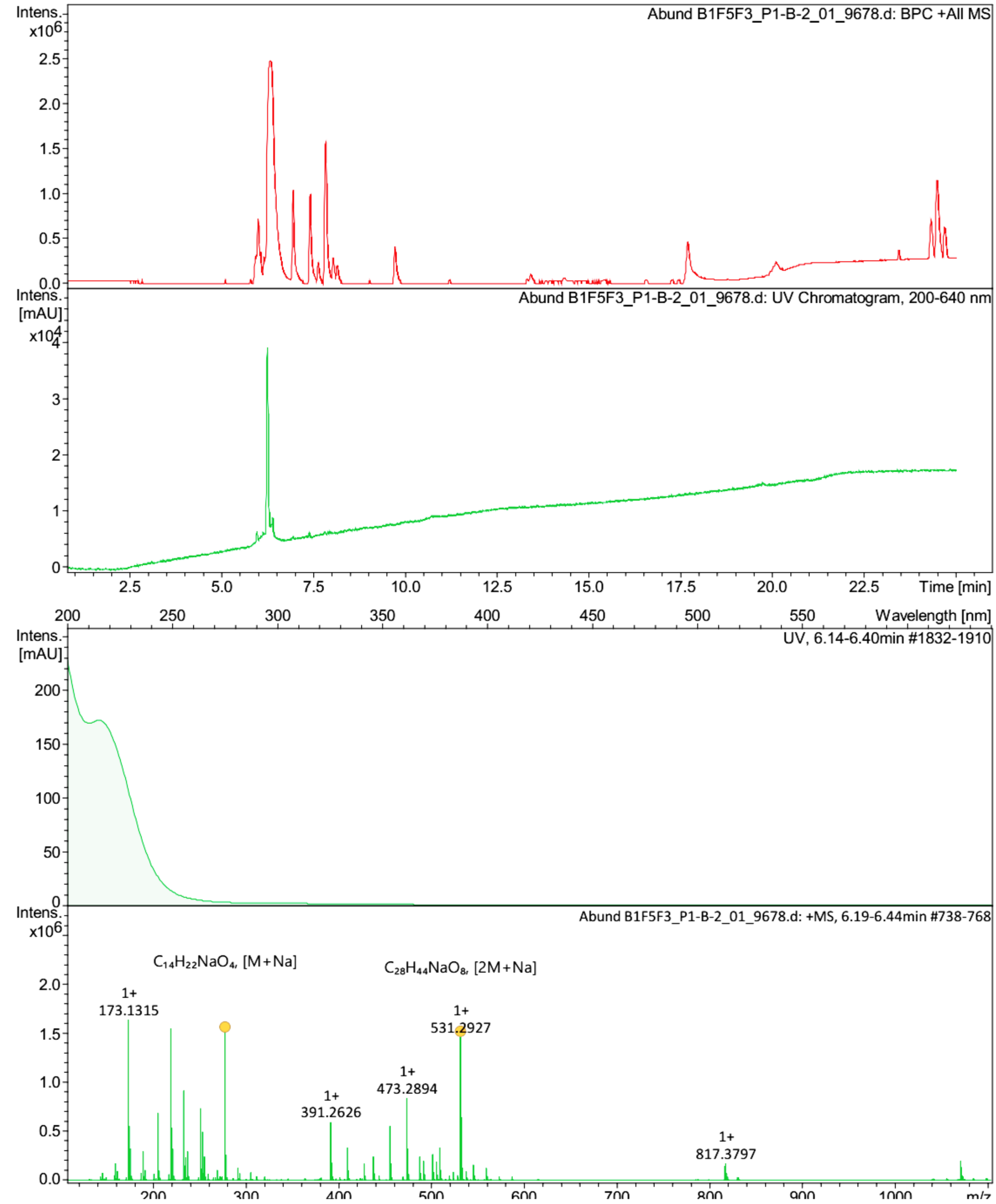


Bruker Compass DataAnalysis 4.4 printed: 11.04.2023 18:38:12 by: mk-mwisnmr Page 1 of 1

Figure S20. HRESIMS of **3**.

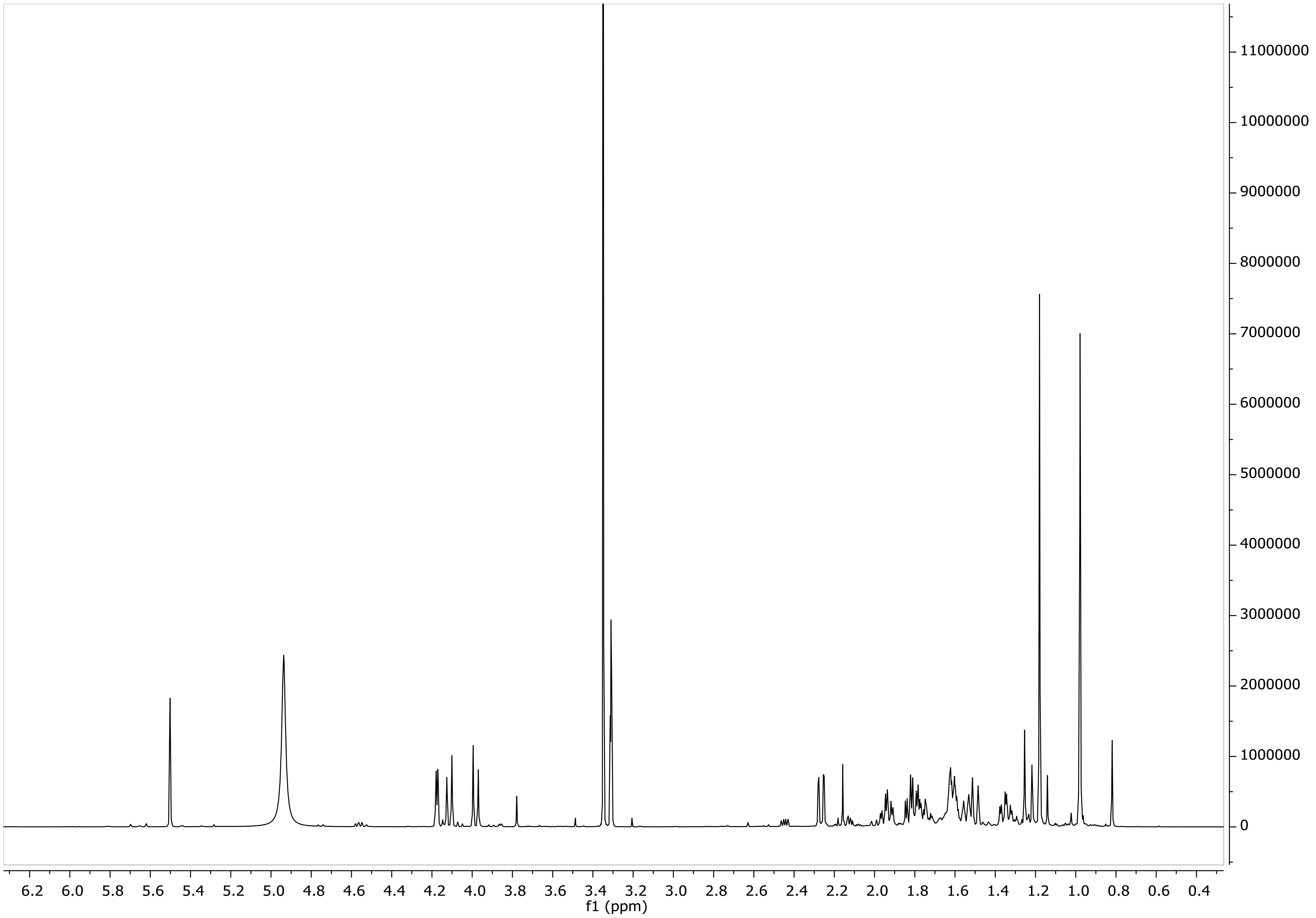


Figure S21. $^{1}H$ NMR spectrum of **3** in methanol-*d₄* at 500 MHz.

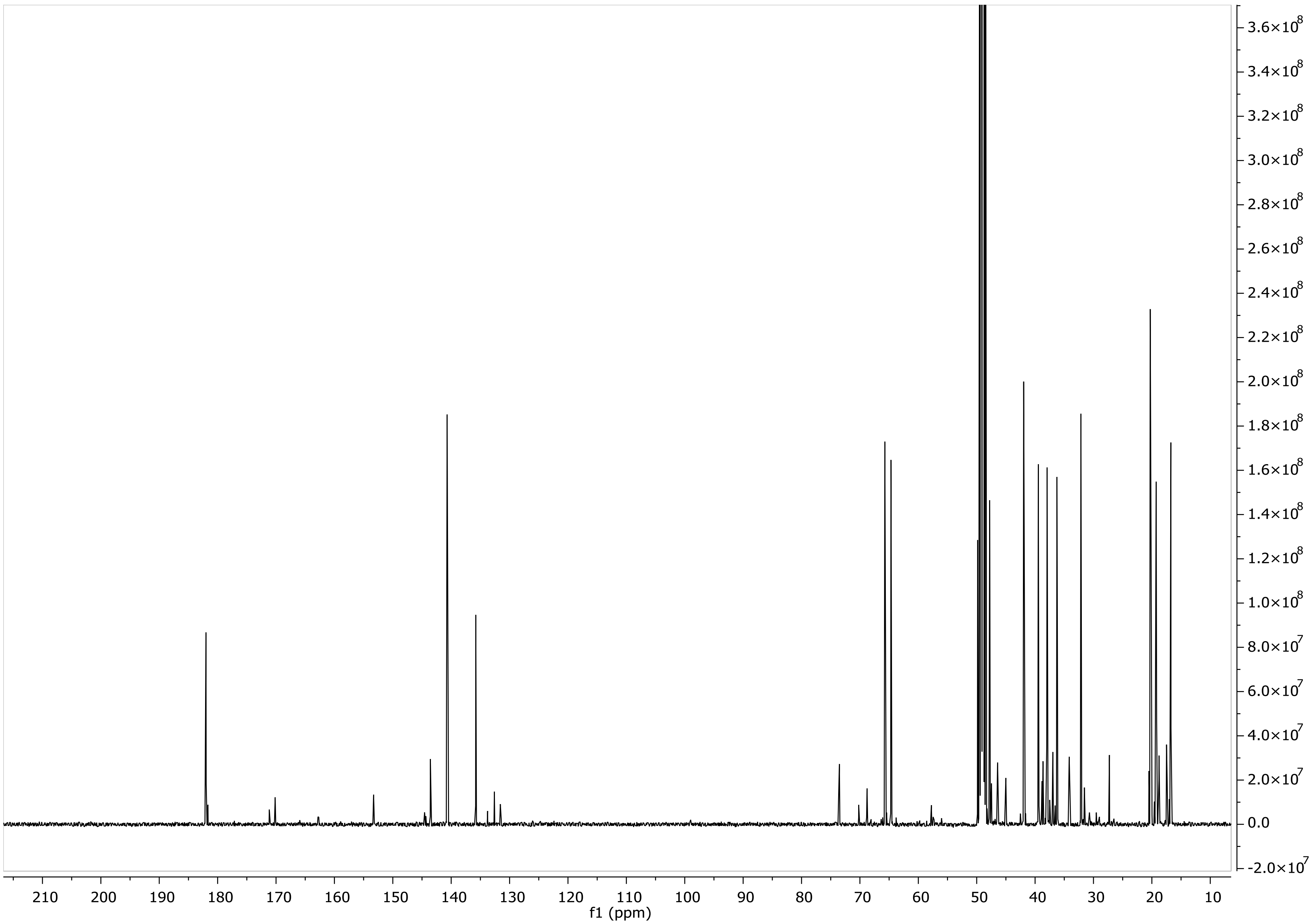


Figure S22. $^{13}$C NMR spectrum of **3** in methanol-$d_4$ at 125 MHz.

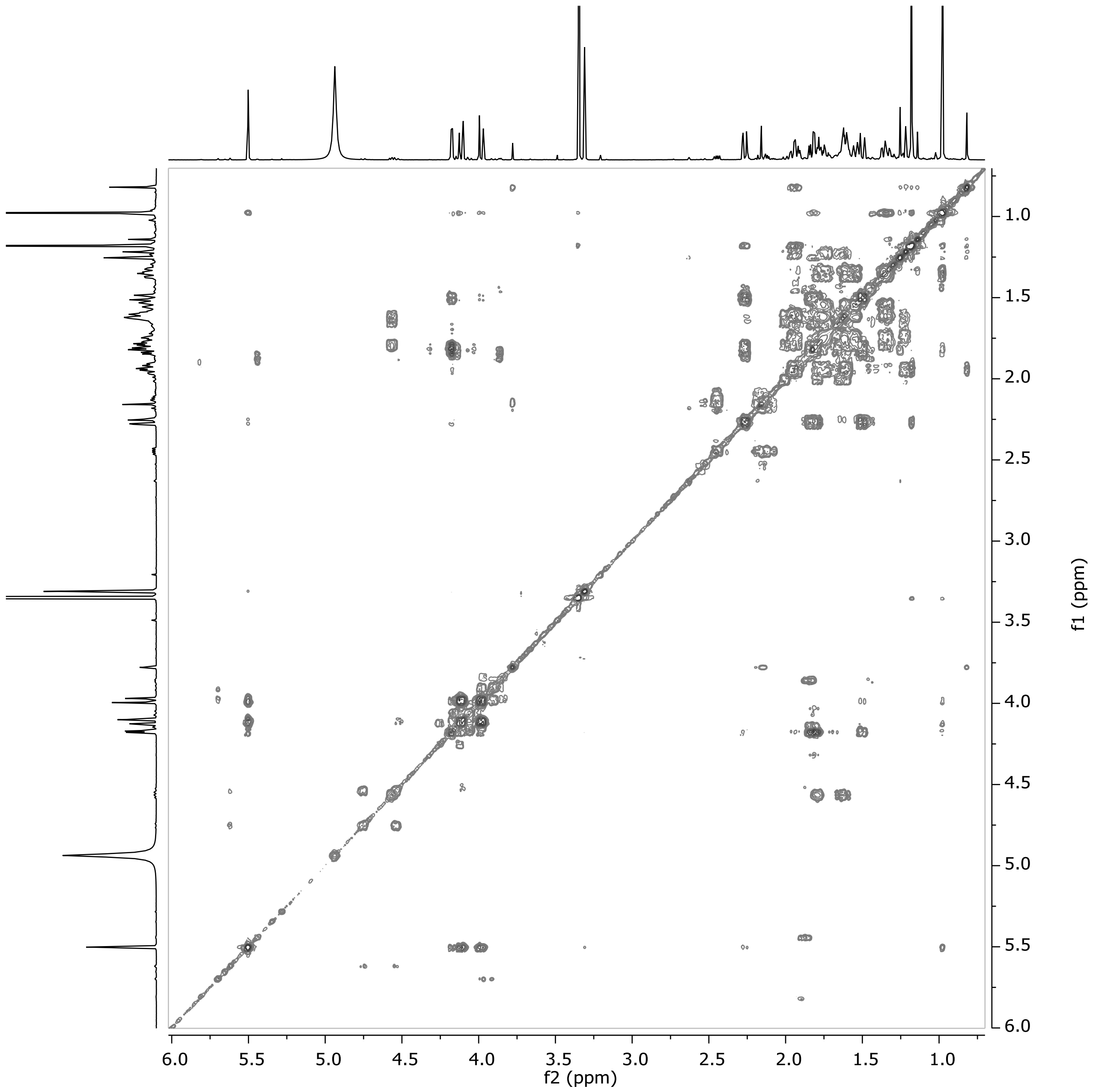


Figure S23. $^1H$-$^1H$ COSY spectrum of **3** in methanol-$d_4$ at 500 MHz.

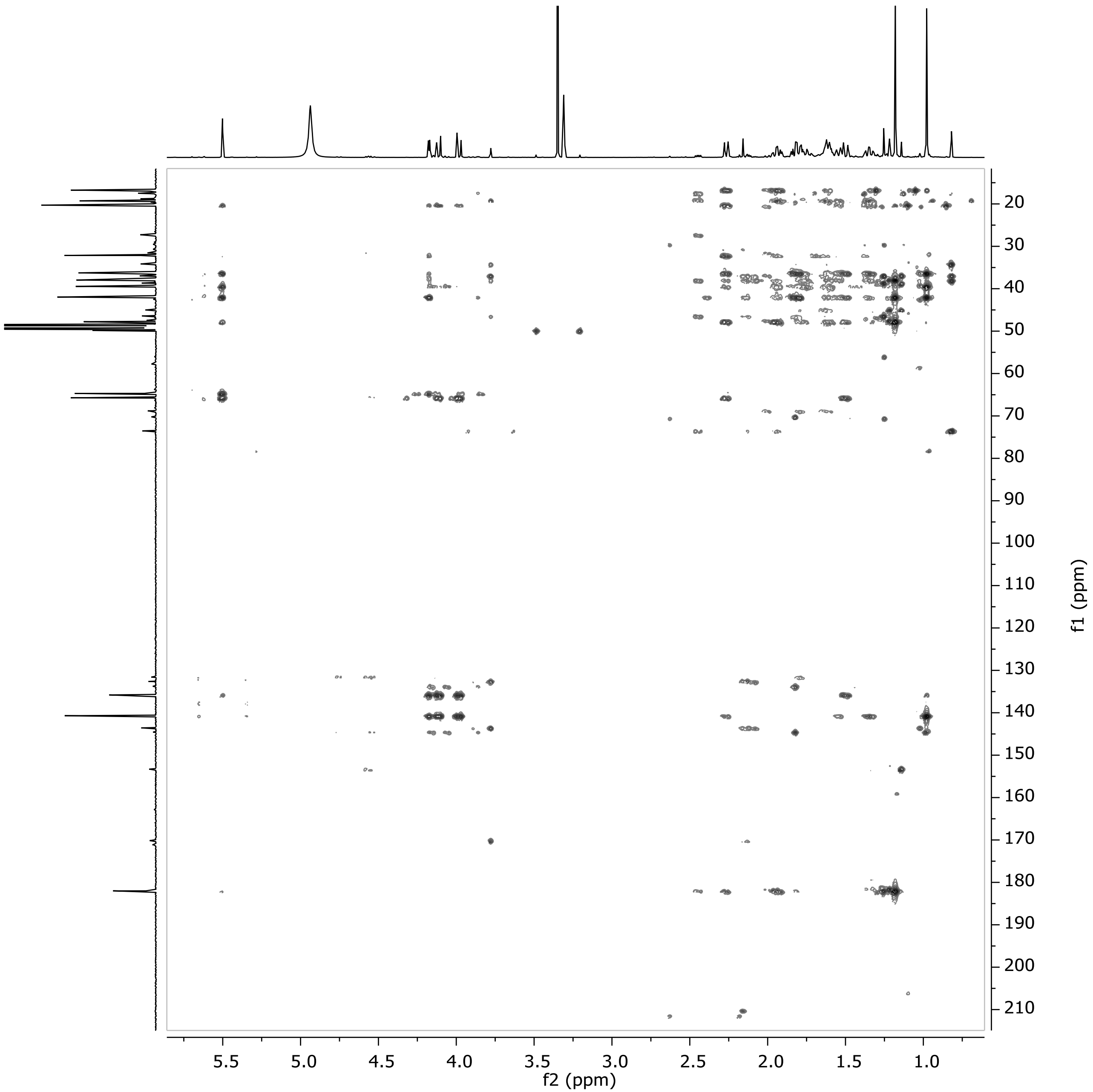


Figure S24. HMBC spectrum of **3** in methanol-$d_4$ at 500 MHz.

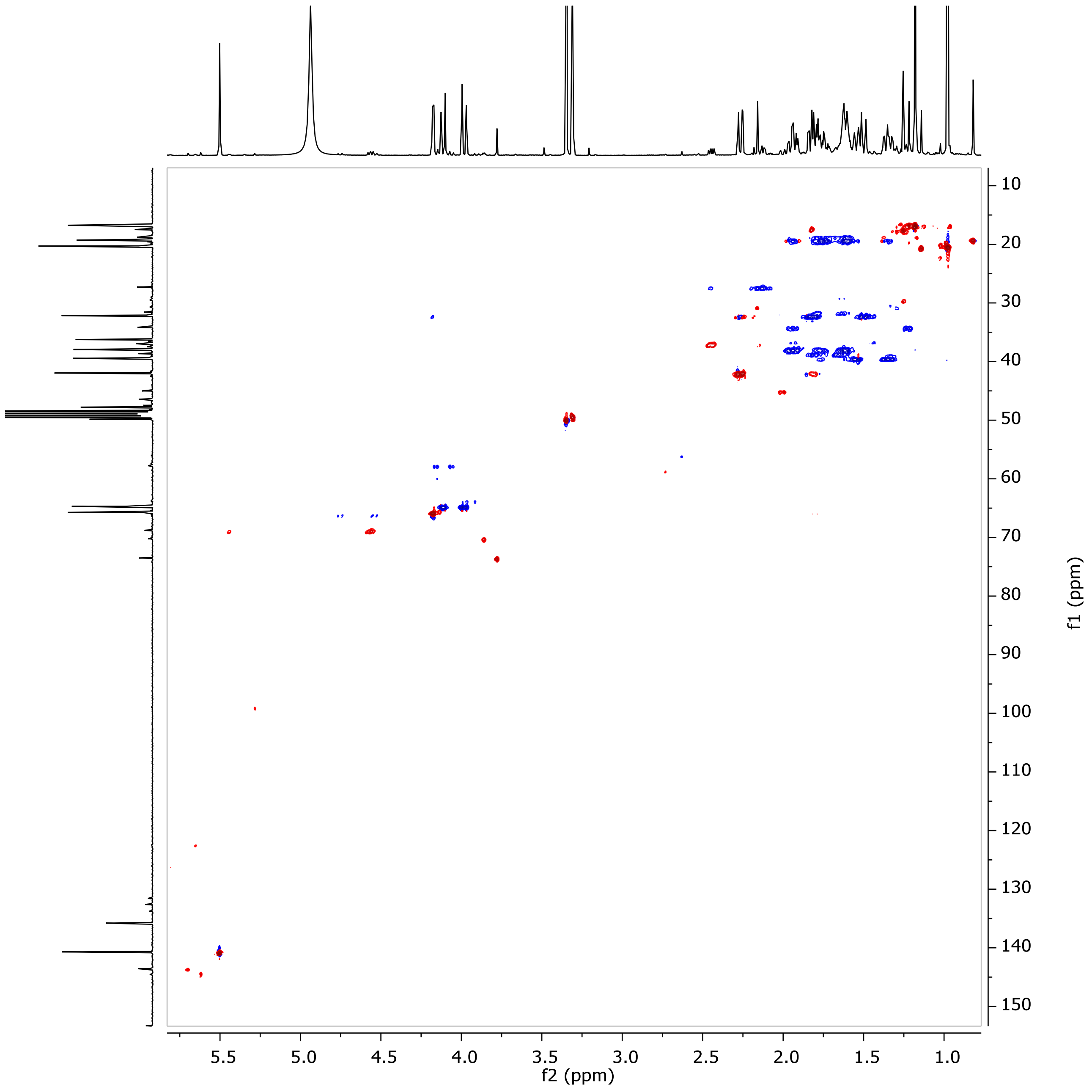


Figure S25. HSQC spectrum of **3** in methanol-$d_4$ at 500 MHz.

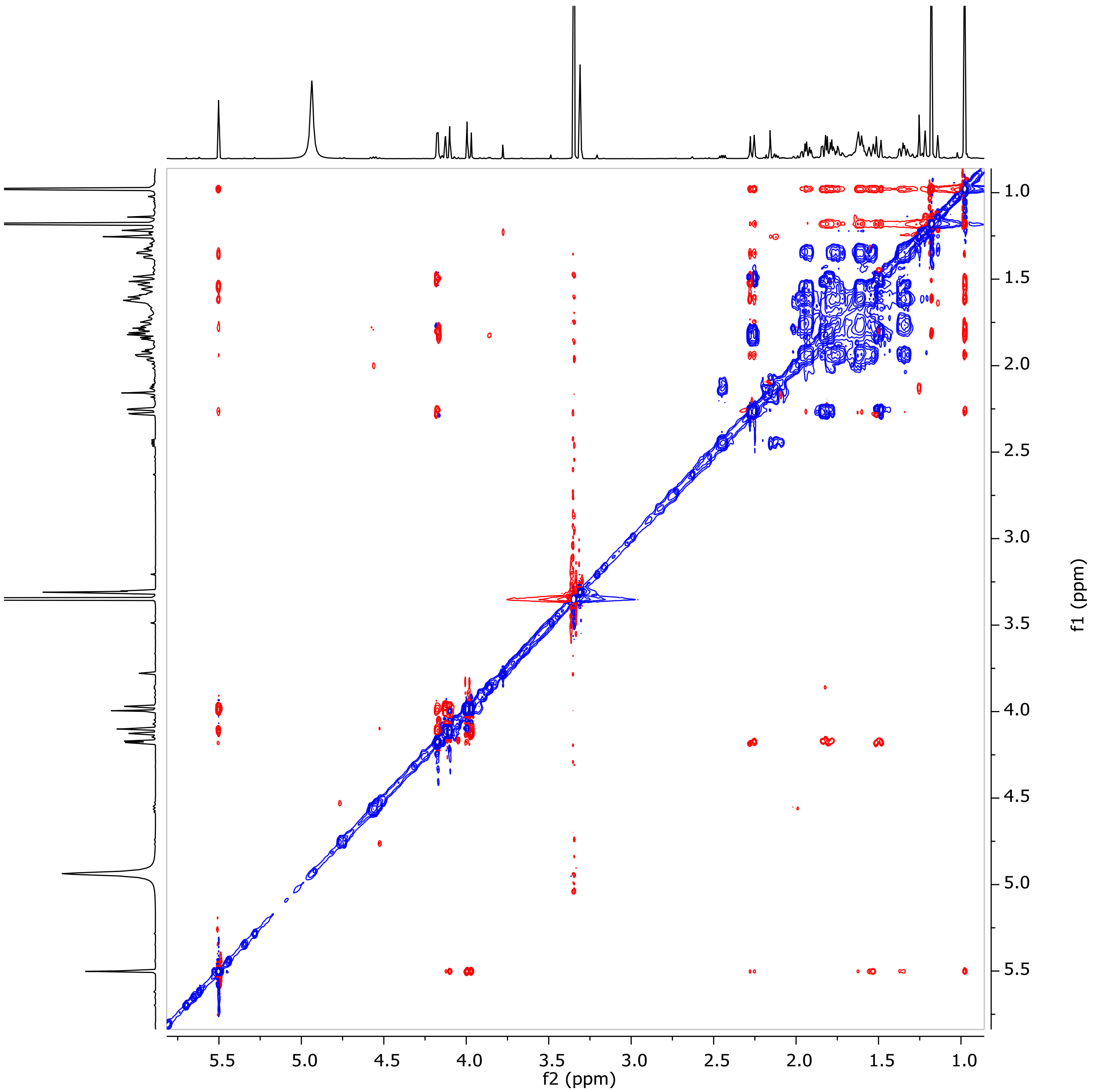


Figure S26. ROESY spectrum of **3** in methanol-$d_4$ at 500 MHz.

# Generic Display Report

**Analysis Info**

| | | | |
|---|---|---|---|
| Analysis Name | \\Neon\MWISCOM\PEOPLE\sel22_Sherif Elsayed\Abundisporus\Amazon\A. violaceus AmaZon\Abund B2F1_GA1_01_37140.d | Acquisition Date | 22.02.2022 13:36:05 |
| Method | [illegible] | Operator | esu |
| Sample Name | Abund B2F1 | Instrument | amaZon speed |
| Comment | | | |

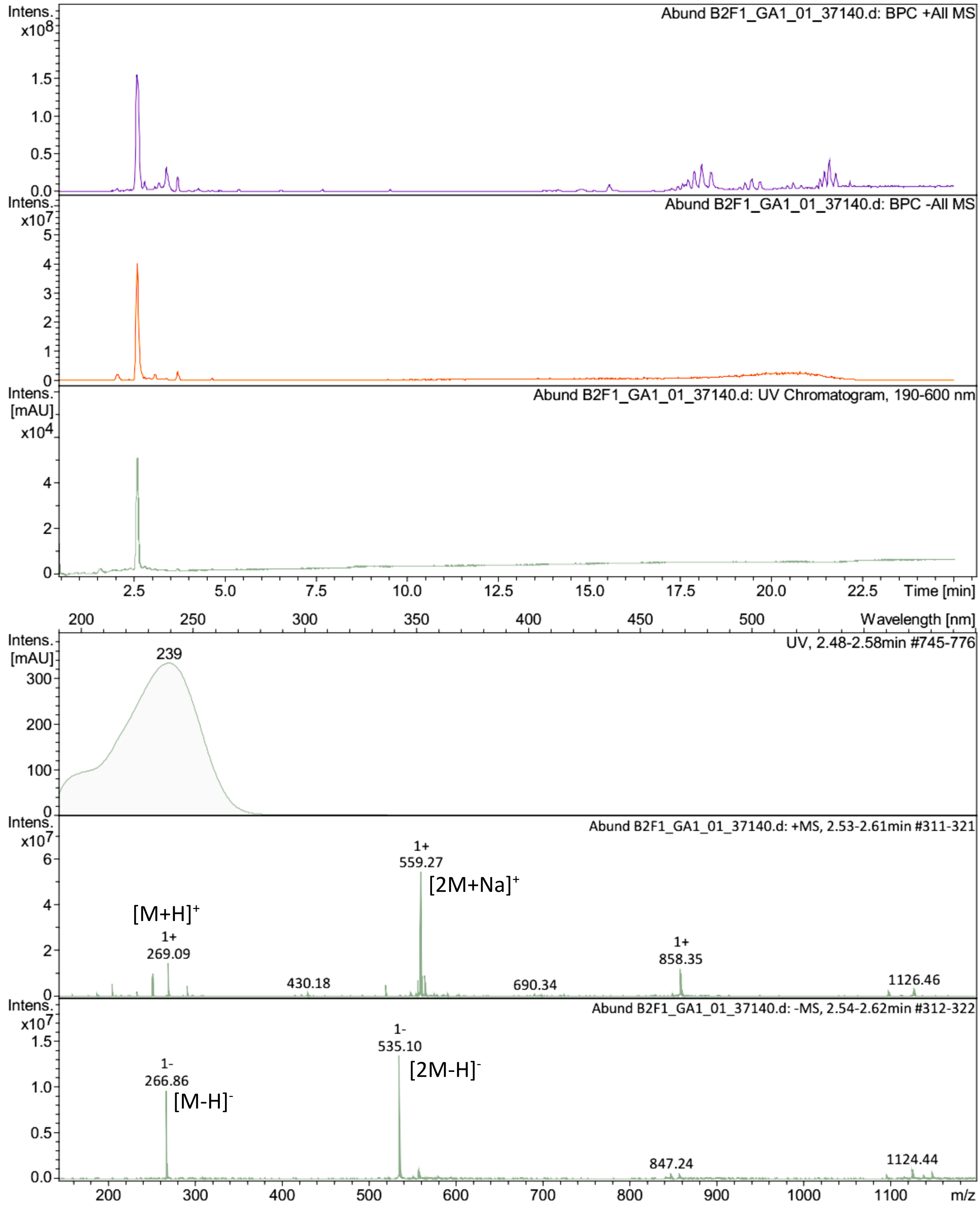


Bruker Compass DataAnalysis 4.4 printed: 11.04.2023 18:20:13 by: mk-mwisnmr Page 1 of 1

Figure S27. LRESIMS of **4**.

# Generic Display Report

| | | | |
|---|---|---|---|
| **Analysis Info** | | Acquisition Date | 28.02.2022 13:51:40 |
| Analysis Name | \\Neon\MWISCOM\PEOPLE\sel22_Sherif Elsayed\Abundisporus\Maxis\A. violaceus MaXis\Abund B2F1_P1-B-2_01_9619.d | | |
| Method | pos_[illegible]_screening_ms_100_2500_line.m | Operator | ate06 |
| Sample Name | Abund B2F1 | Instrument | maXis |
| Comment | Screening01<br>Waters Acquity UPLC BEH C_18 1,7um 2.1x50mm | | |

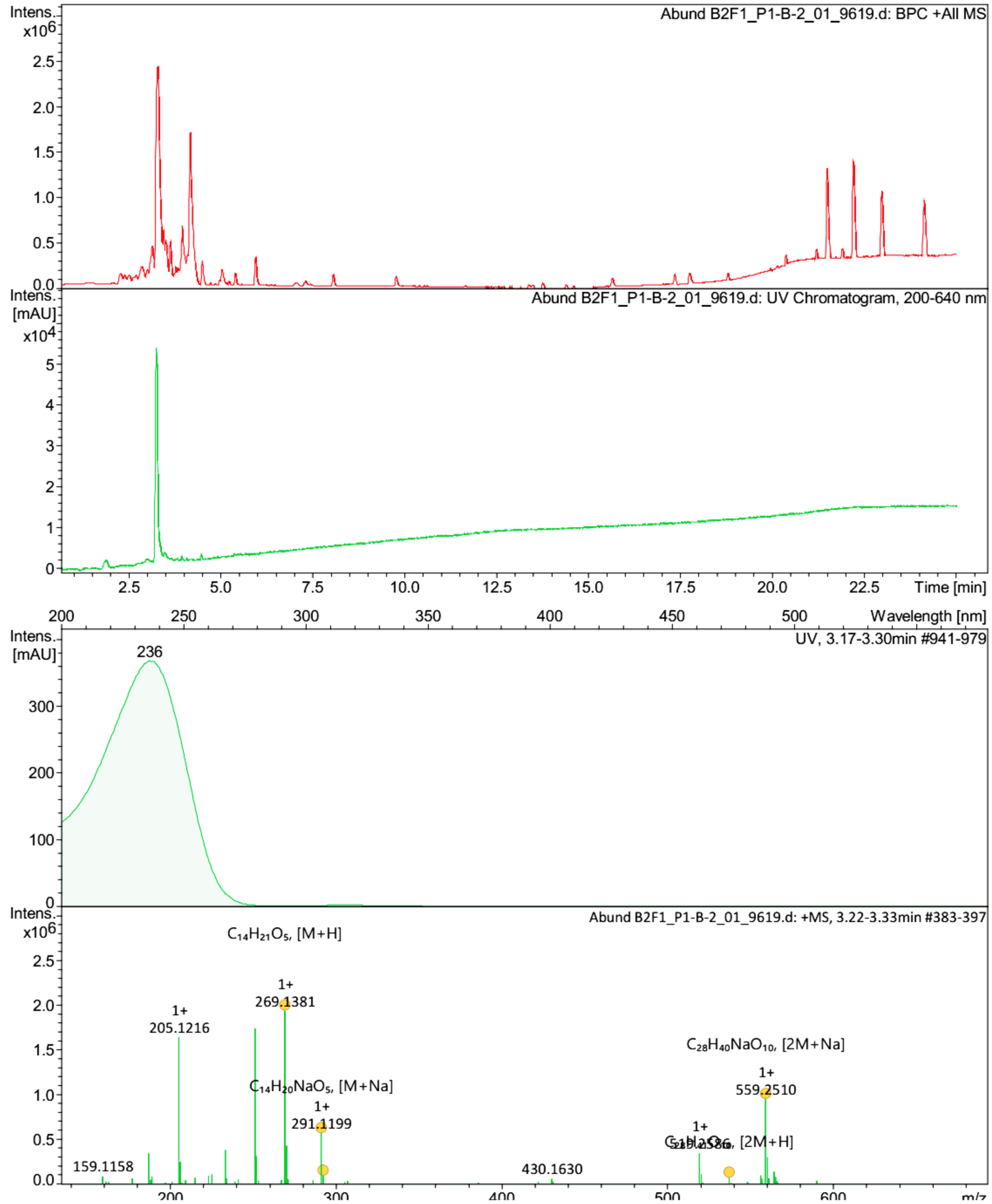




Figure S28. HRESIMS of **4**.

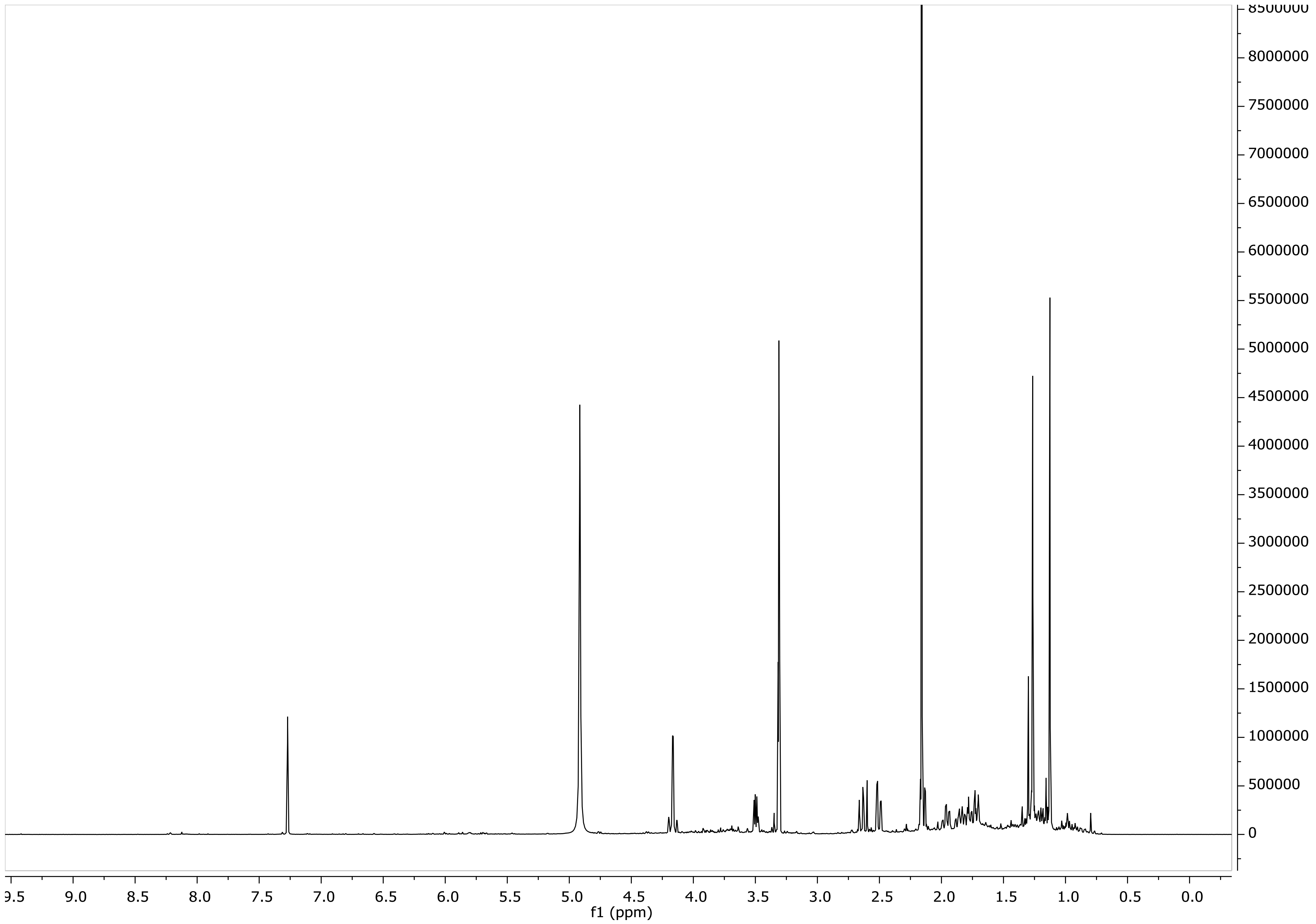


Figure S29. $^{1}H$ NMR spectrum of **4** in methanol-$d_4$ at 500 MHz.

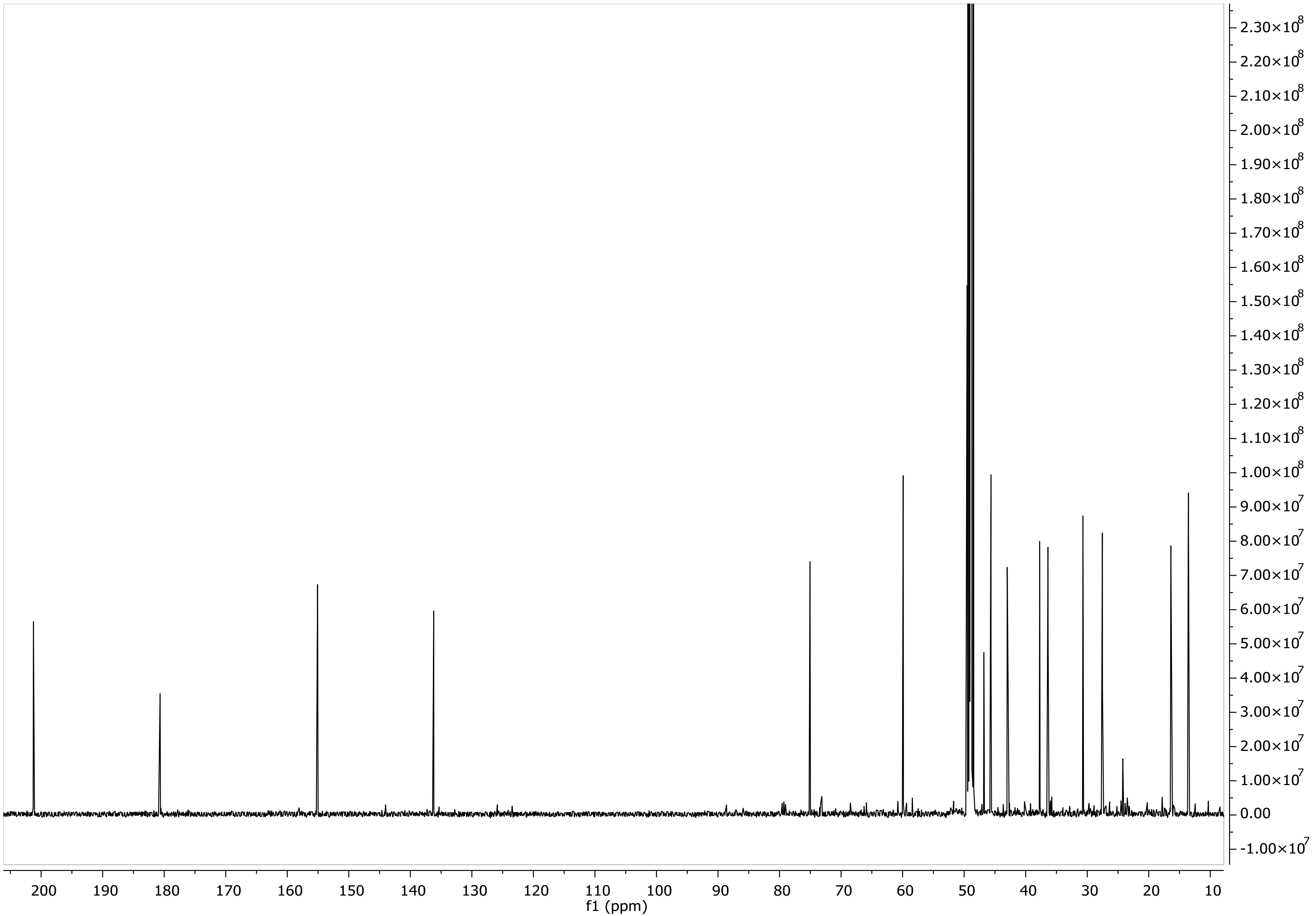


Figure S30. $^{13}$C NMR spectrum of **4** in methanol-$d_4$ at 125 MHz.

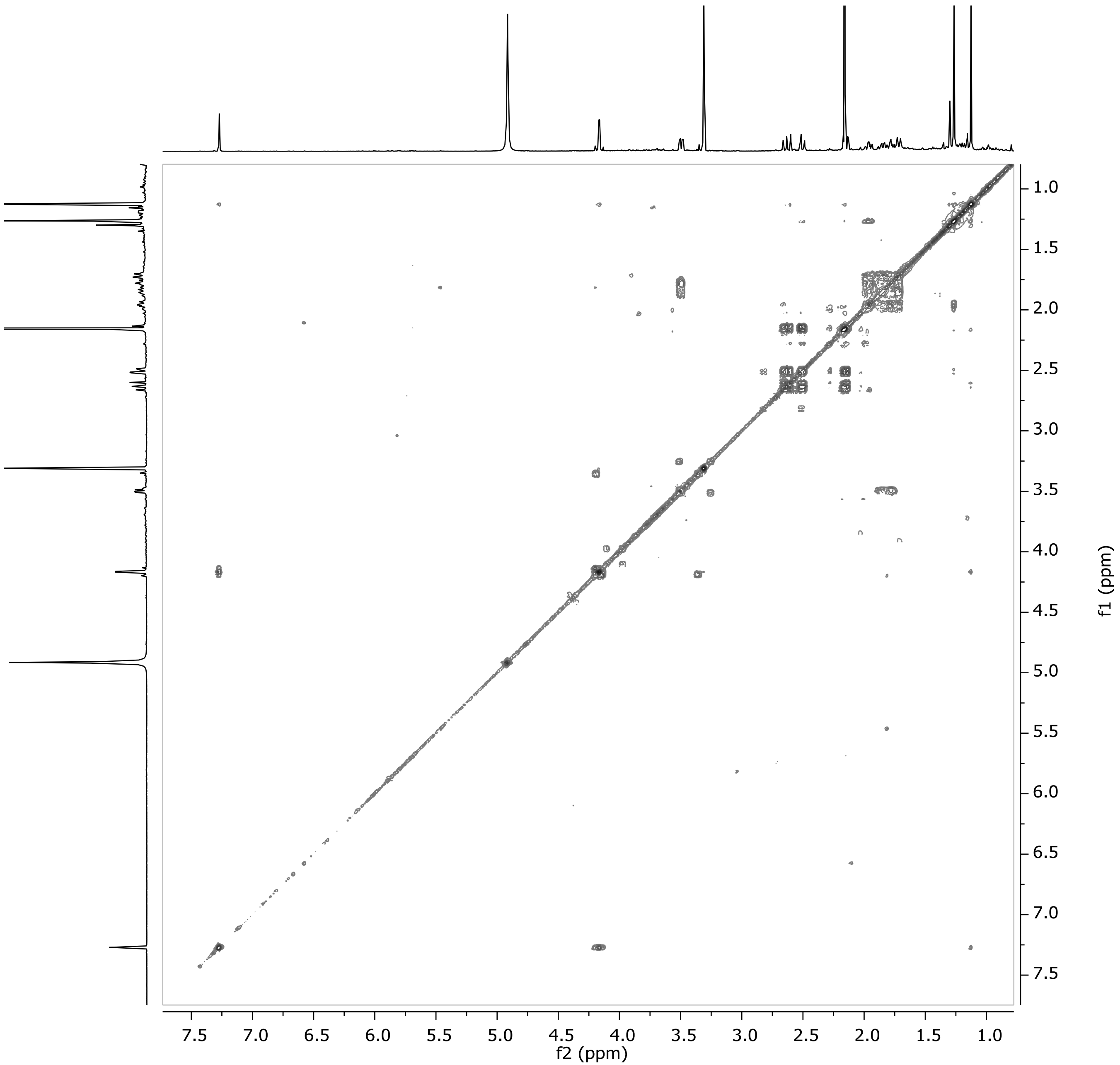


Figure S31. $^1$H-$^1$H COSY spectrum of **4** in methanol-$d_4$ at 500 MHz.

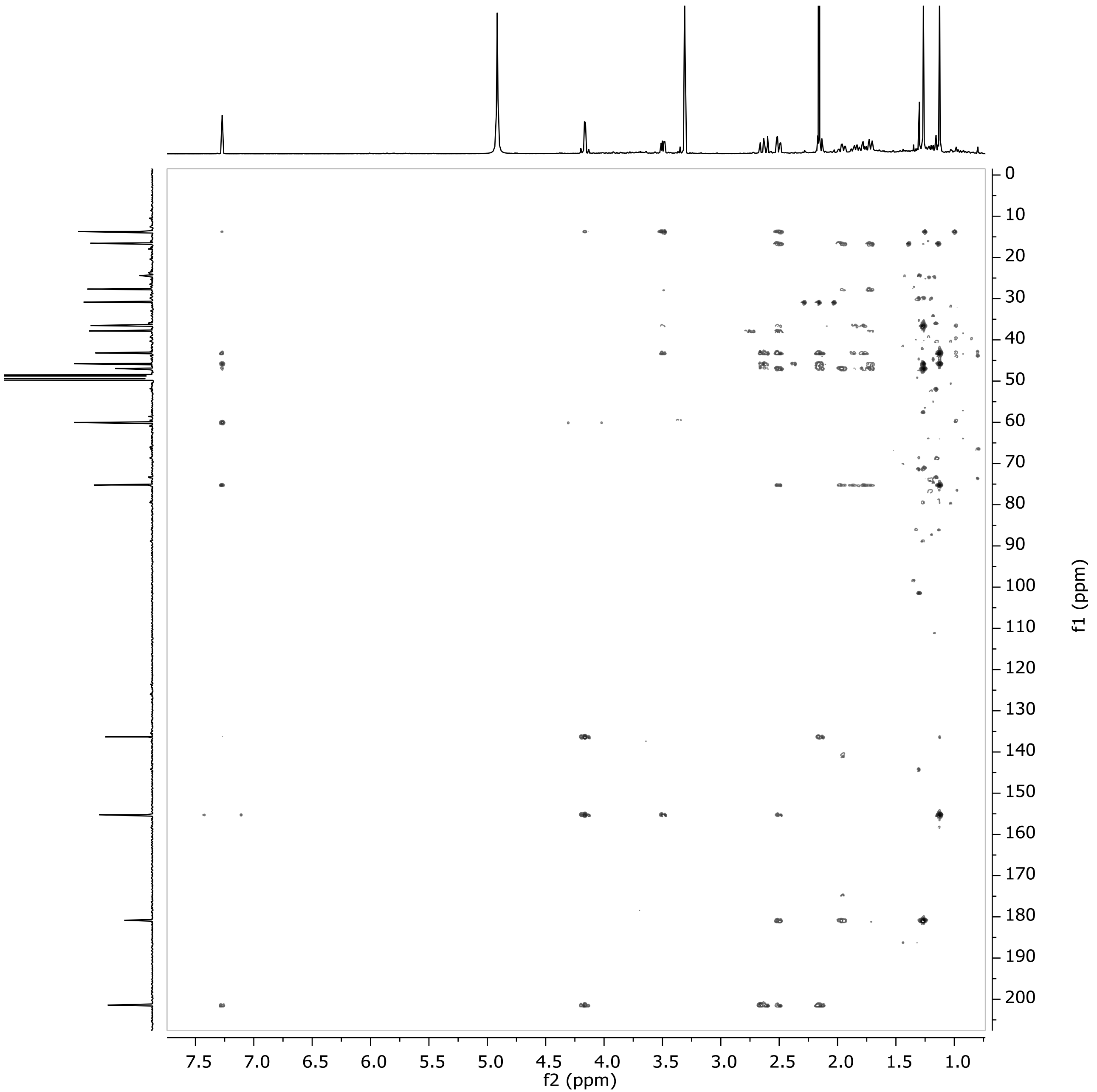


Figure S32. HMBC spectrum of **4** in methanol-$d_4$ at 500 MHz.

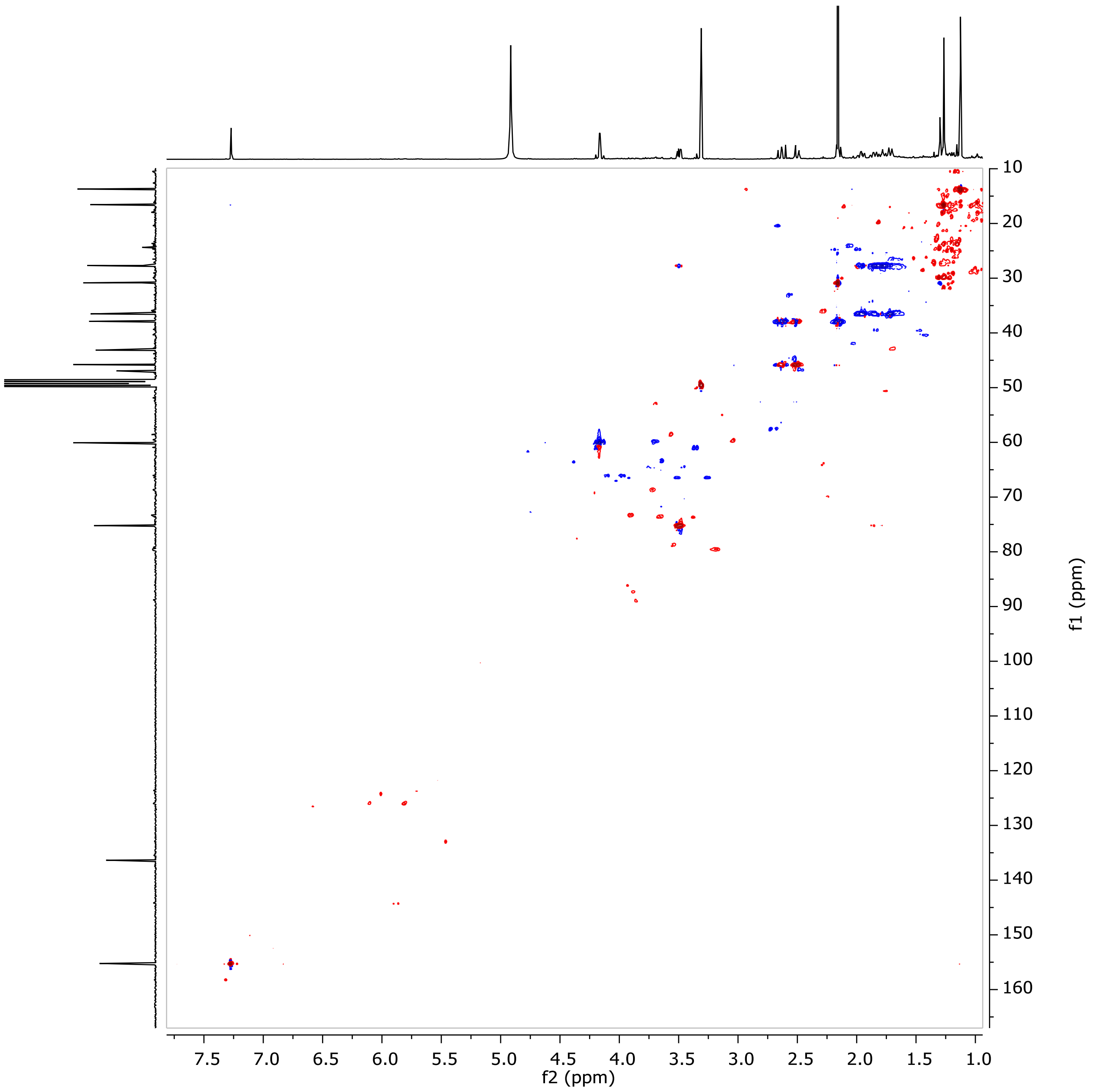


Figure S33. HSQC spectrum of **4** in methanol-$d_4$ at 500 MHz.

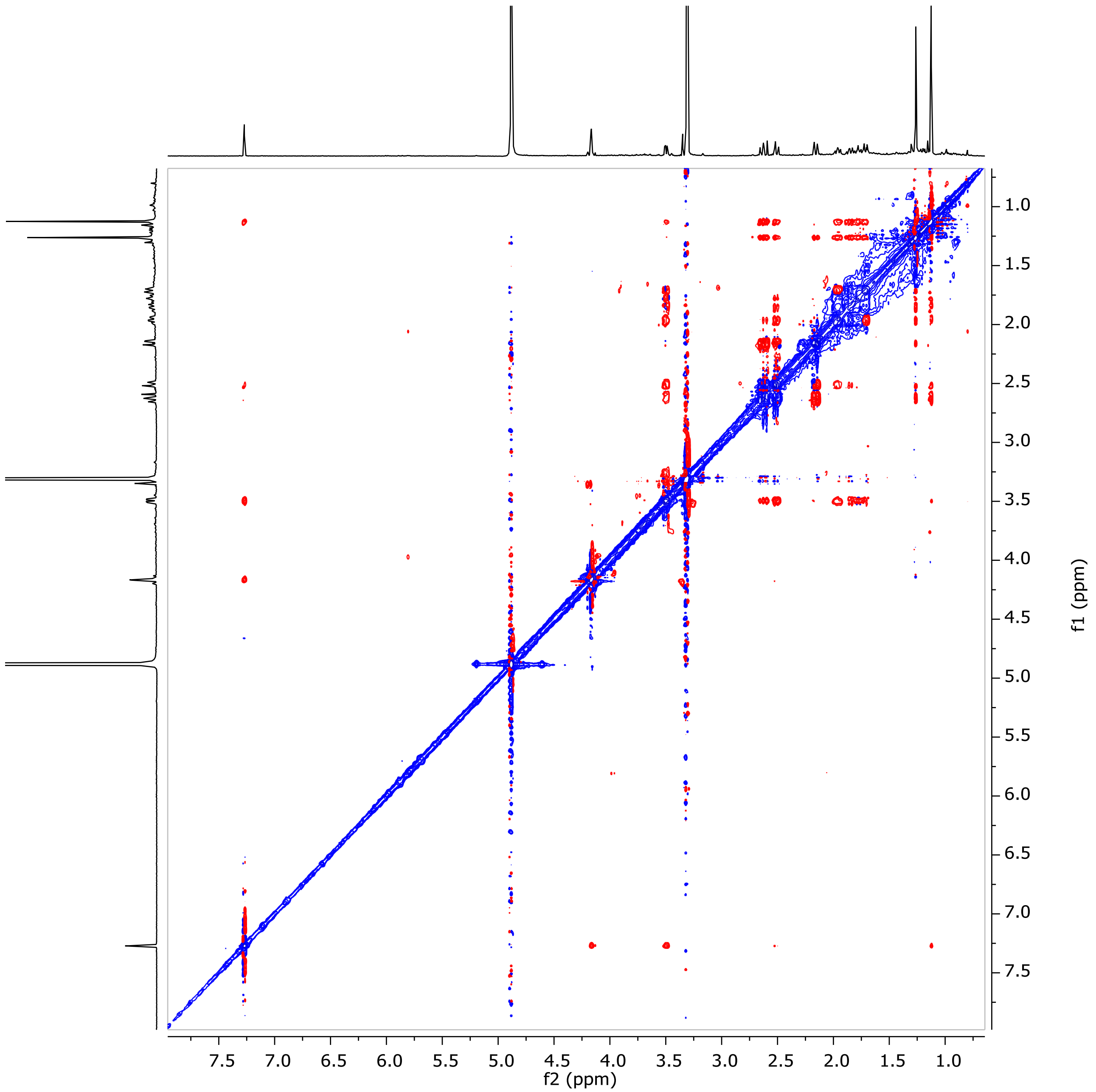


Figure S34. ROESY spectrum of **4** in methanol-$d_4$ at 500 MHz.

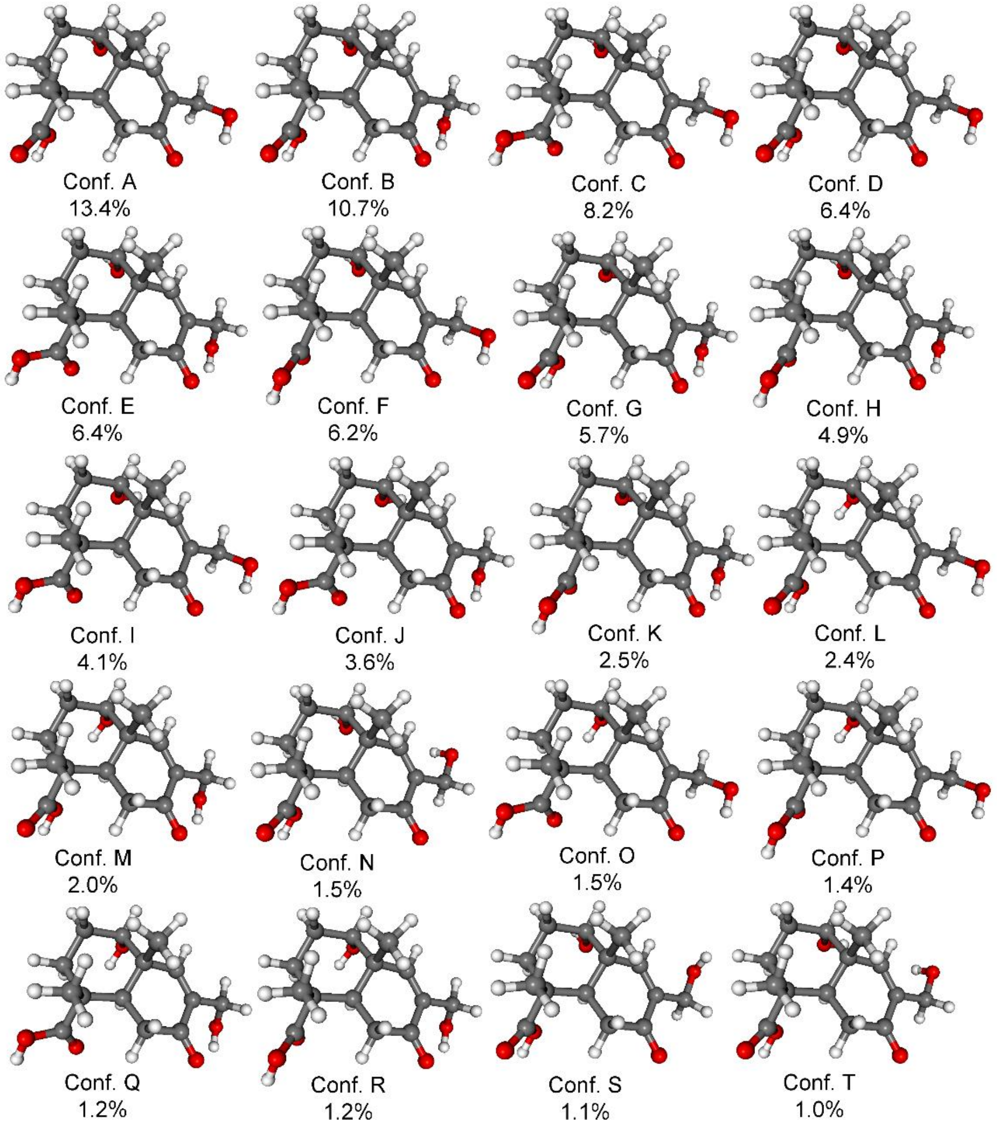


Figure S35. Low-energy ωB97X/TZVP PCM/MeOH conformers of (1*S*,4*R*,5*R*,10*R*)-**4**.

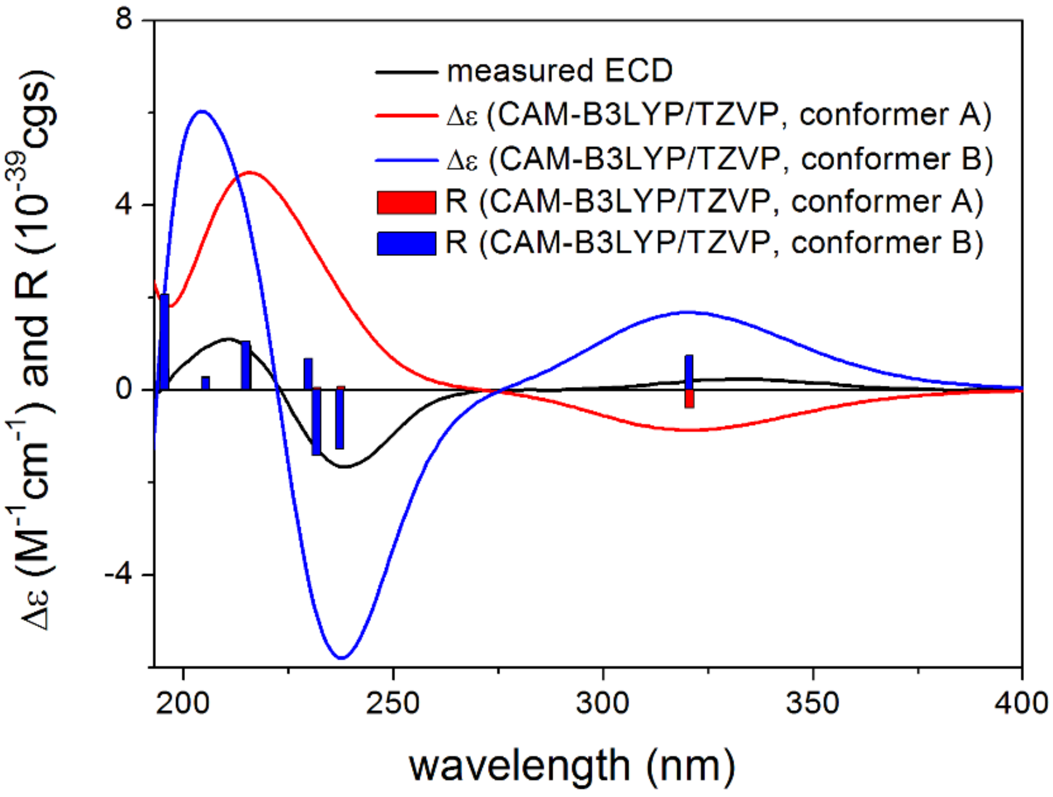


Figure S36. Experimental ECD spectrum of **4** (black) compared with the CAM-B3LYP/TZVP PCM/MeOH ECD spectra of the lowest-energy conformers of the two distinct conformer clusters of (1*S*,4*R*,5*R*,10*R*)-**4** (red: conformer A, blue: conformer B). Level of DFT optimization: ωB97X/TZVP PCM/MeOH. Bars represent the computed rotational strength values for conformers A (red) and B (blue).

# Generic Display Report

**Analysis Info**

| | | | |
|---|---|---|---|
| Analysis Name | \\Neon\MWISCOM\PEOPLE\sel22_Sherif Elsayed\Abundisporus\Amazon\A. violaceus AmaZon\Abund | Acquisition Date | 22.02.2022 17:12:57 |
| Method | B2F7_GA7_01_37146.d | Operator | esu |
| Sample Name | Abund B2F7 | Instrument | amaZon speed |
| Comment | | | |

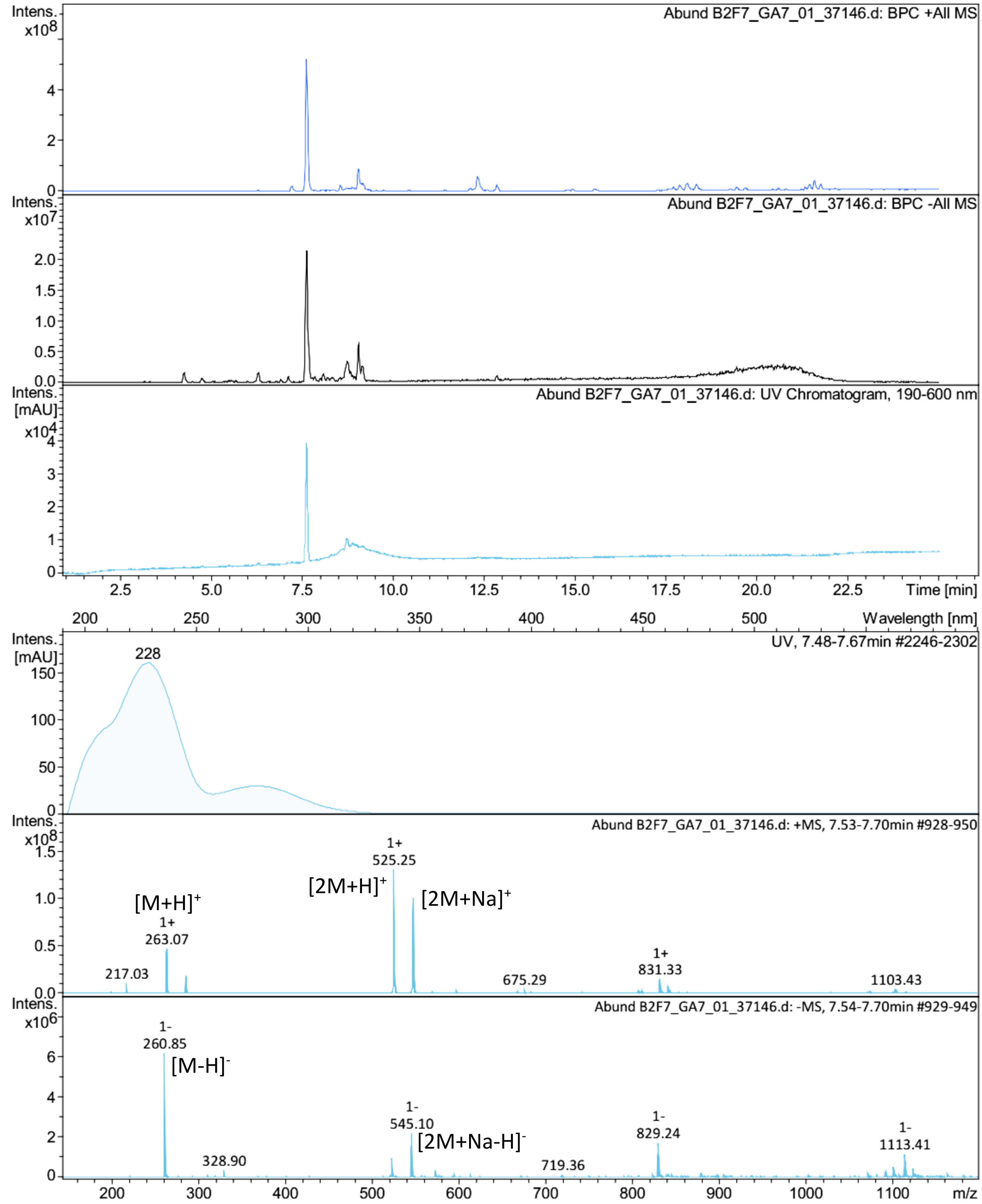




Figure S37. LRESIMS of **5**.

# Generic Display Report

| **Analysis Info** | | Acquisition Date | 28.02.2022 13:20:43 |
|---|---|---|---|
| Analysis Name | \\Neon\MWISCOM\PEOPLE\sel22_Sherif Elsayed\Abundisporus\Maxis\A. violaceus MaXis\Abund B1F11_P1-B-1_01_9618.d | | |
| Method | pos_screening_ms_100_2500_line.m | Operator | ate06 |
| Sample Name | Abund B1F11 | Instrument | maXis |
| Comment | Screening01<br>Waters Acquity UPLC BEH C_18 1,7um 2.1x50mm | | |

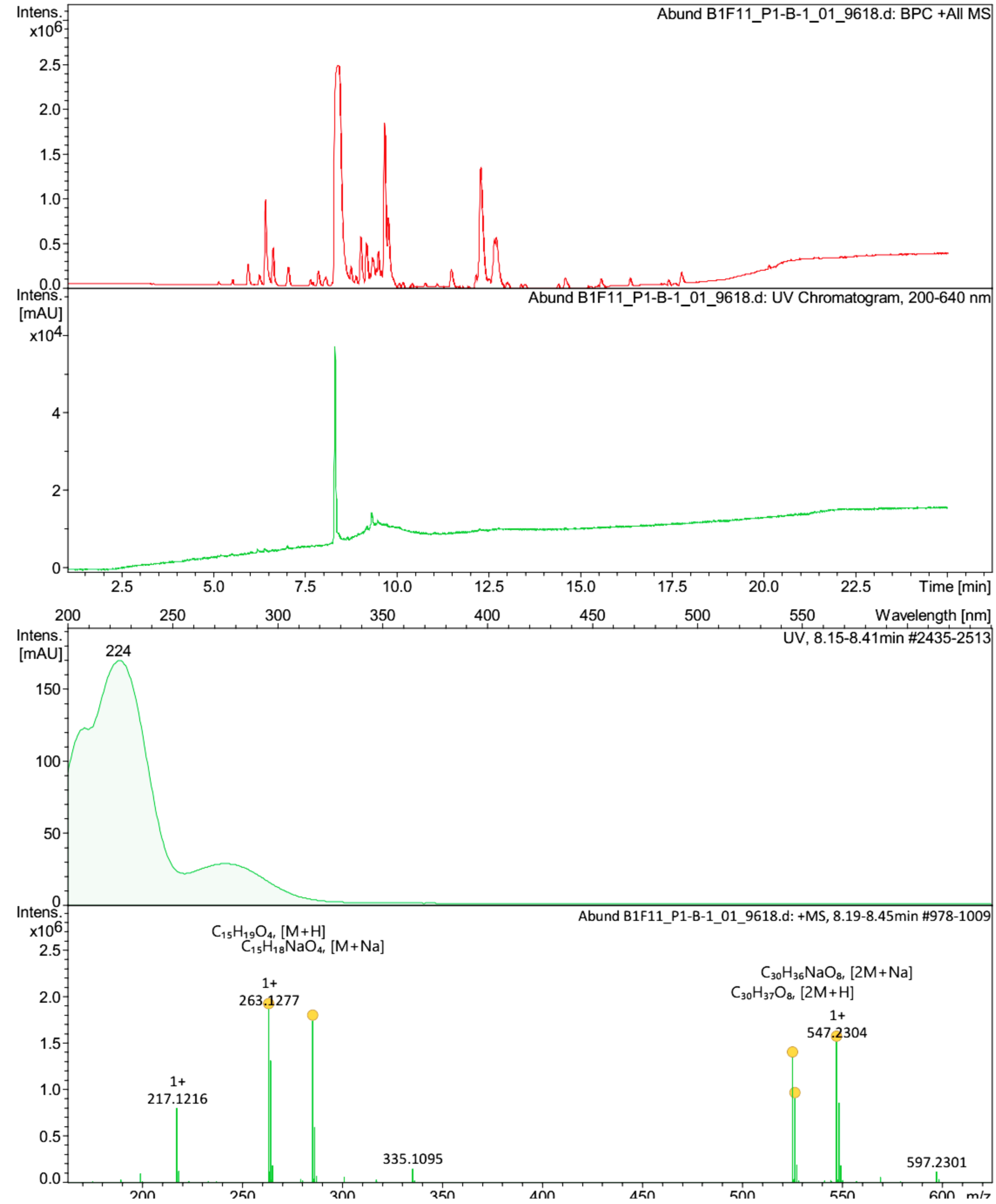




Figure S38. HRESIMS of **5**.

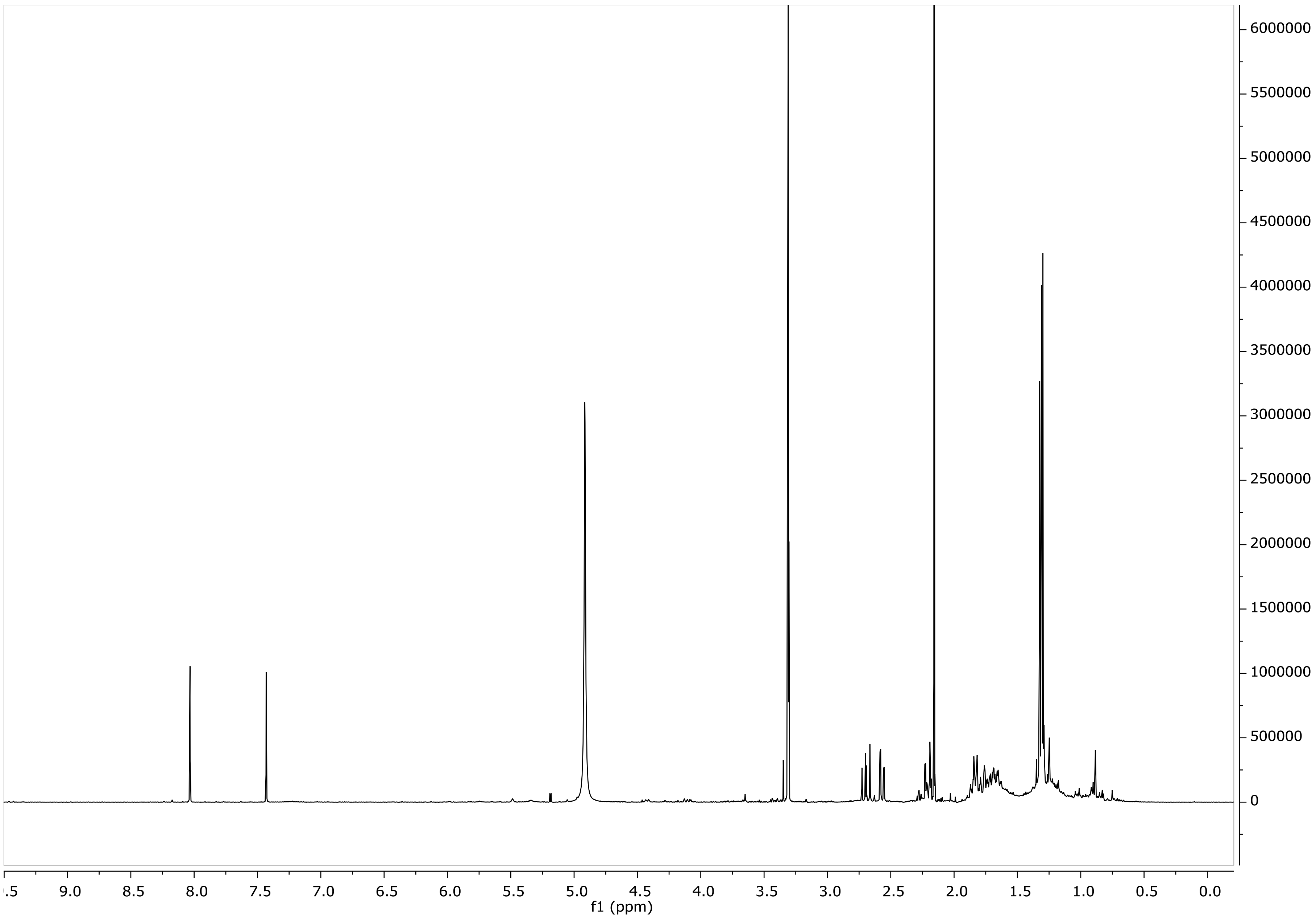


Figure S39. $^1$H NMR spectrum of **5** in methanol-$d_4$ at 500 MHz.

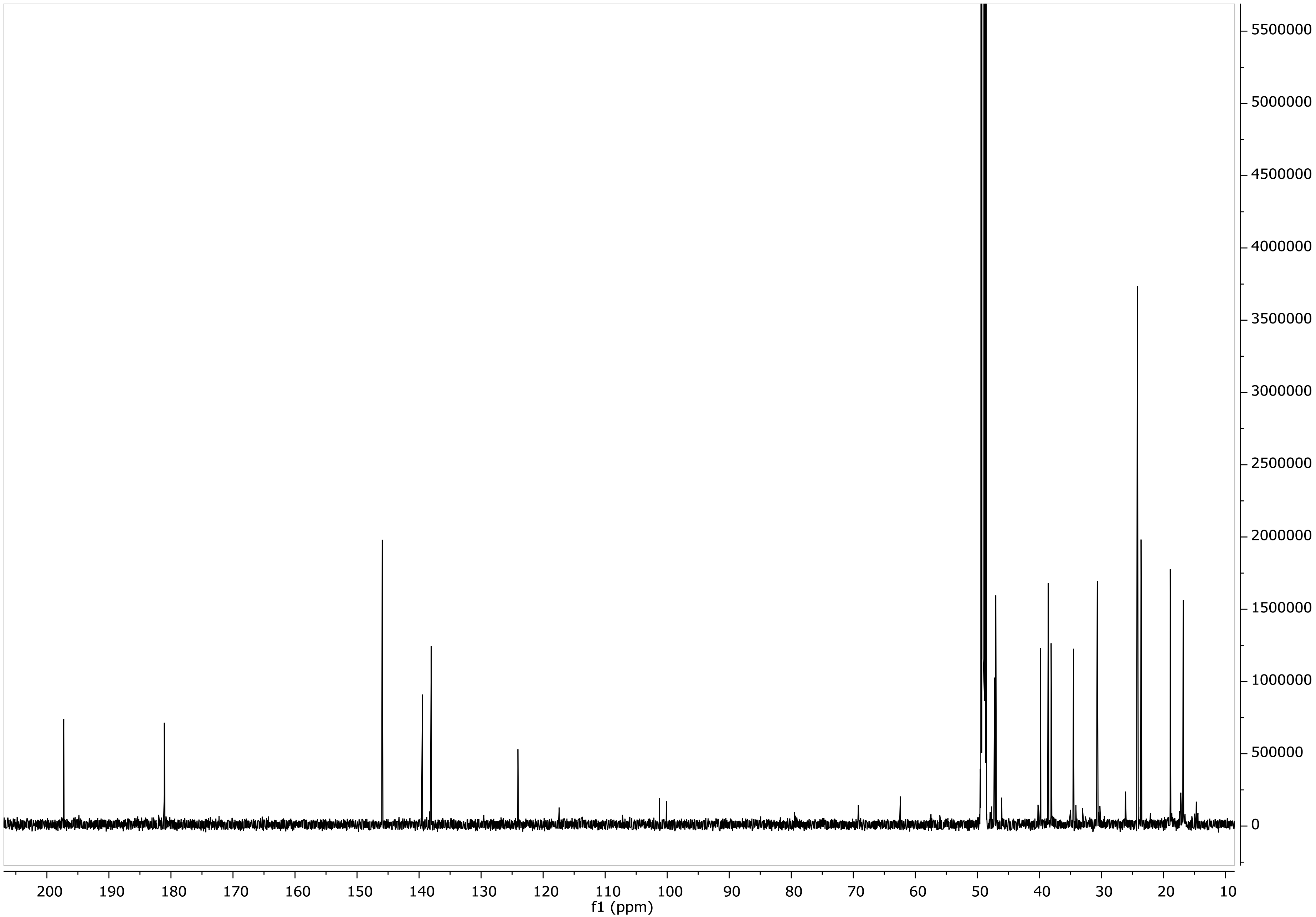


Figure S40. $^{13}C$ NMR spectrum of **5** in methanol-$d_4$ at 125 MHz.

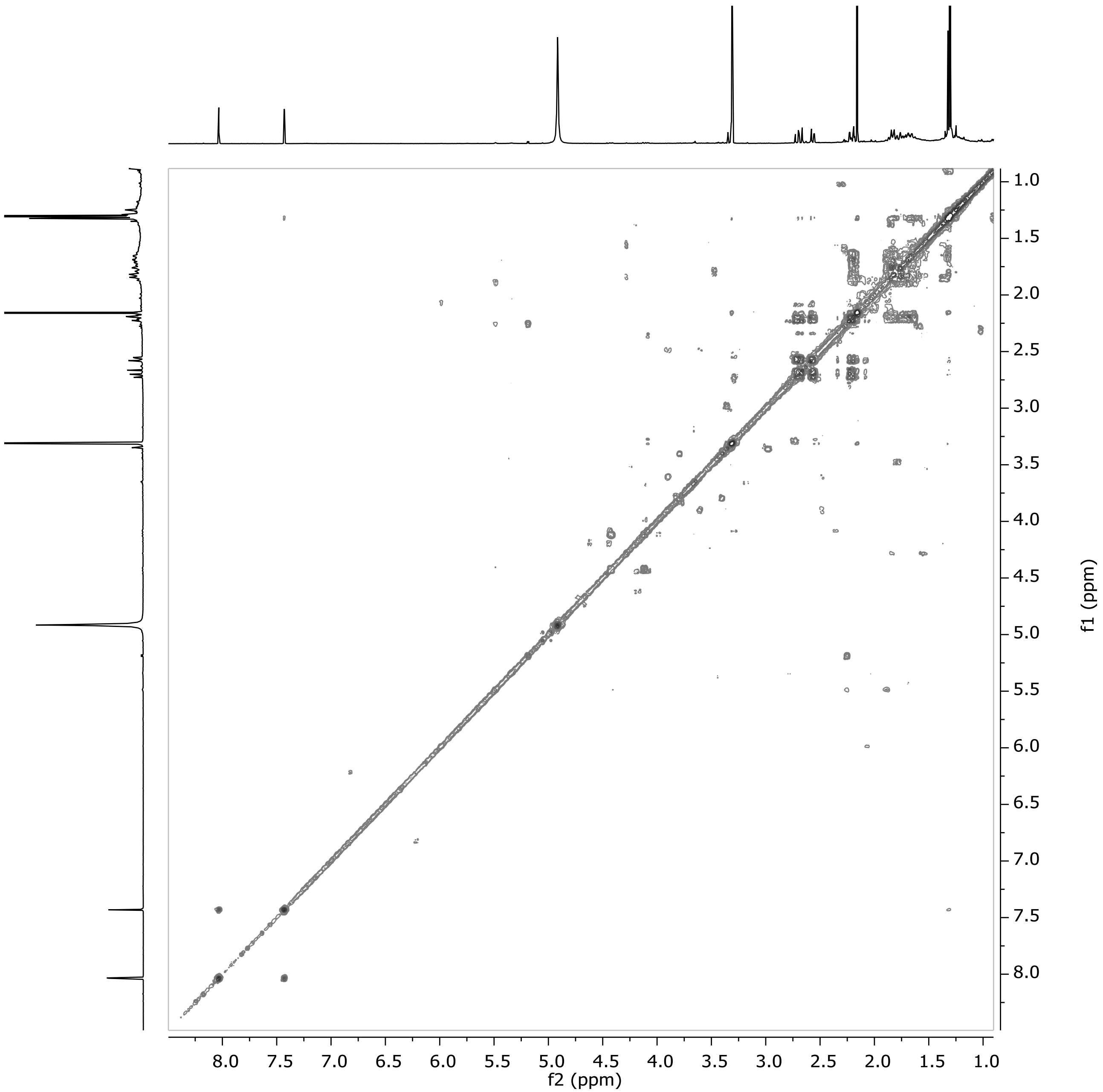


Figure S41. $^{1}H$-$^{1}H$ COSY spectrum of **5** in methanol-$d_4$ at 500 MHz.

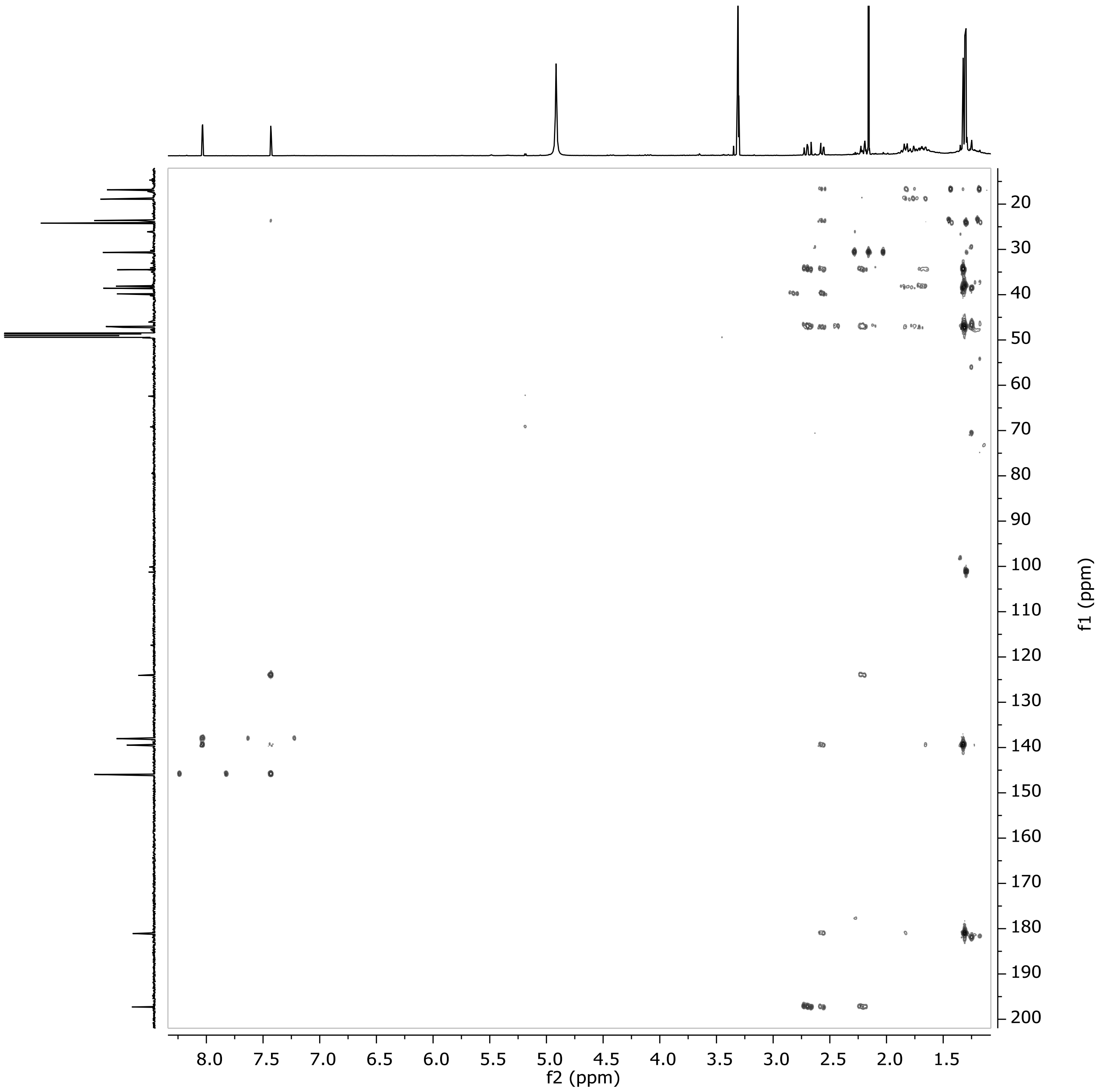


Figure S42. HMBC spectrum of **5** in methanol-$d_4$ at 500 MHz.

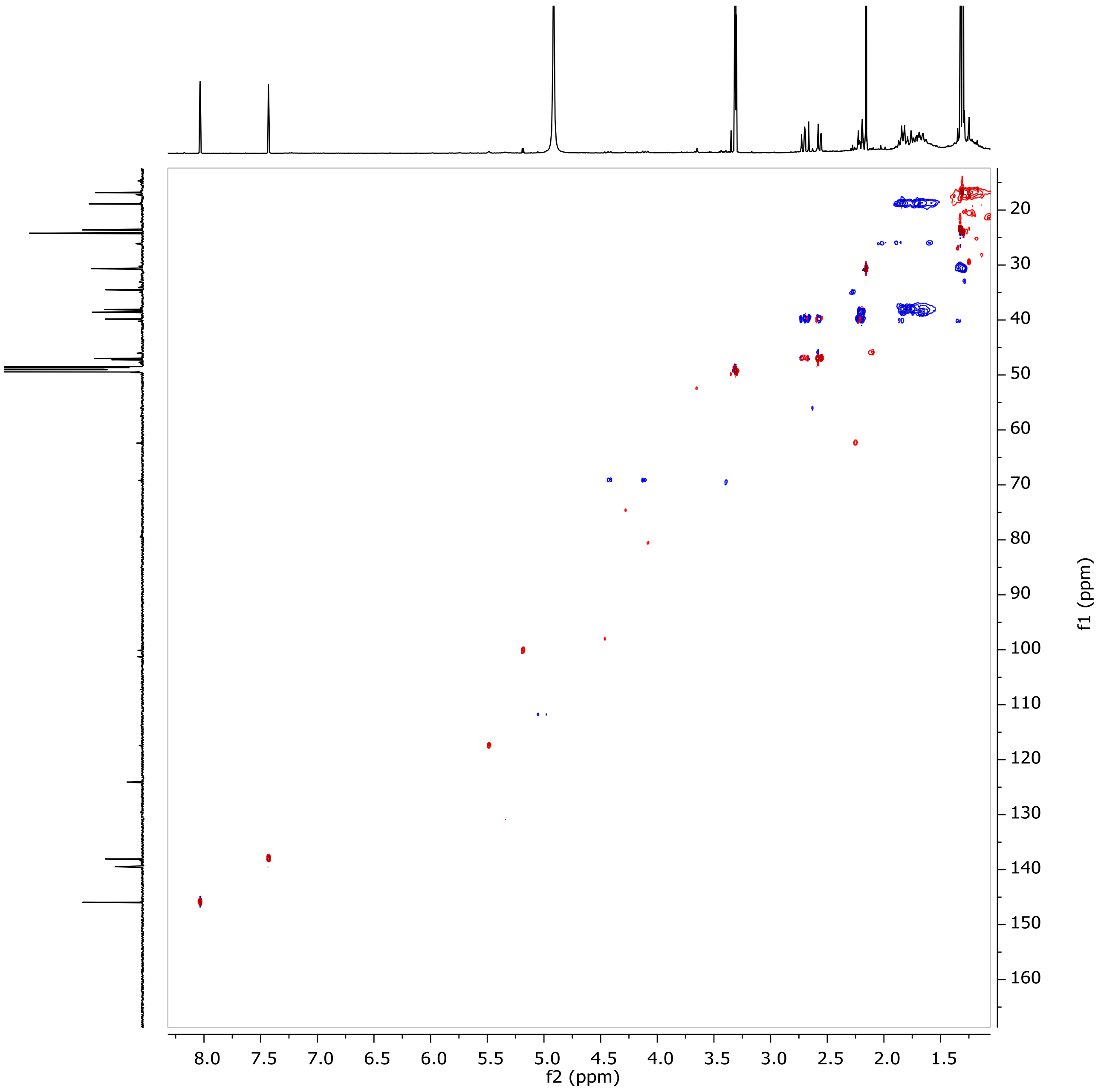


Figure S43. HSQC spectrum of **5** in methanol-$d_4$ at 500 MHz.

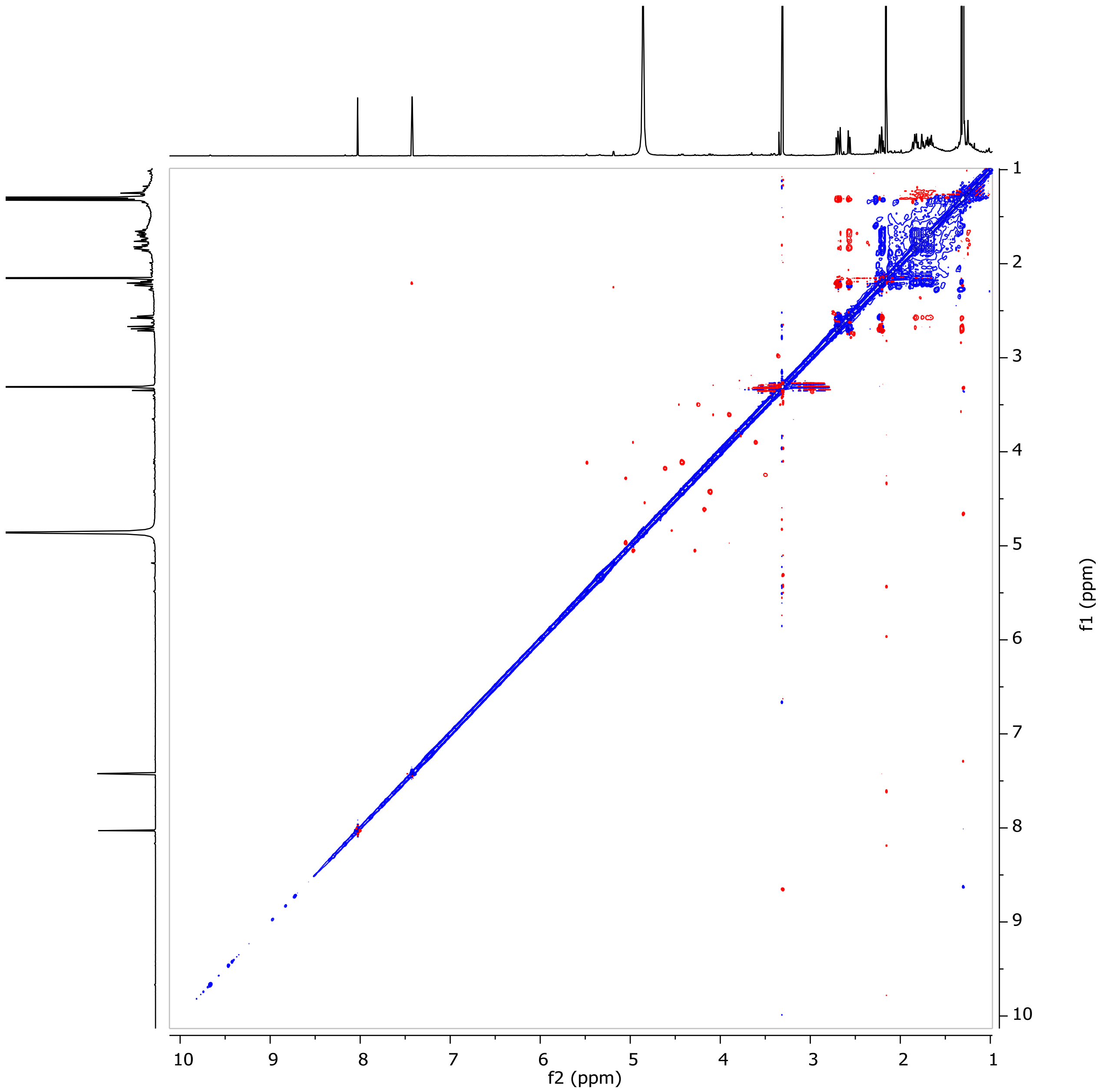


Figure S44. ROESY spectrum of **5** in methanol-$d_4$ at 700 MHz.

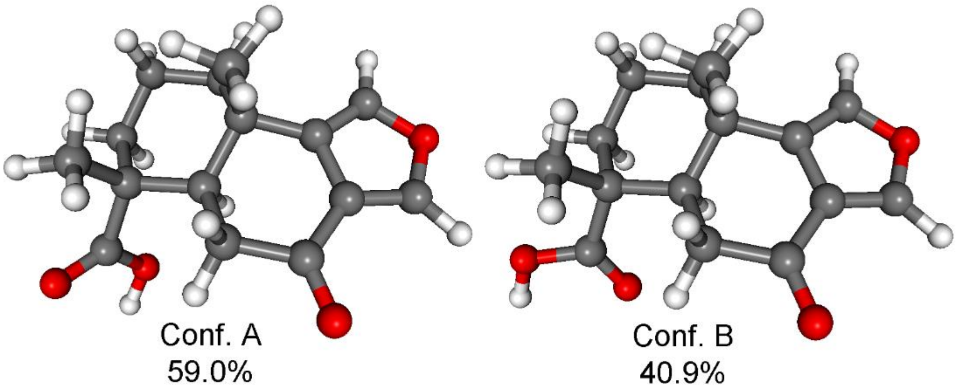


Figure S45. Low-energy ωB97X/TZVP PCM/MeOH conformers of (4*R*,5*R*,10*S*)-**5**.

# Generic Display Report

**Analysis Info**

Acquisition Date 02.03.2022 18:27:45

Analysis Name \\Neon\MWISCOM\PEOPLE\sel22_Sherif Elsayed\Abundisporus\Amazon\A. violaceus AmaZon\Abund B1F9F2_BB6_01_10300.d

Method Screening_Set05_iso-uv210_10300.m

Operator lab

Sample Name Abund B1F9F2

Instrument amaZon speed

Comment

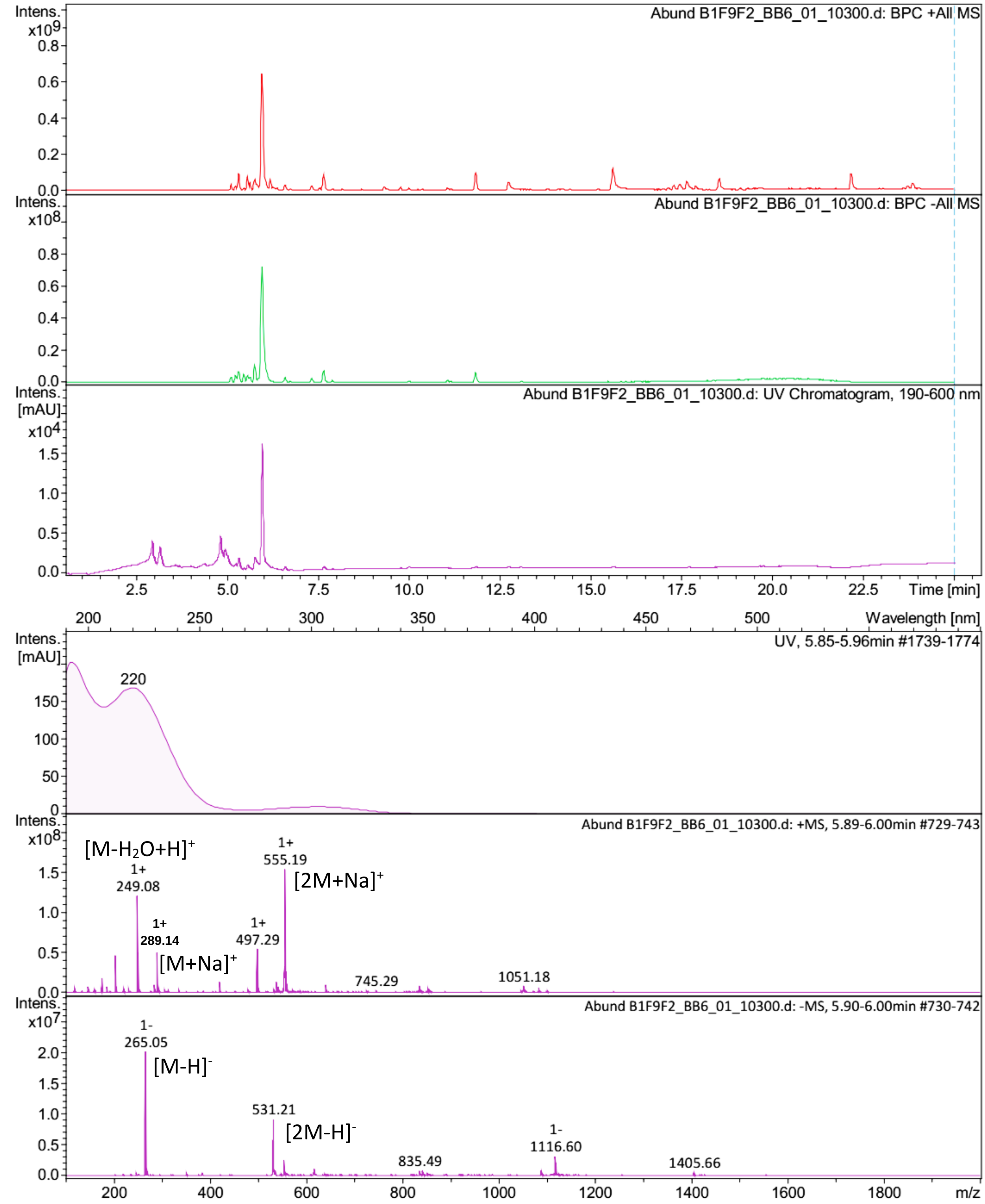




Figure S46. LRESIMS of **6**.

# Generic Display Report

**Analysis Info**

| | | | |
|---|---|---|---|
| | | Acquisition Date | 09.03.2022 12:43:03 |
| Analysis Name | \\Neon\MWISCOM\PEOPLE\sel22_Sherif Elsayed\Abundisporus\Maxis\A. violaceus MaXis\Abund B1F9F2_P1-B-3_01_9679.d | | |
| Method | pos_saure_15000_screening_ms_100_2500_line.m | Operator | ate06 |
| Sample Name | Abund B1F9F2 | Instrument | maXis |
| Comment | Screening01<br>Waters Acquity UPLC BEH C_18 1,7um 2.1x50mm | | |

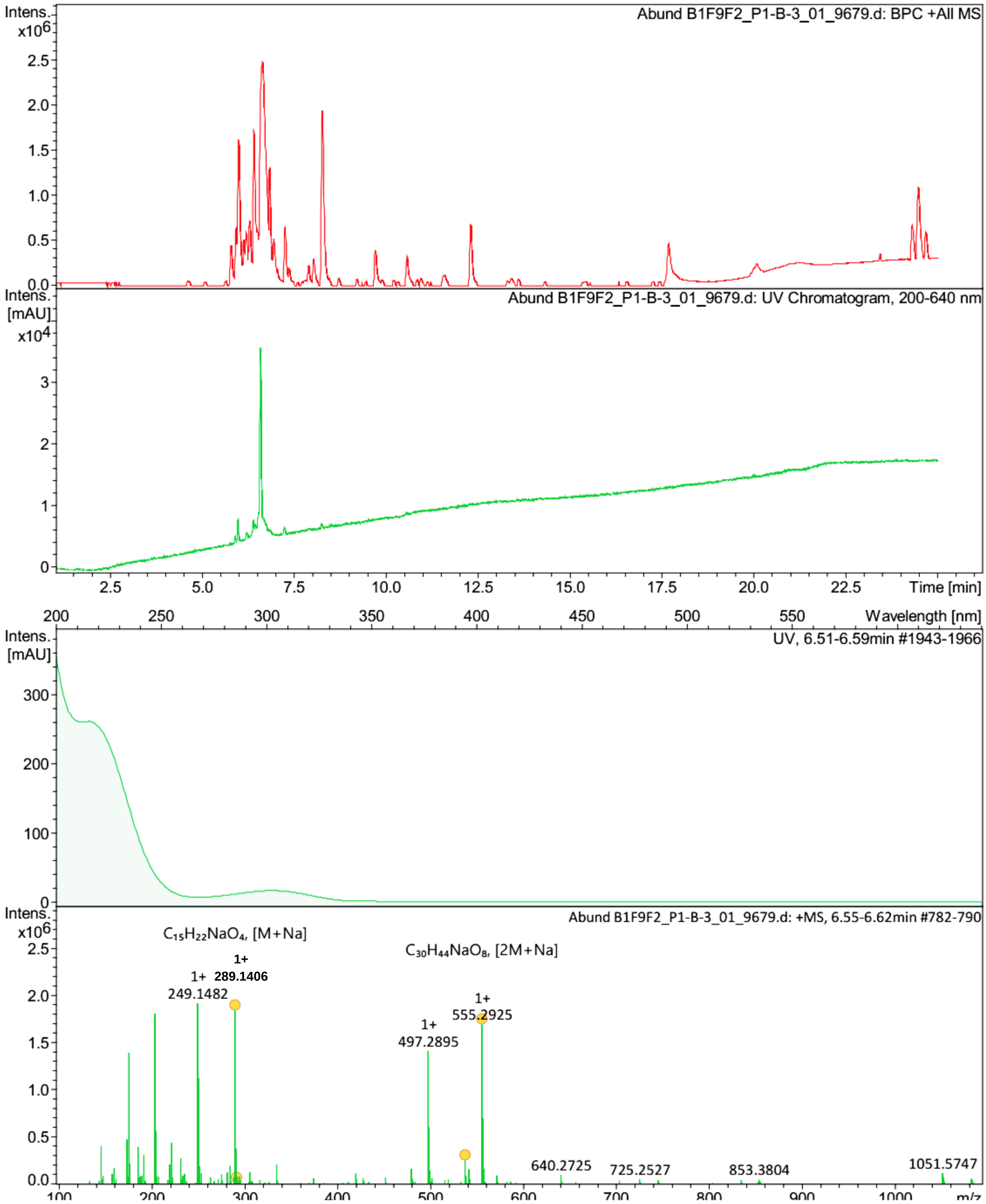


Bruker Compass DataAnalysis 4.4 printed: 11.04.2023 18:40:11 by: mk-mwisnmr Page 1 of 1

Figure S47. HRESIMS of **6**.

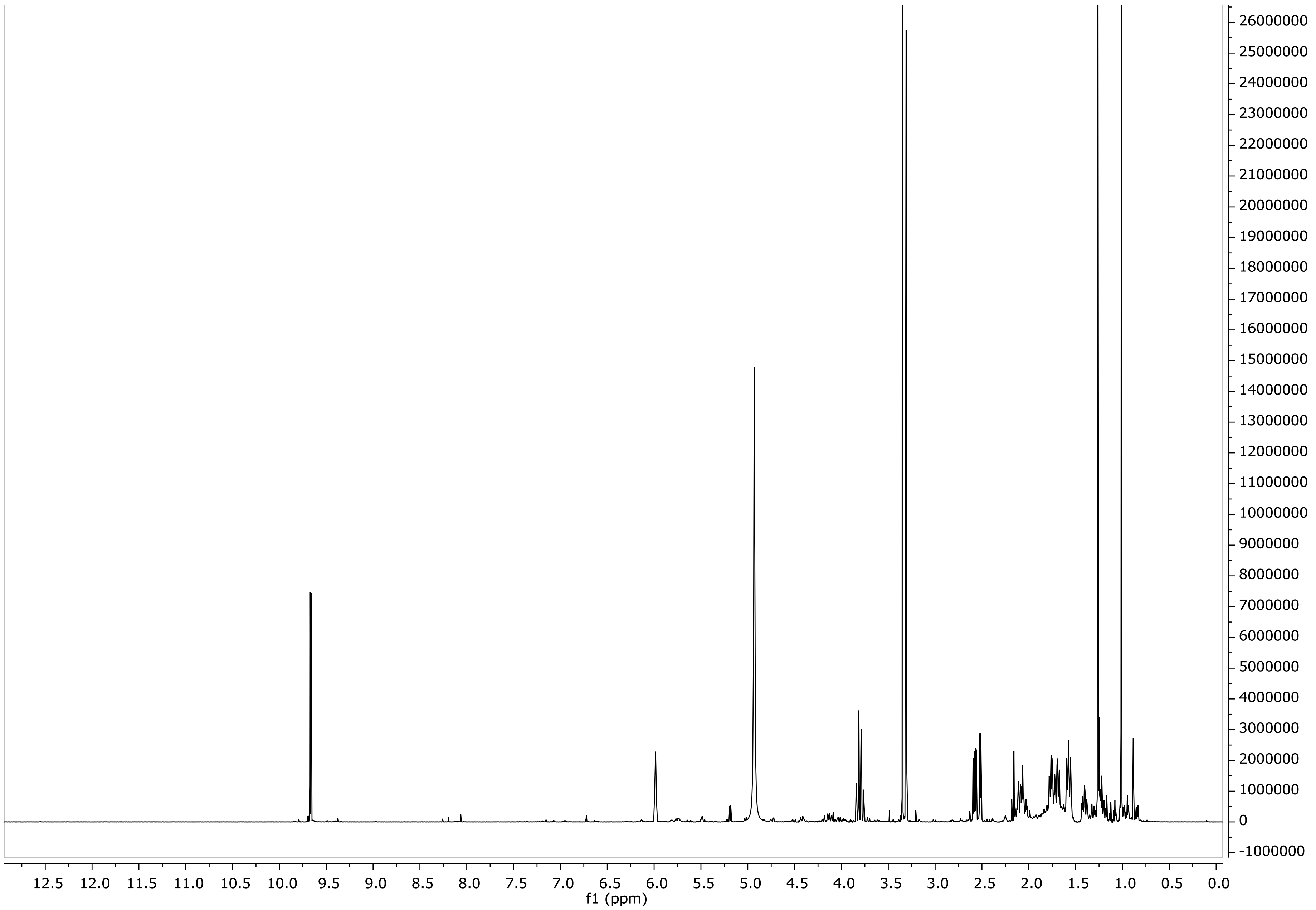


Figure S48. $^{1}$H NMR spectrum of **6** in methanol-$d_4$ at 500 MHz.

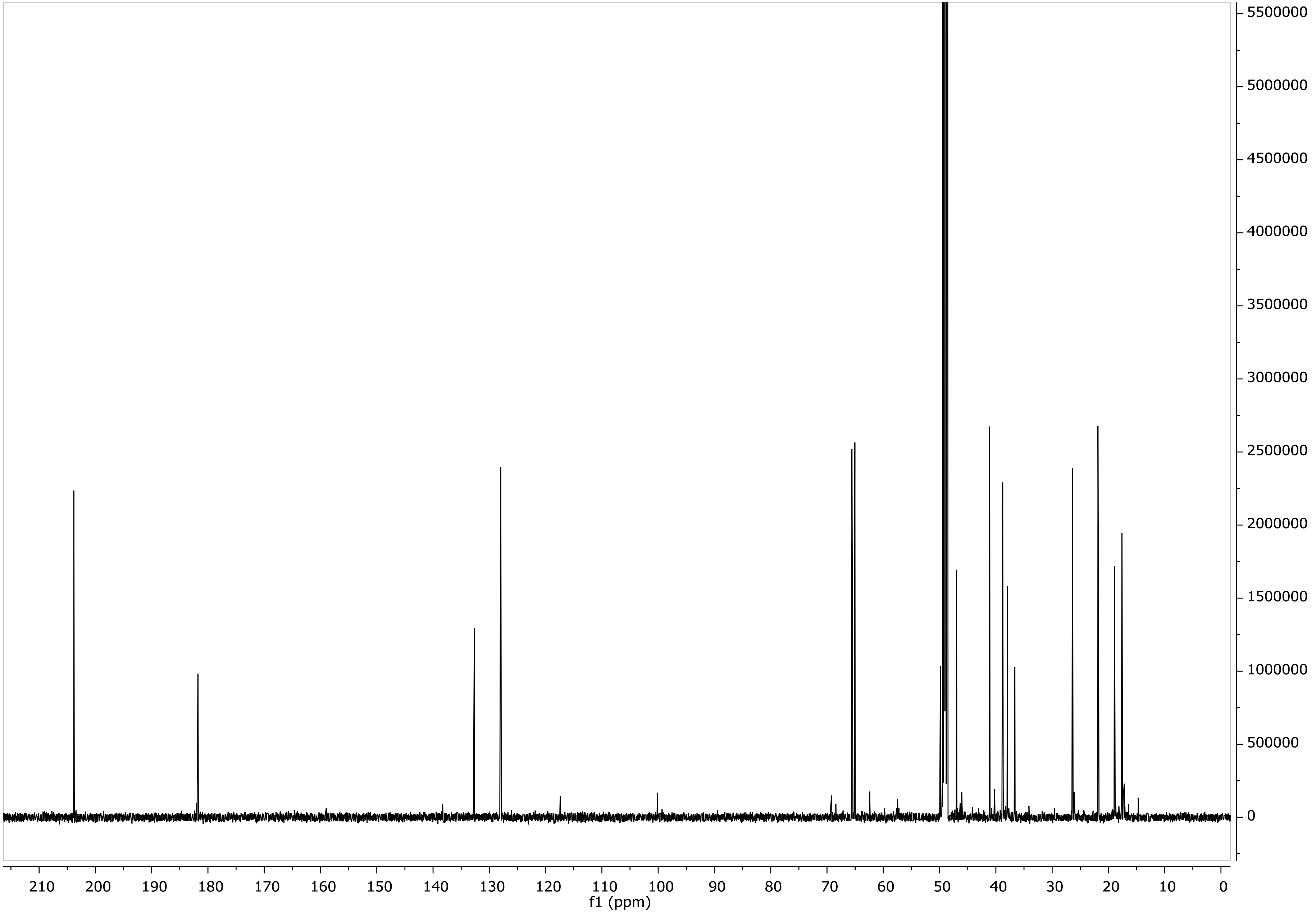


Figure S49. $^{13}$C NMR spectrum of **6** in methanol-$d_4$ at 125 MHz.

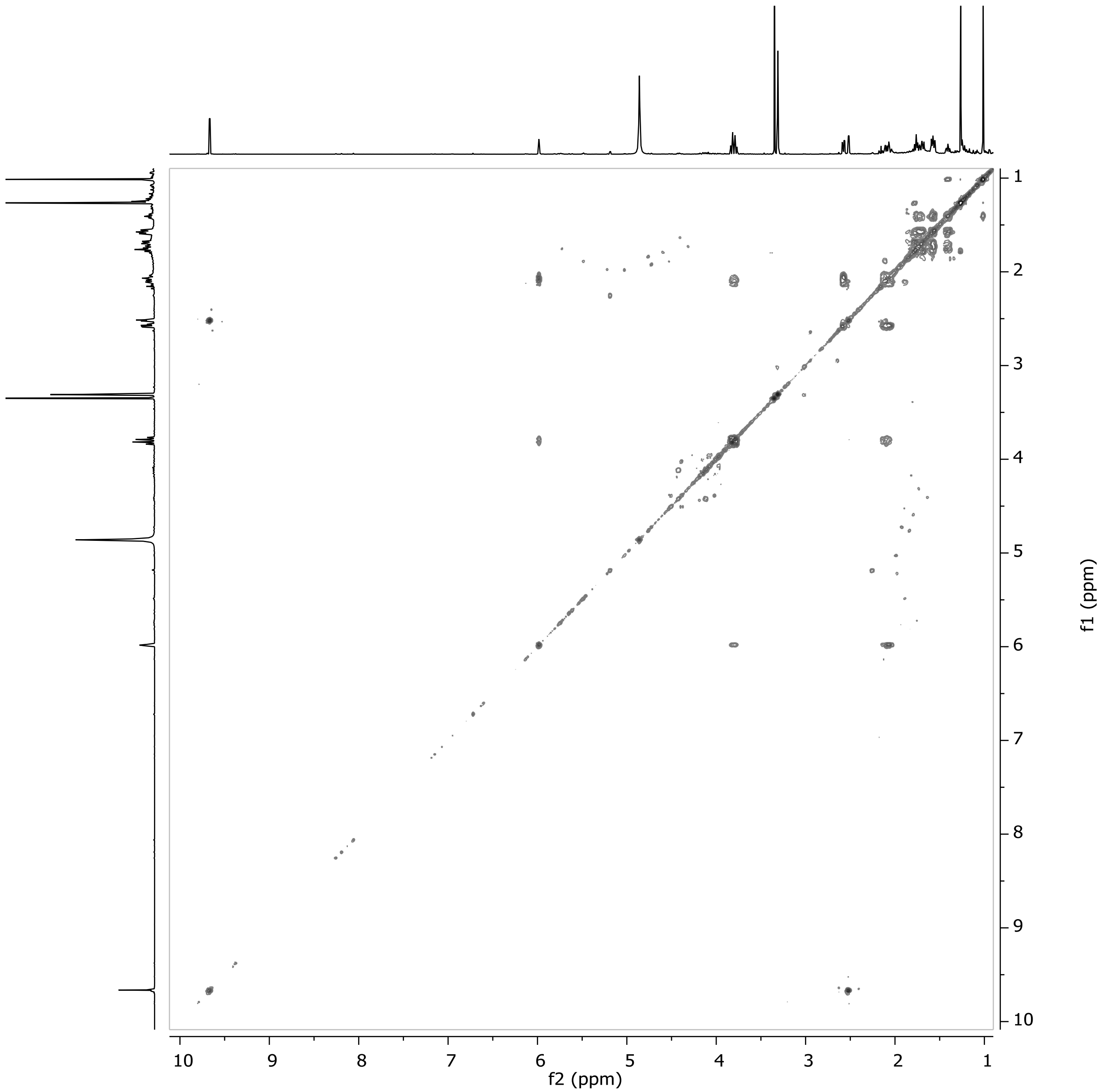


Figure S50. $^1H$-$^1H$ COSY spectrum of **6** in methanol-$d_4$ at 500 MHz.

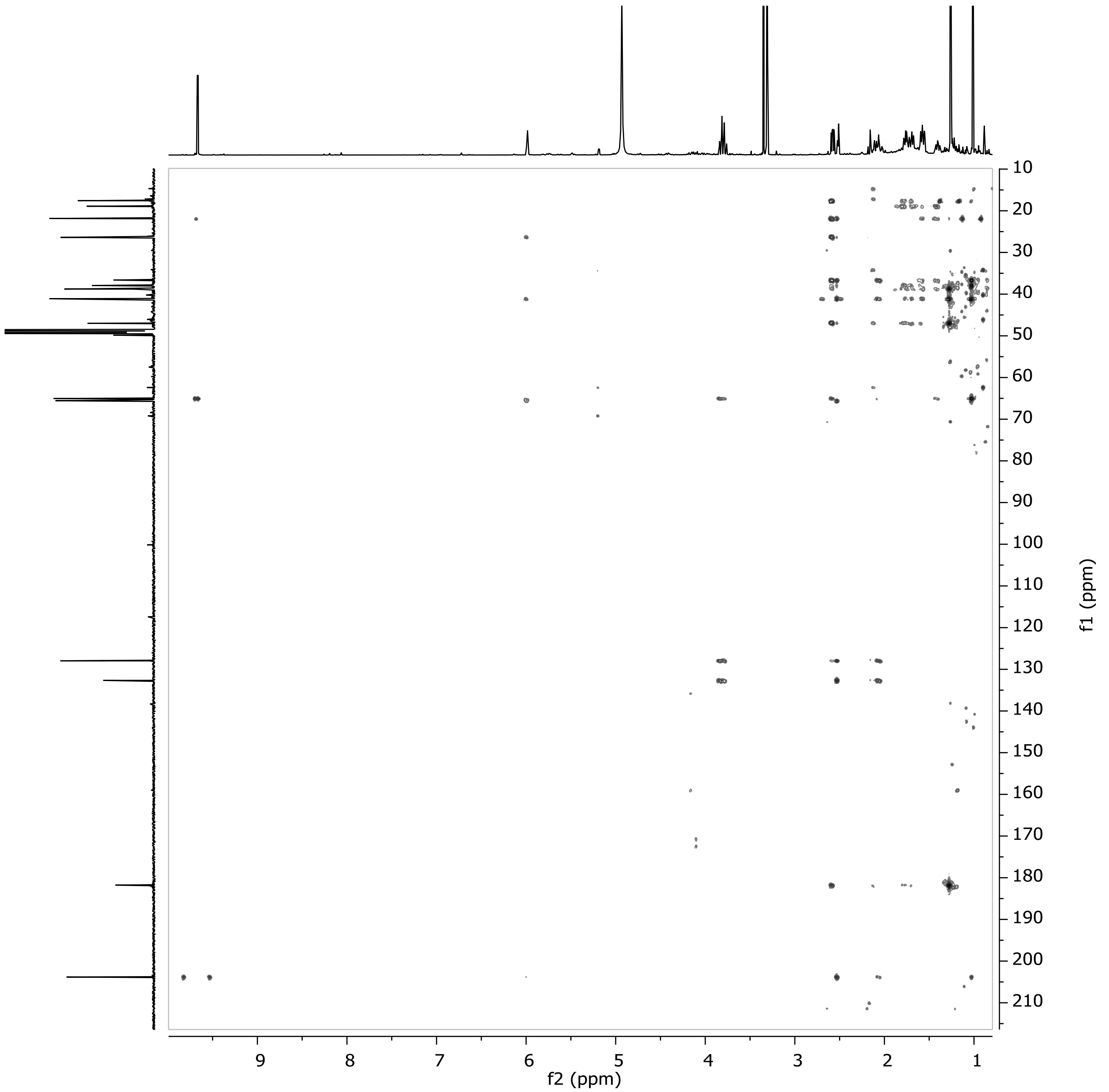


Figure S51. HMBC spectrum of **6** in methanol-$d_4$ at 500 MHz.

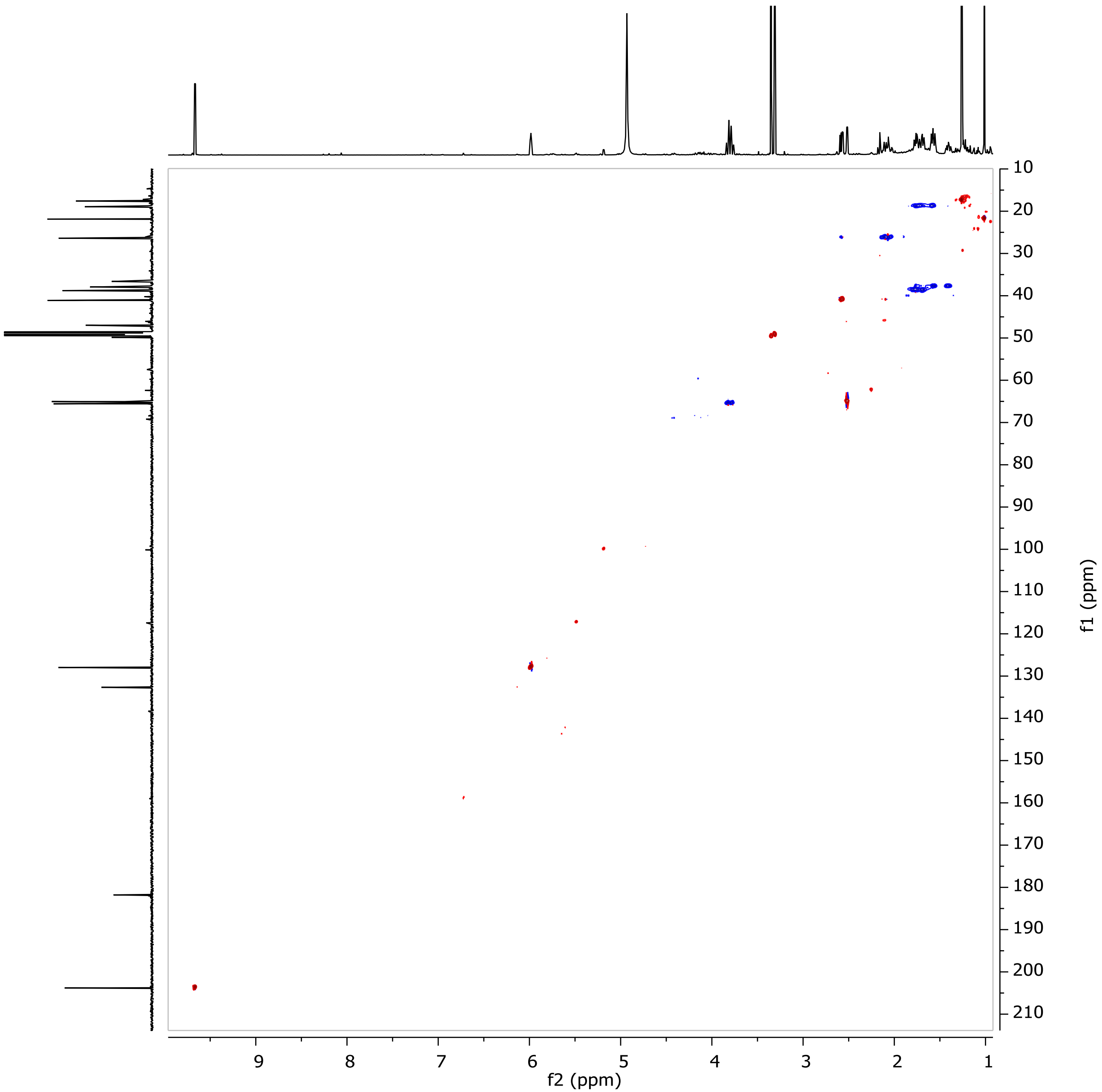


Figure S52. HSQC spectrum of **6** in methanol-*$d_4$* at 500 MHz.

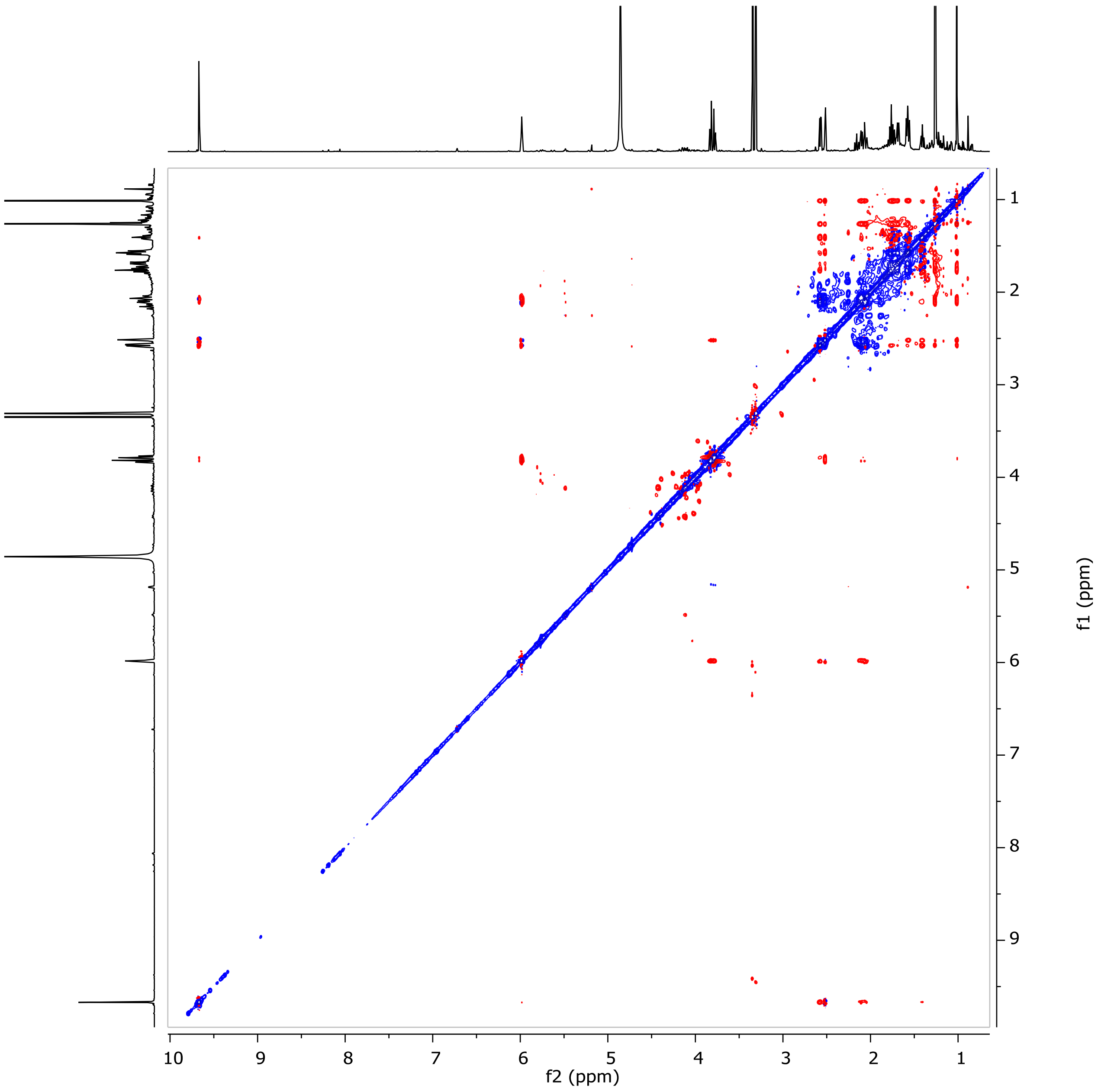


Figure S53. ROESY spectrum of **6** in methanol-$d_4$ at 700 MHz.

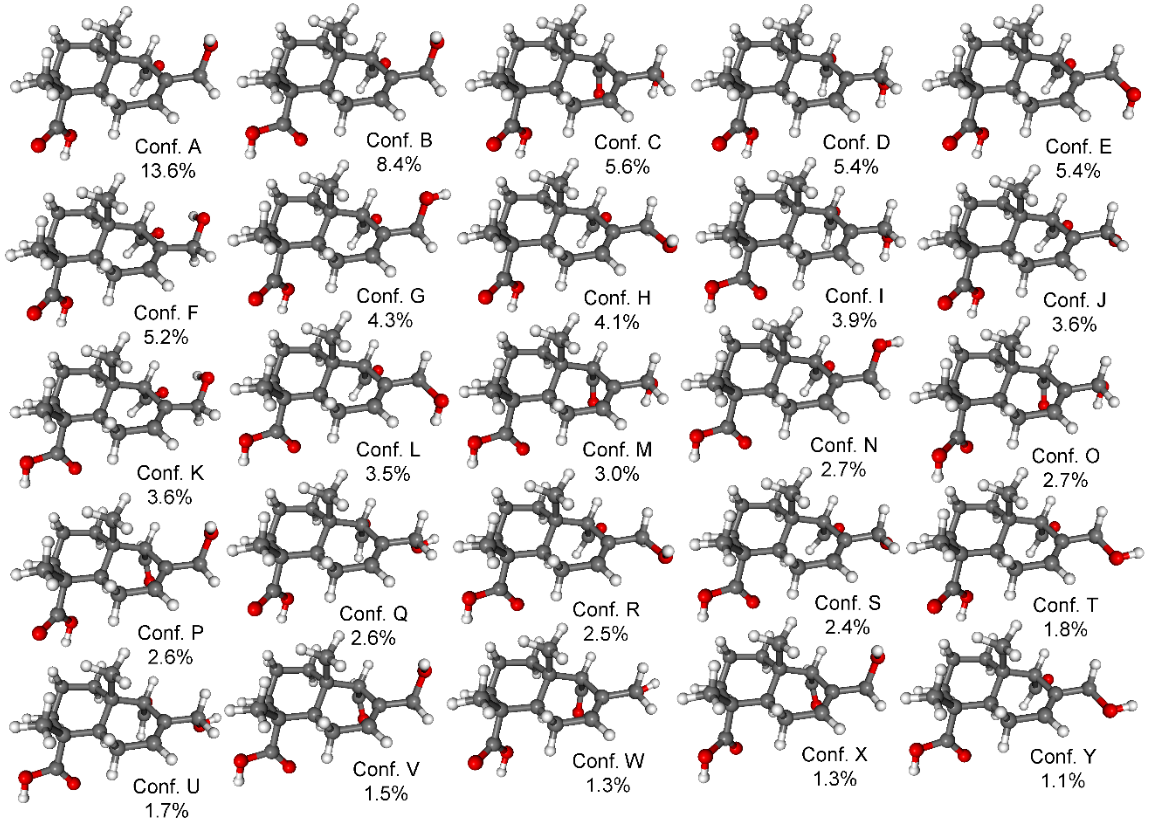


Figure S54. Low-energy ωB97X/TZVP PCM/MeOH conformers of (4*R*,5*R*,9*S*,10*S*)-**6**.

# Generic Display Report

**Analysis Info**

| | | | |
|---|---|---|---|
| Analysis Name | \\Neon\MWISCOM\PEOPLE\sel22_Sherif Elsayed\Abundisporus\Amazon\A. violaceus AmaZon\Abund | Acquisition Date | 02.03.2022 09:24:39 |
| Method | [illegible]_10285.m | Operator | lab |
| Sample Name | Abund B1F13F1 | Instrument | amaZon speed |
| Comment | | | |

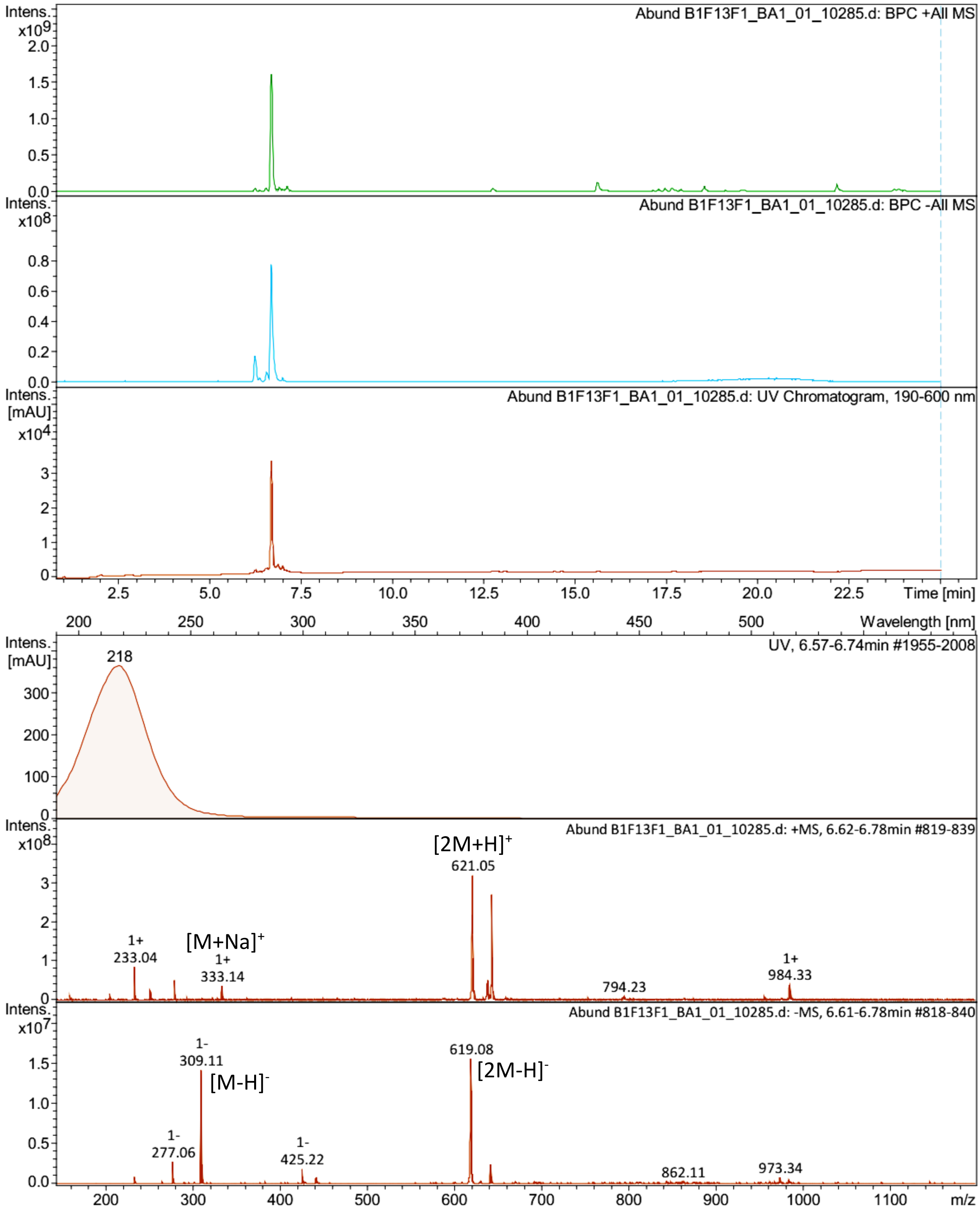


Bruker Compass DataAnalysis 4.4 printed: 11.04.2023 18:19:29 by: mk-mwisnmr Page 1 of 1

Figure S55. LRESIMS of **7**.

# Generic Display Report

**Analysis Info**

| | | | |
|---|---|---|---|
| Analysis Name | F:\Volume D\HZI Data\Winnie\Abundisporus\A violaceus MaXis\(310) | Acquisition Date | 09.03.2022 14:15:56 |
| Method | pos_säure_10000_screening_ms_100_2500_line.m | Operator | ate06 |
| Sample Name | Abund B1F13F1 | Instrument | maXis |
| Comment | Screening01<br>Waters Acquity UPLC BEH C_18 1,7um 2.1x50mm | | |

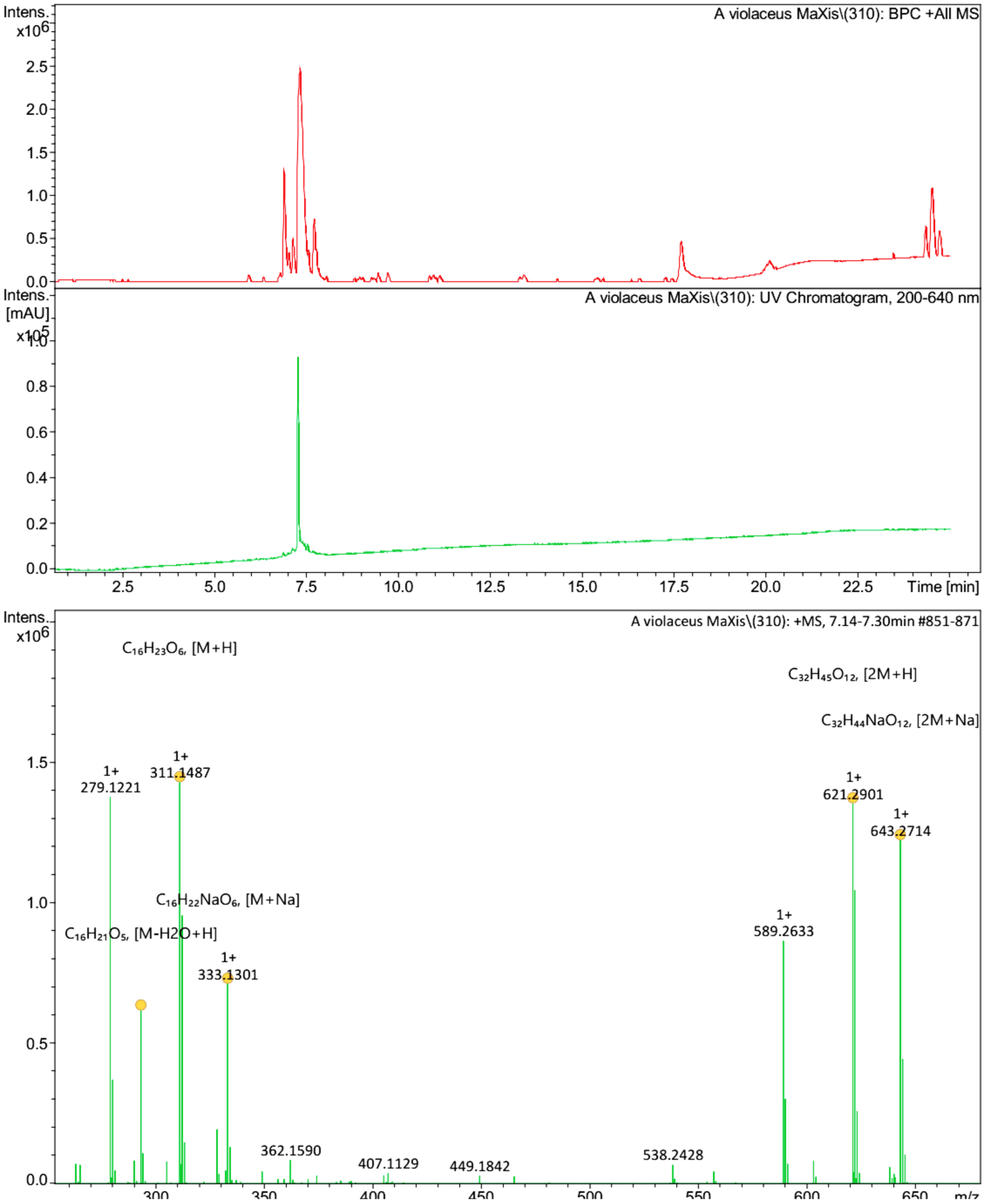


Bruker Compass DataAnalysis 4.4 printed: 05.04.2023 11:17:25 by: sel22 Page 1 of 1

Figure S56. HRESIMS of **7**.

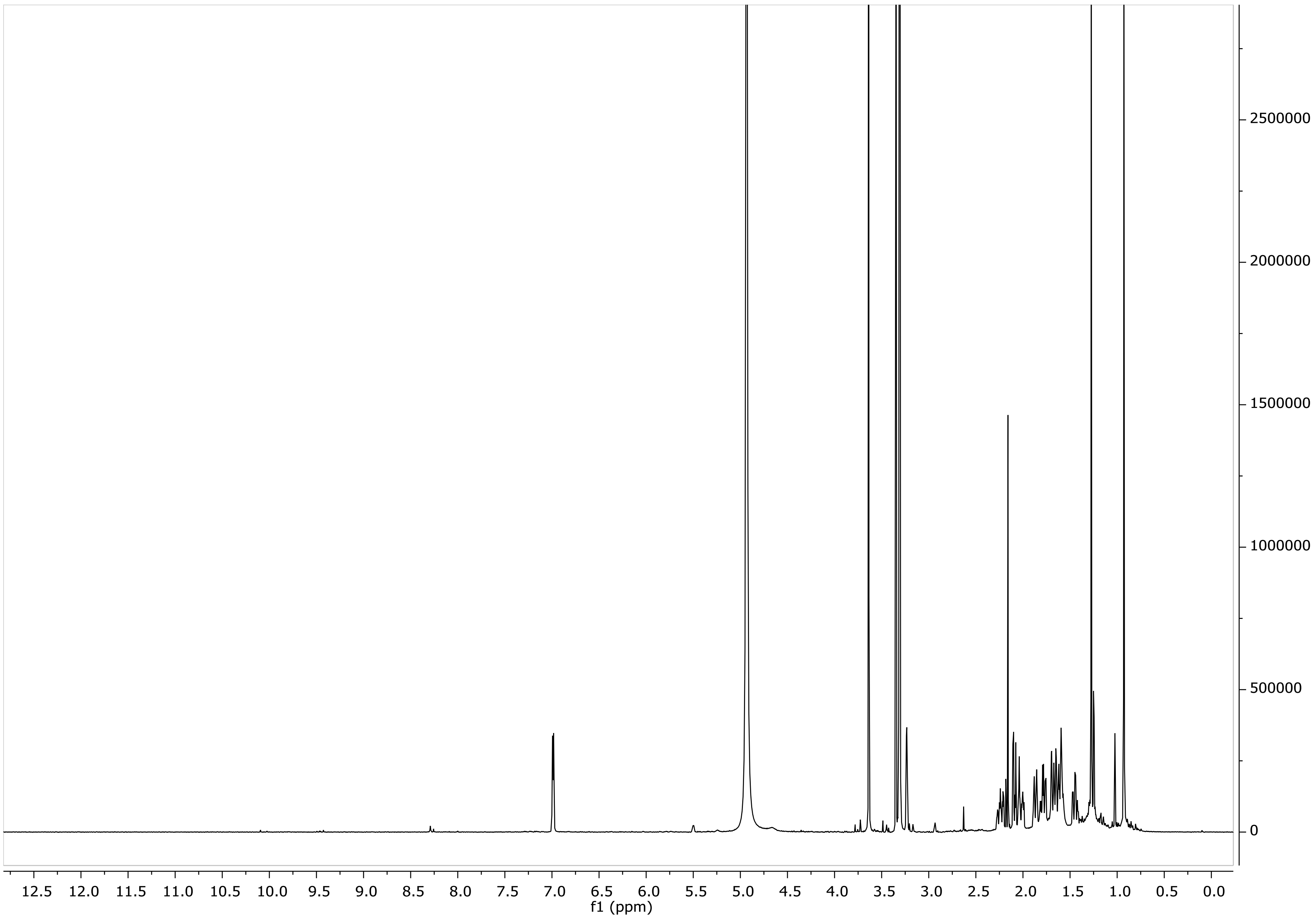


Figure S57. $^{1}H$ NMR spectrum of **7** in methanol-$d_4$ at 500 MHz.

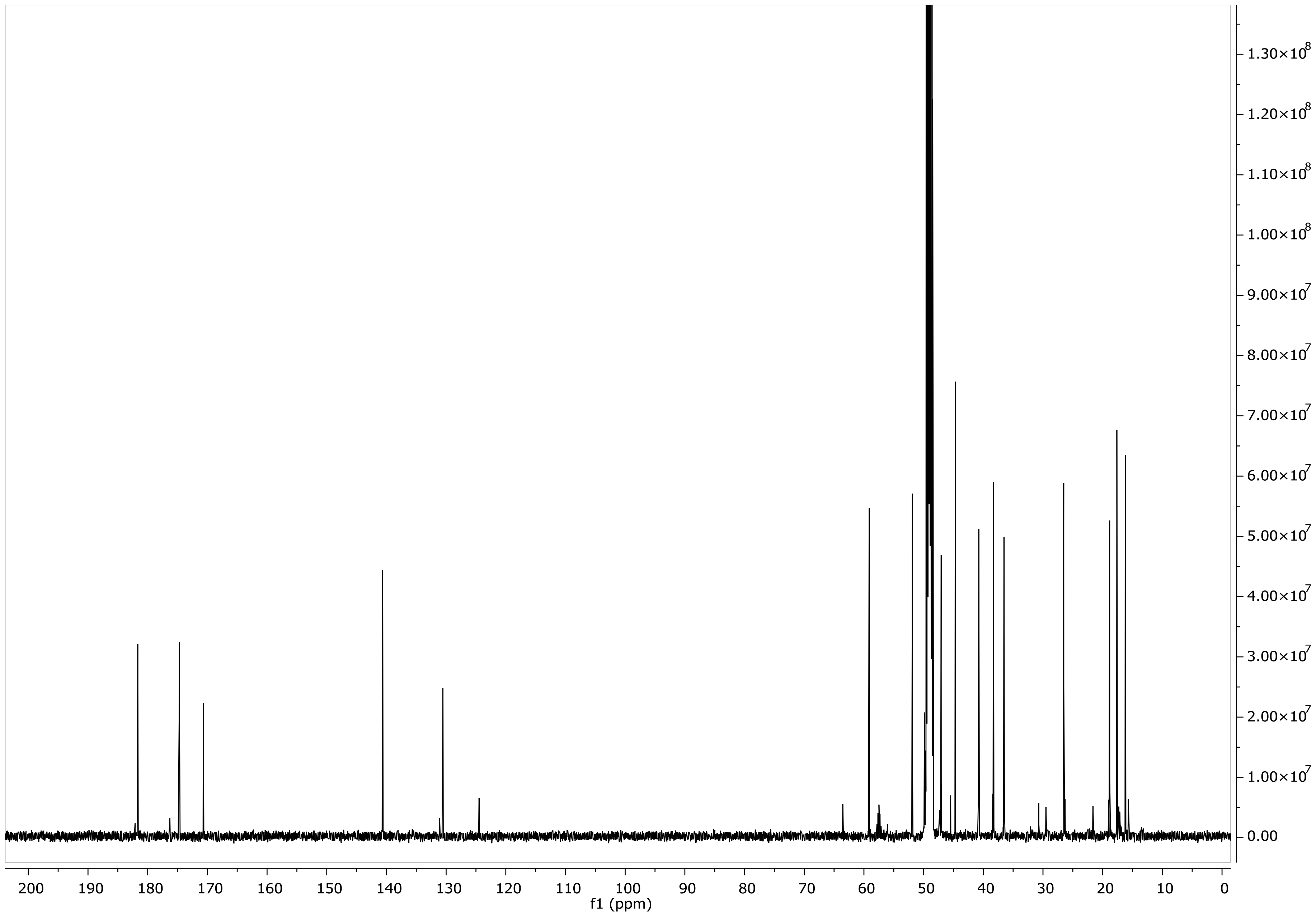


Figure S58. $^{13}$C NMR spectrum of **7** in methanol-$d_4$ at 125 MHz.

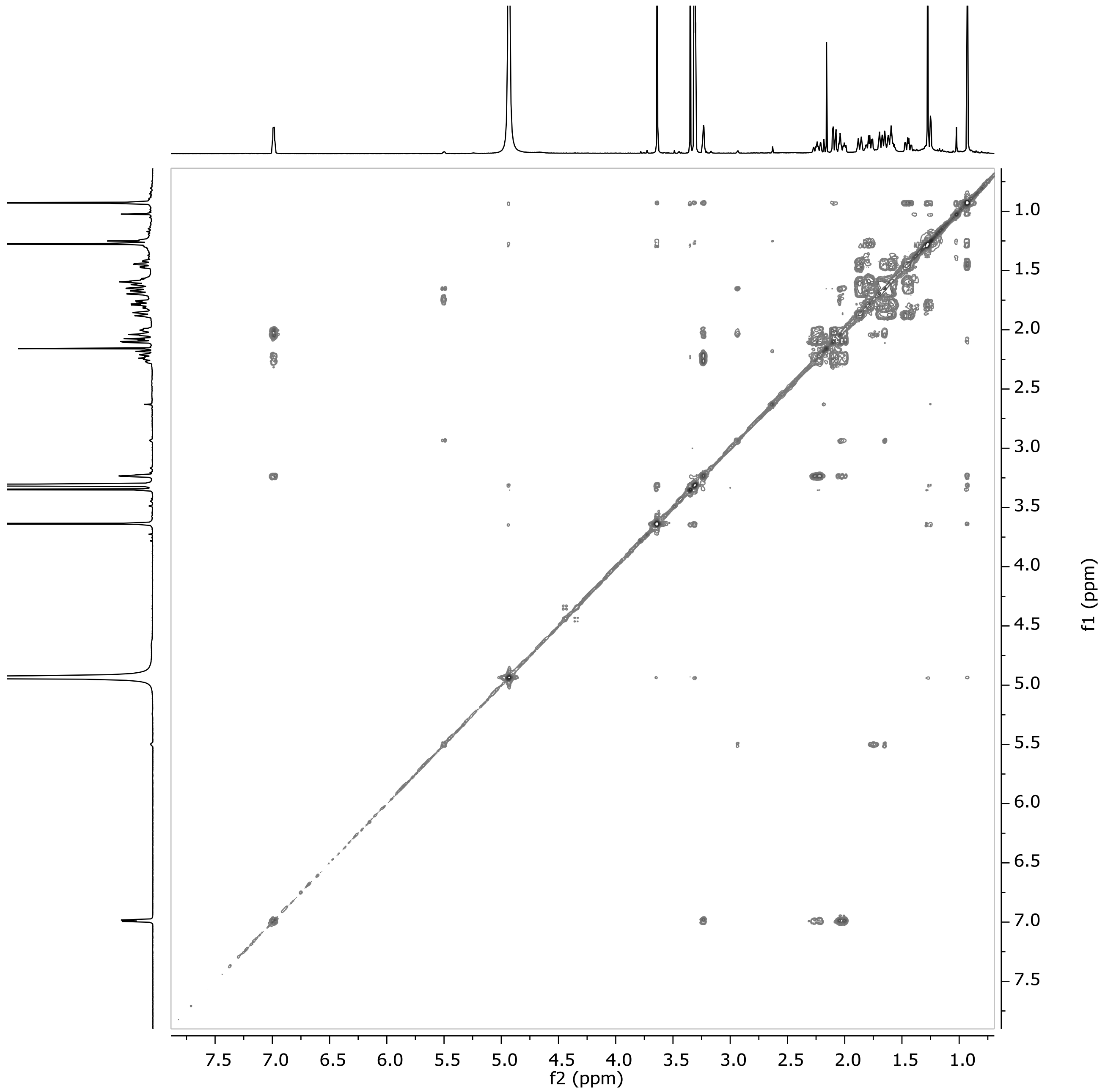


Figure S59. $^{1}H$-$^{1}H$ COSY spectrum of **7** in methanol-$d_4$ at 500 MHz.

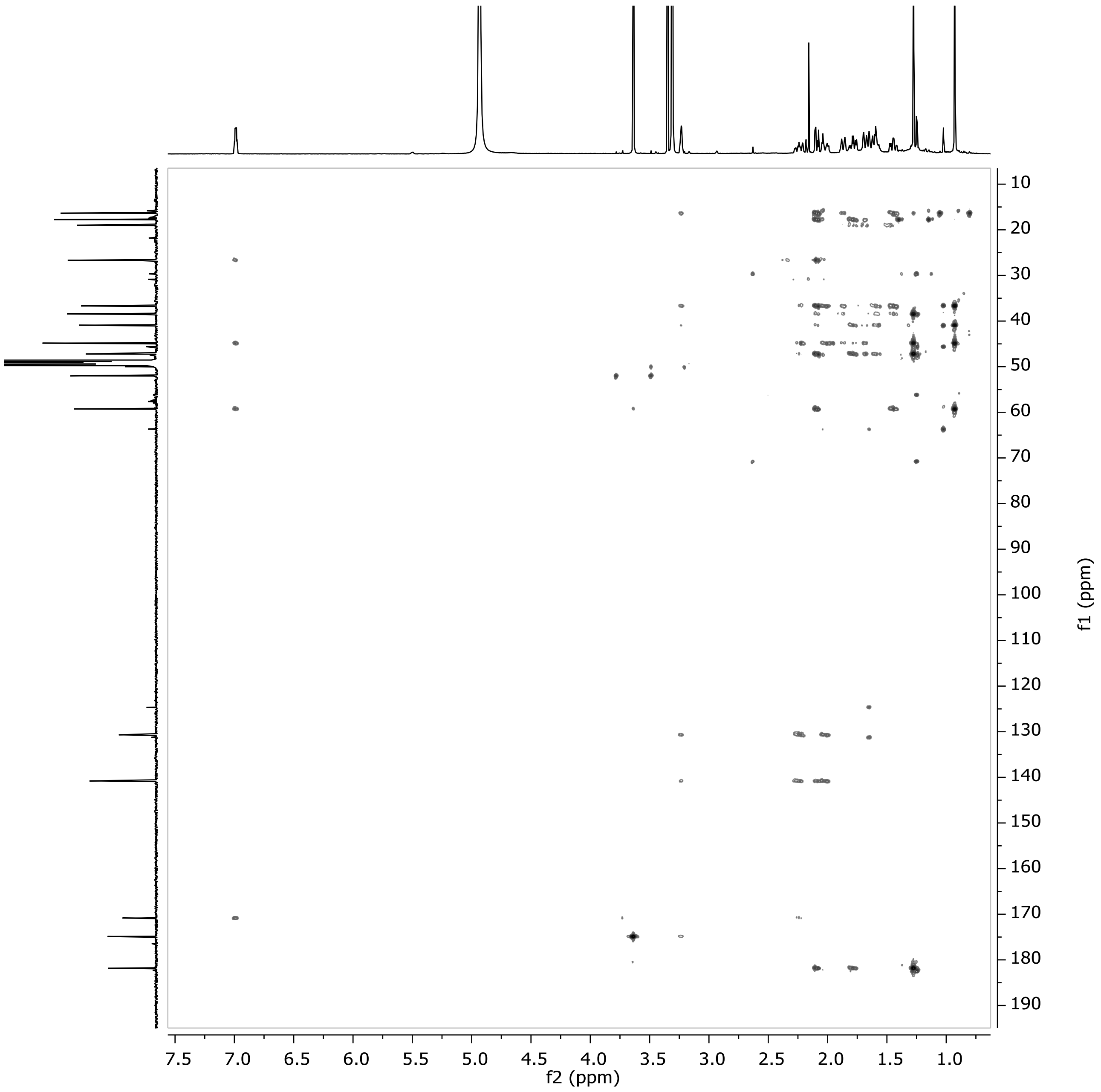


Figure S60. HMBC spectrum of **7** in methanol-$d_4$ at 500 MHz.

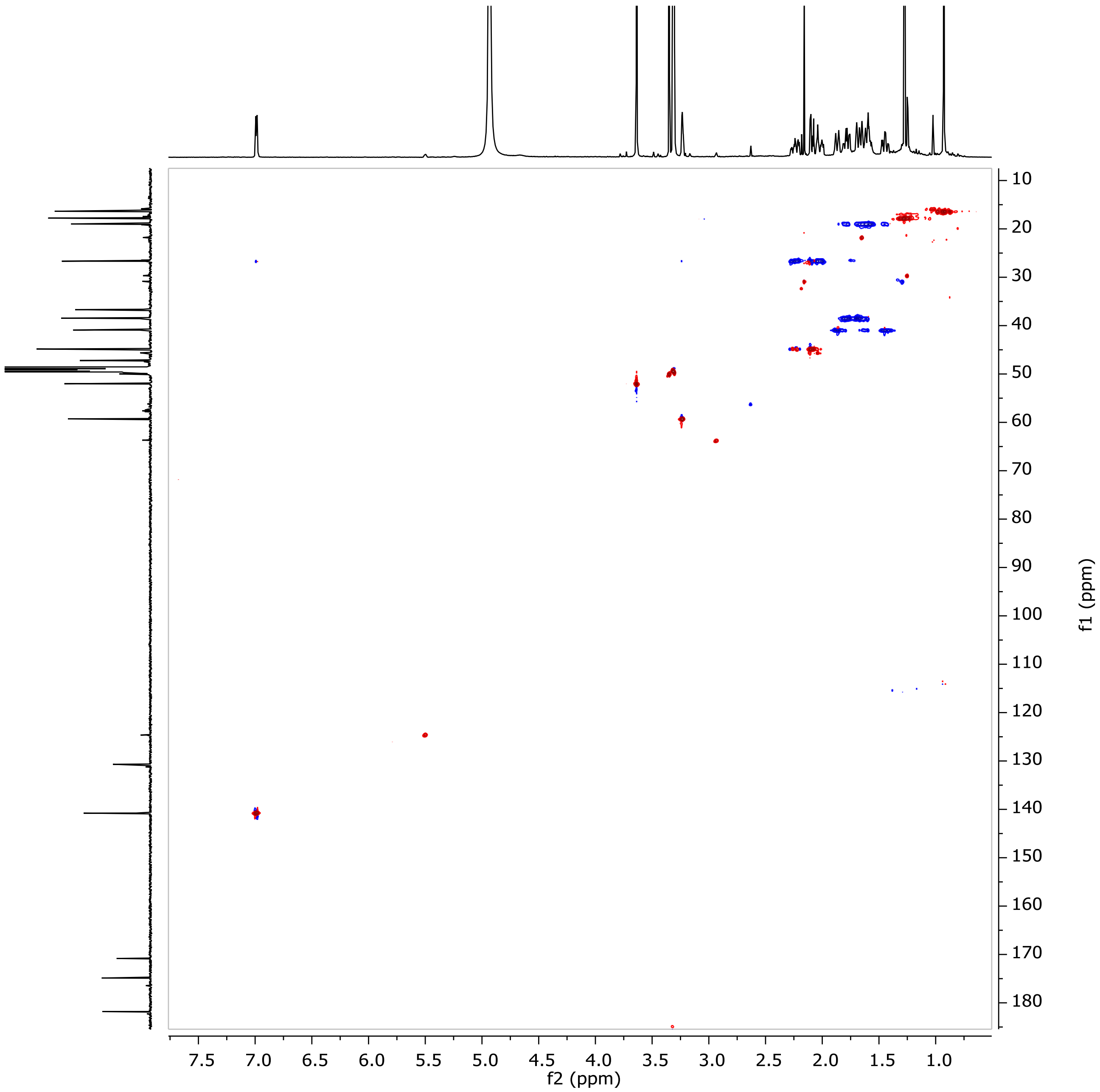


Figure S61. HSQC spectrum of **7** in methanol-$d_4$ at 500 MHz.

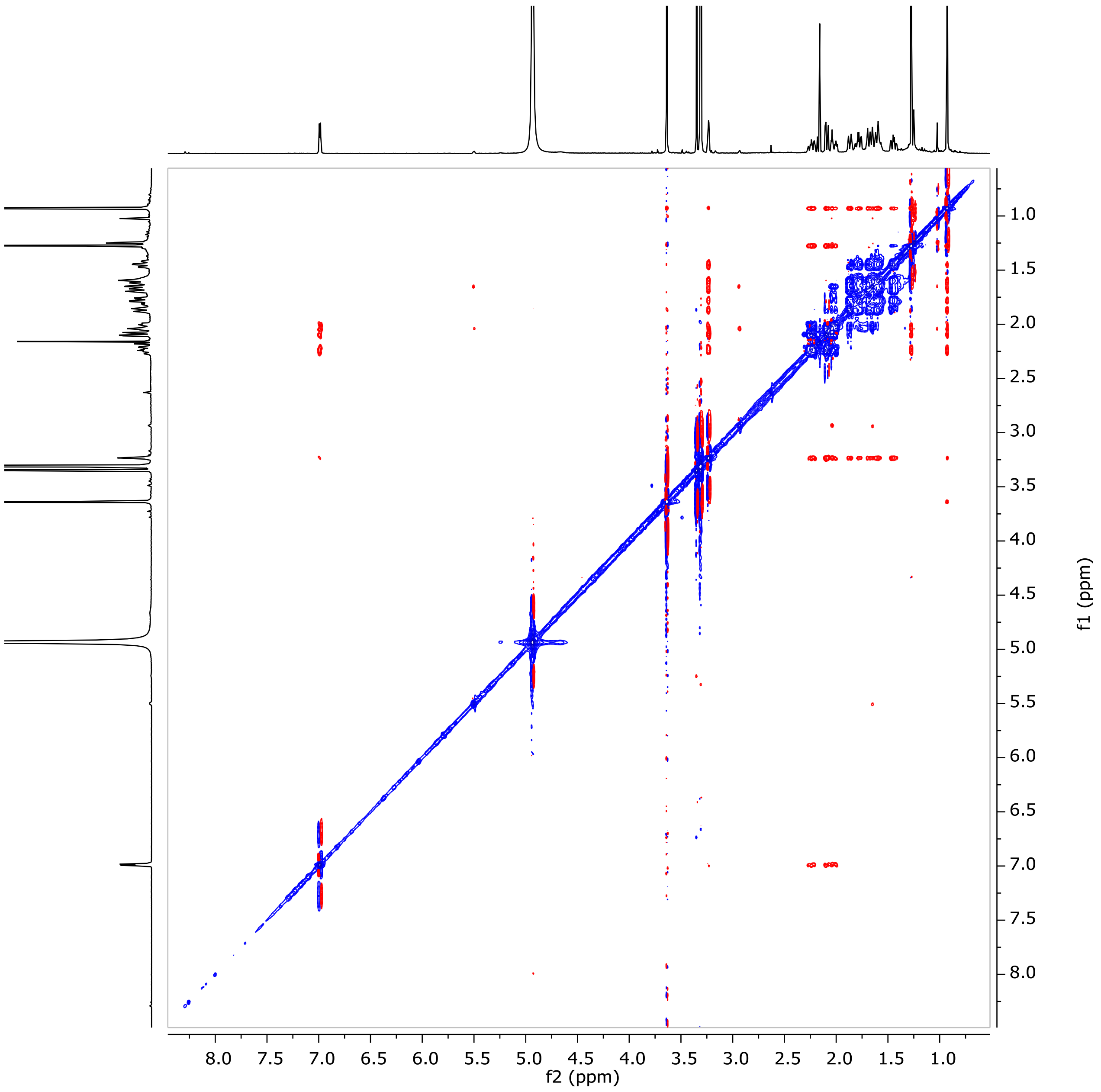


Figure S62. ROESY spectrum of **7** in methanol-$d_4$ at 500 MHz.

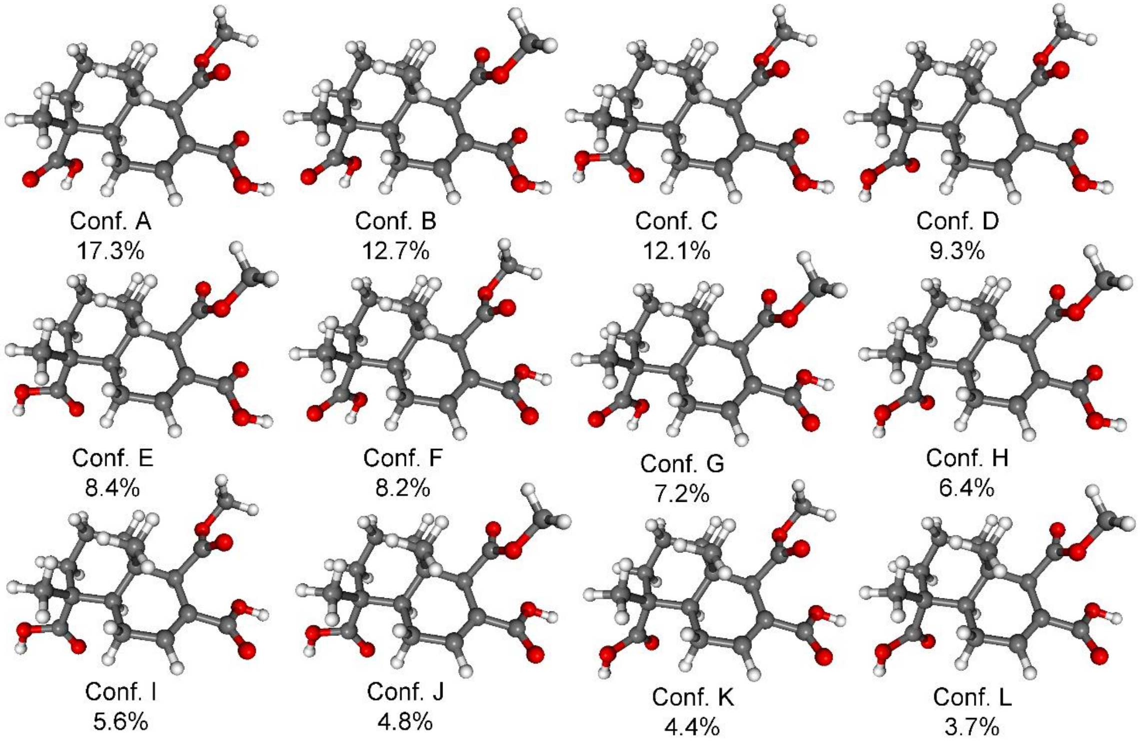


Figure S63. Low-energy ωB97X/TZVP PCM/MeOH conformers of (4*R*,5*R*,9*R*,10*S*)-**7**.

# Generic Display Report

**Analysis Info**

| | | | |
|---|---|---|---|
| | | Acquisition Date | 02.03.2022 14:50:22 |
| Analysis Name | \\Neon\MWISCOM\PEOPLE\sel22_Sherif Elsayed\Abundisporus\Amazon\A. violaceus AmaZon\Abund B1F10F1_BA8_01_10294.d | | |
| Method | [illegible]_10294.m | Operator | lab |
| Sample Name | Abund B1F10F1 | Instrument | amaZon speed |
| Comment | | | |

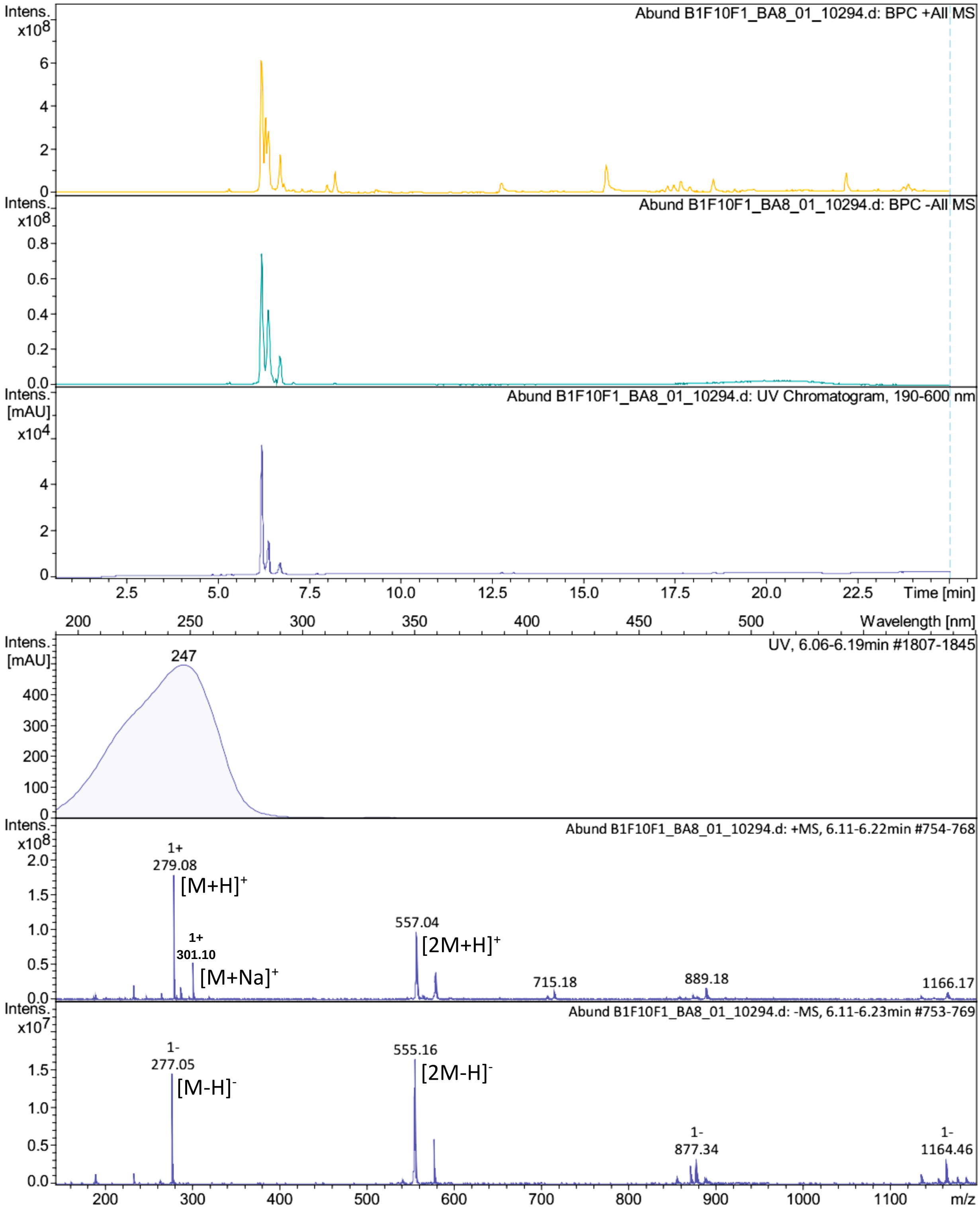


Bruker Compass DataAnalysis 4.4 printed: 11.04.2023 18:18:50 by: mk-mwisnmr Page 1 of 1

Figure S64. LRESIMS of **8**.

# Generic Display Report

**Analysis Info**

| | | | |
|---|---|---|---|
| Analysis Name | \\Neon\MWISCOM\PEOPLE\sel22_Sherif Elsayed\Abundisporus\Maxis\A. violaceus MaXis\Abundisporus | Acquisition Date | 12.05.2022 09:26:08 |
| Method | R2_B1F10F1F5_P1-A-1_01_10152.d / pos_[illegible]_screening_ms200_2500_line.m | Operator | ate06 |
| Sample Name | Abundisporus R2_B1F10F1F5 | Instrument | maXis |
| Comment | Screening01 Waters Acquity UPLC BEH C_18 1,7um 2.1x50mm | | |

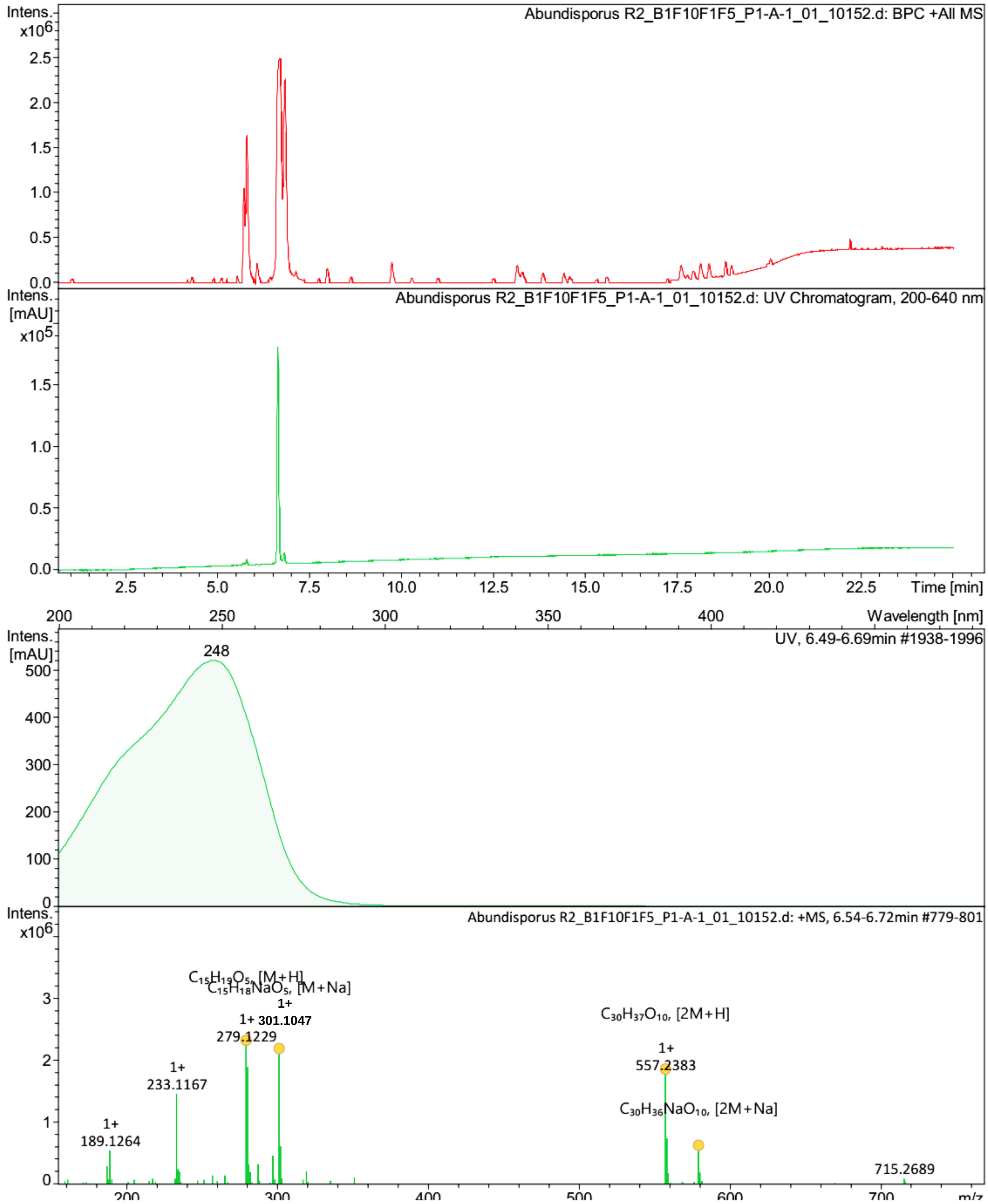


Bruker Compass DataAnalysis 4.4 printed: 11.04.2023 18:55:12 by: mk-mwisnmr Page 1 of 1

Figure S65. HRESIMS of **8**.

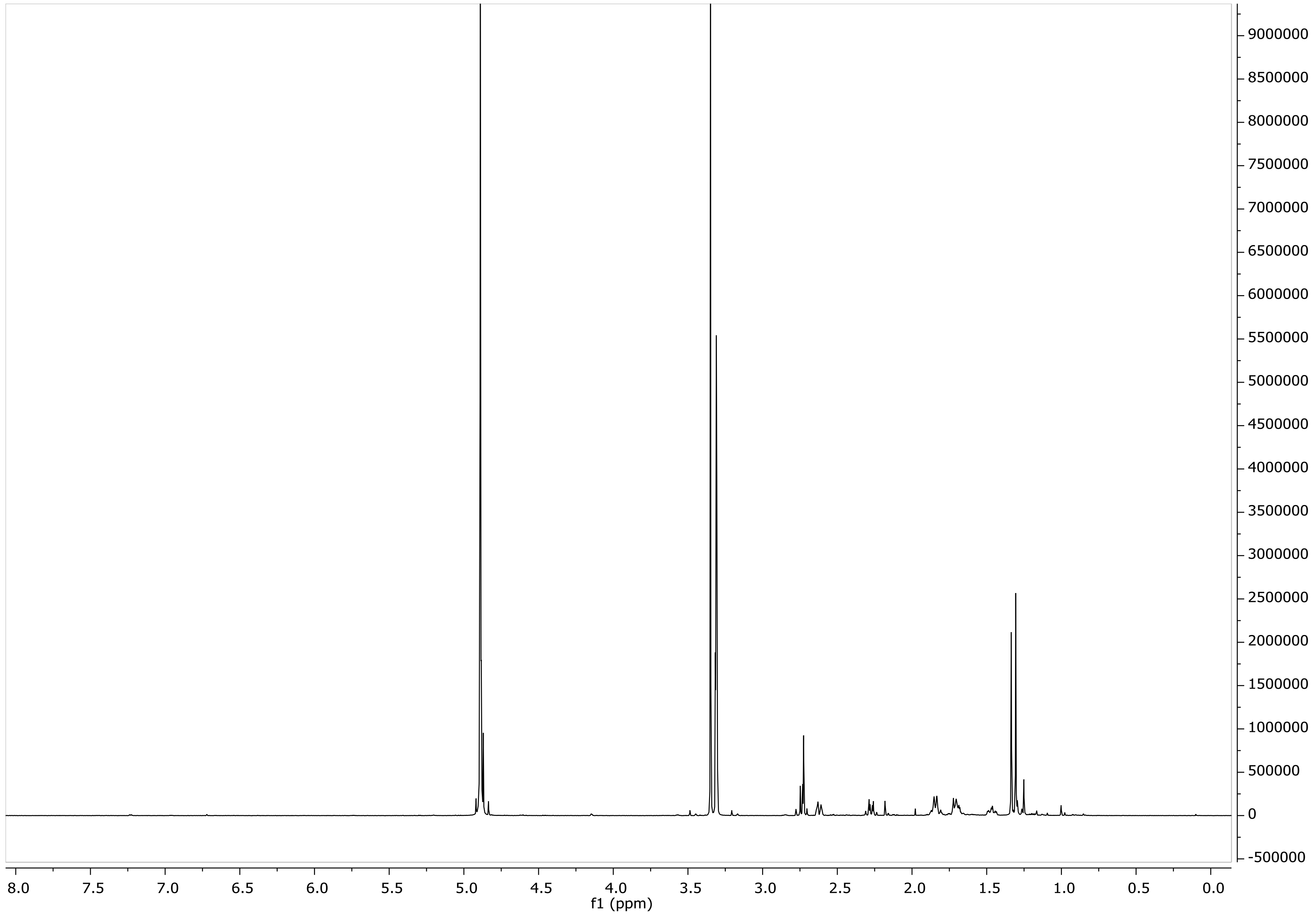


Figure S66. $^{1}$H NMR spectrum of **8** in methanol-$d_4$ at 500 MHz.

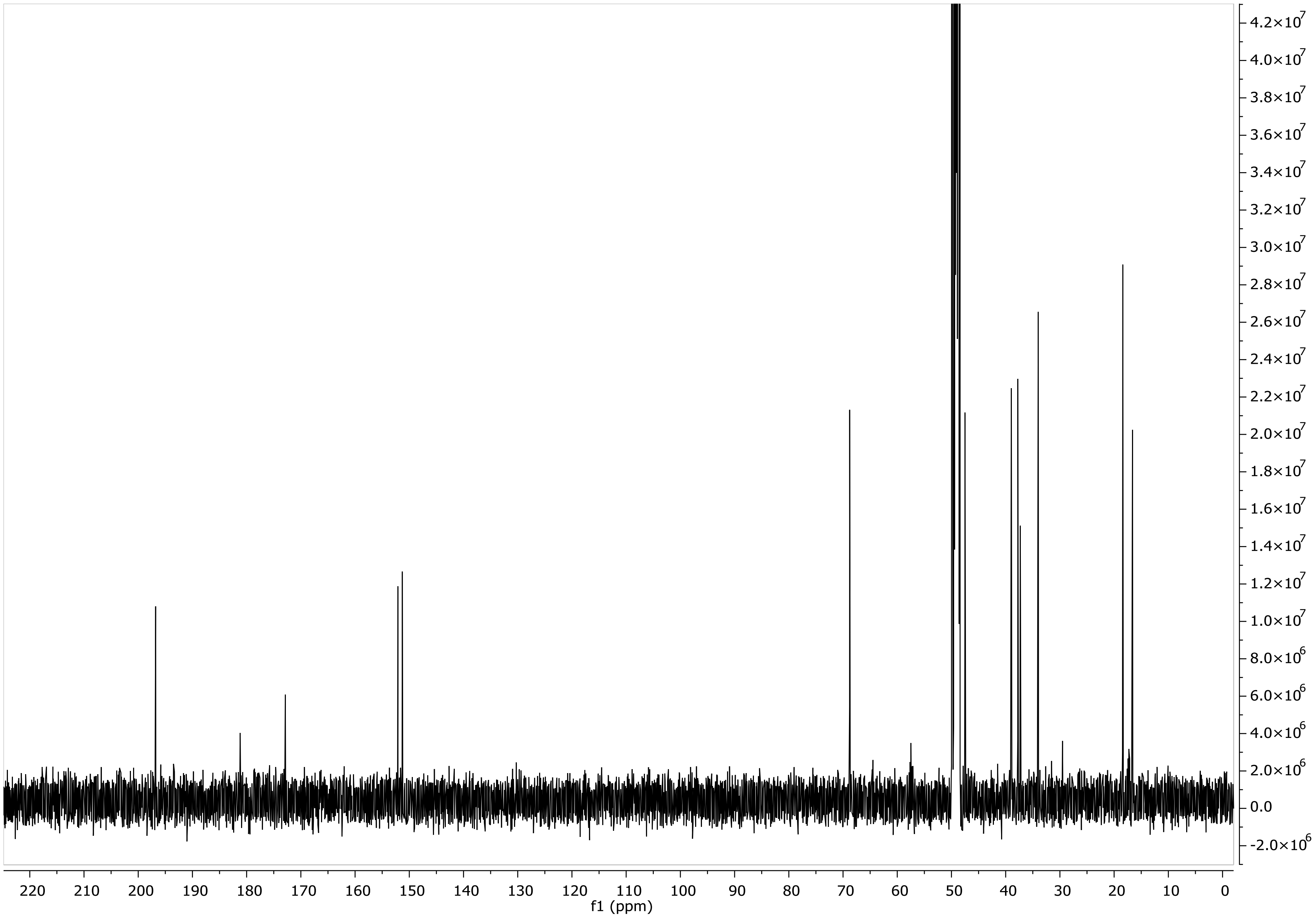


Figure S67. $^{13}$C NMR spectrum of **8** in methanol-$d_4$ at 125 MHz.

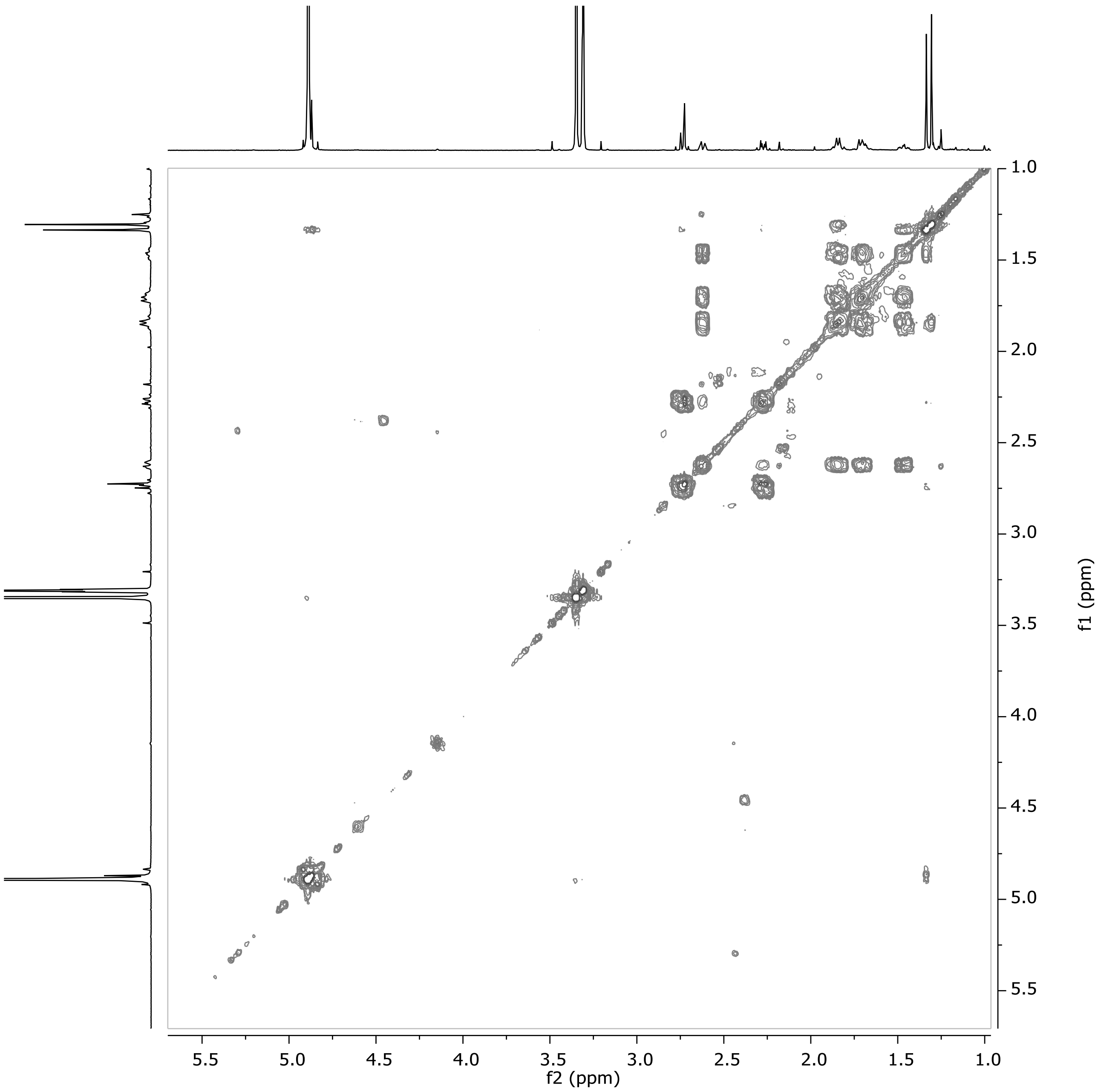


Figure S68. $^{1}H$-$^{1}H$ COSY spectrum of **8** in methanol-$d_4$ at 500 MHz.

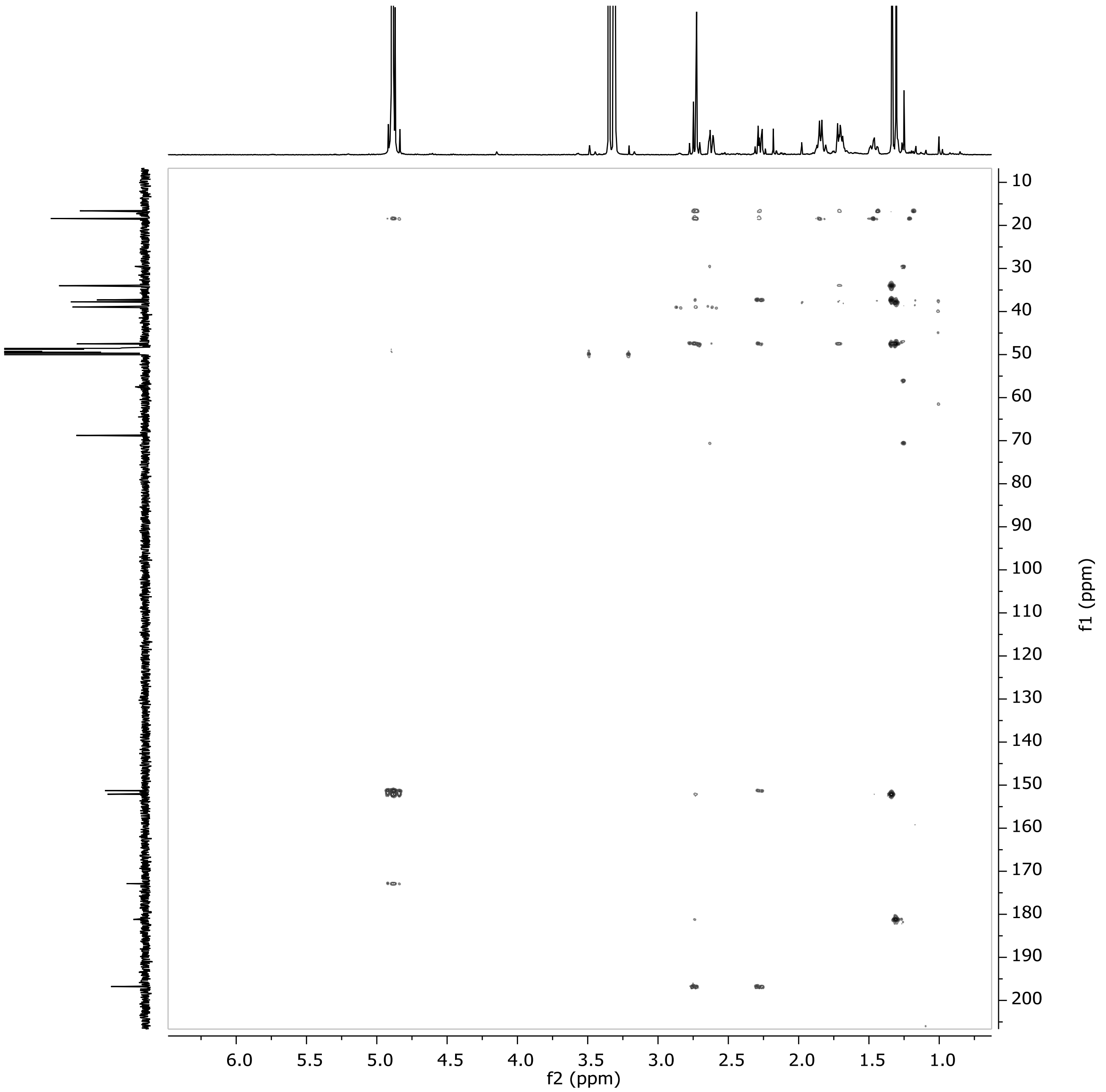


Figure S69. HMBC spectrum of **8** in methanol-$d_4$ at 500 MHz.

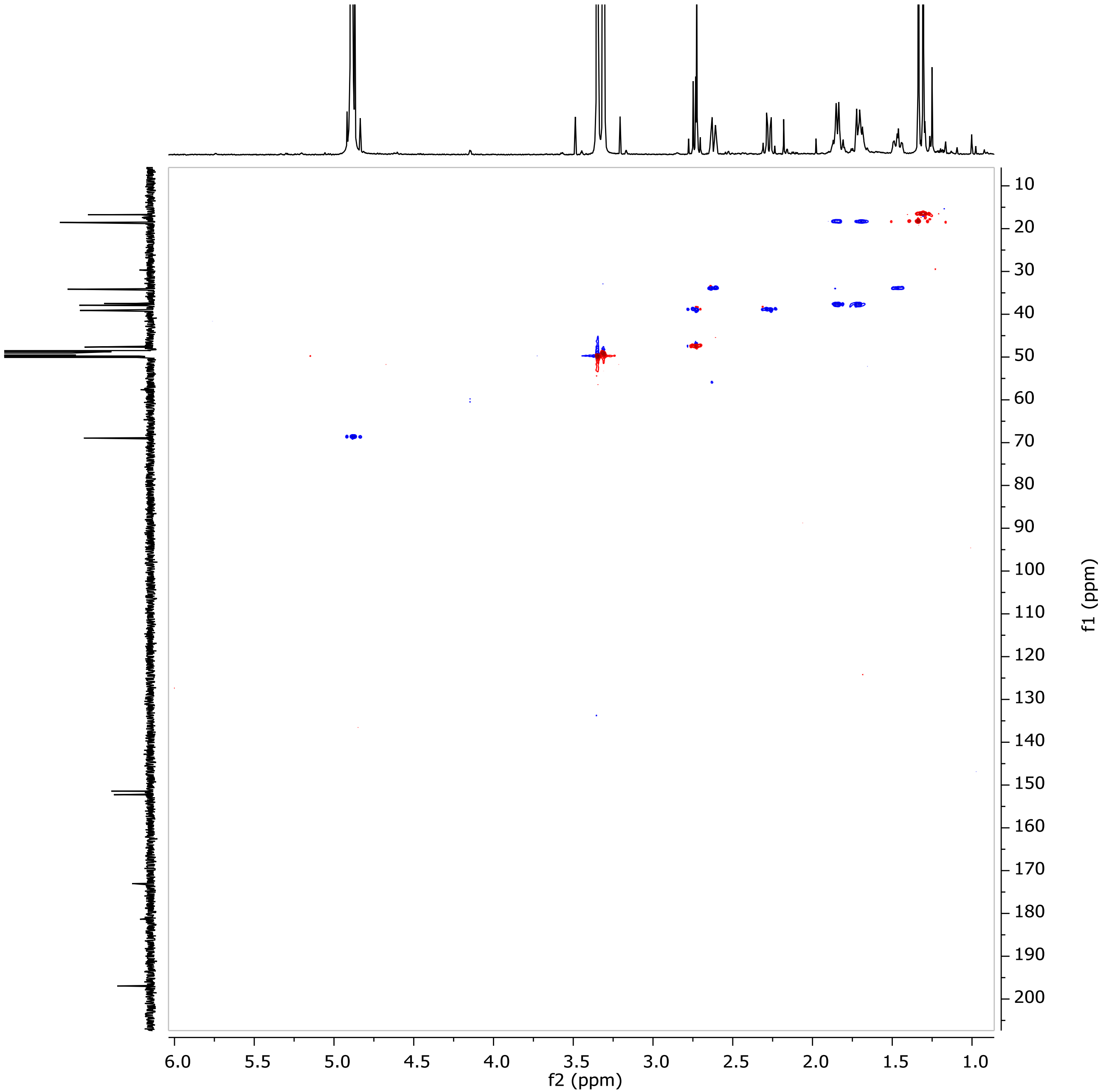


Figure S70. HSQC spectrum of **8** in methanol-$d_4$ at 500 MHz.

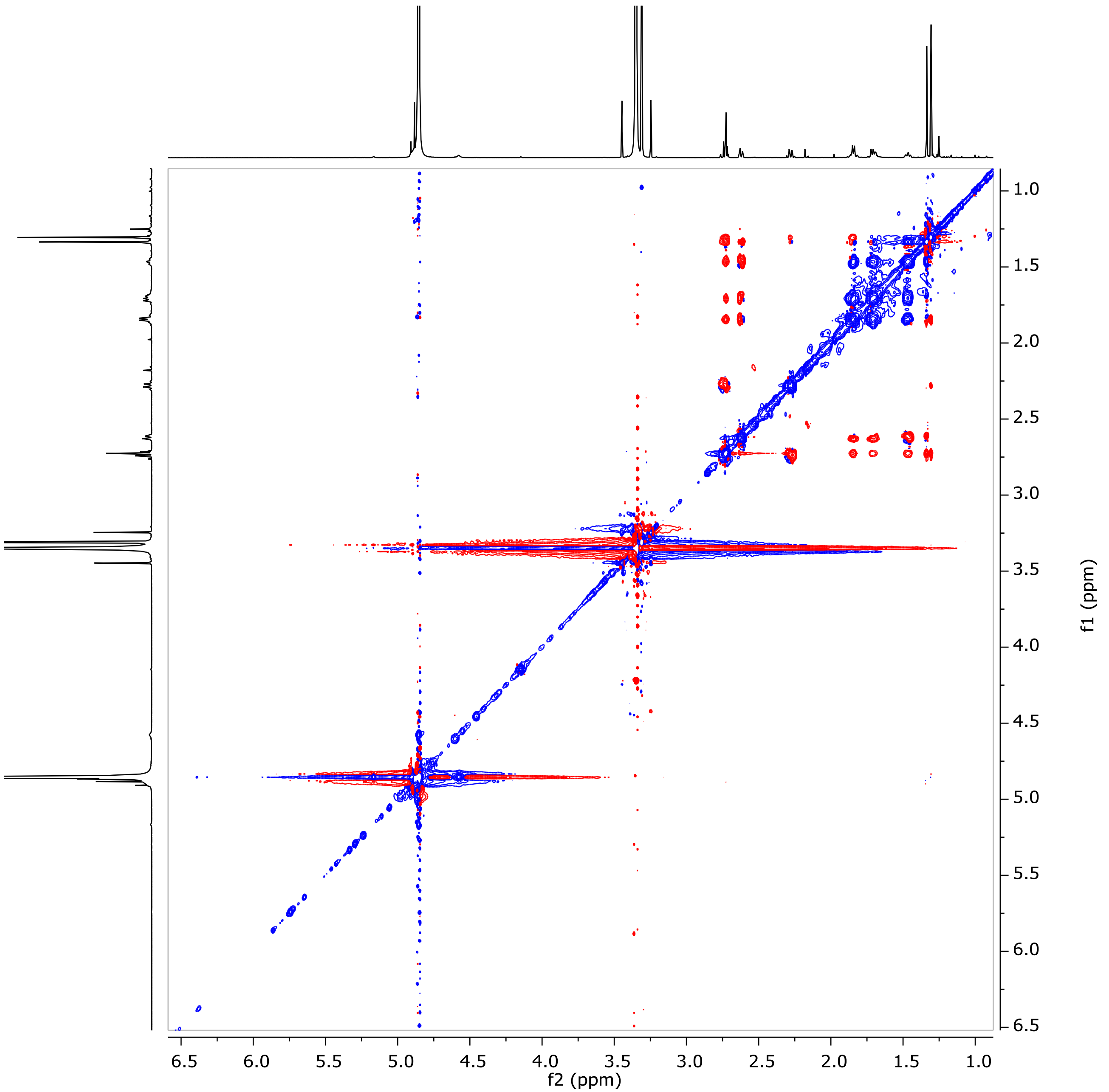


Figure S71. ROESY spectrum of **8** in methanol-$d_4$ at 700 MHz.

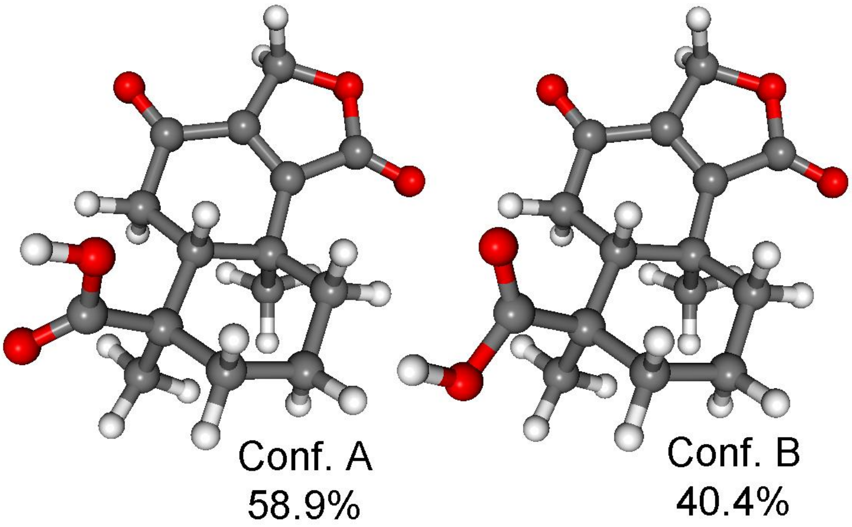


Figure S72. Low-energy ωB97X/TZVP PCM/MeOH conformers of (4*R*,5*R*,10*S*)-**8**.

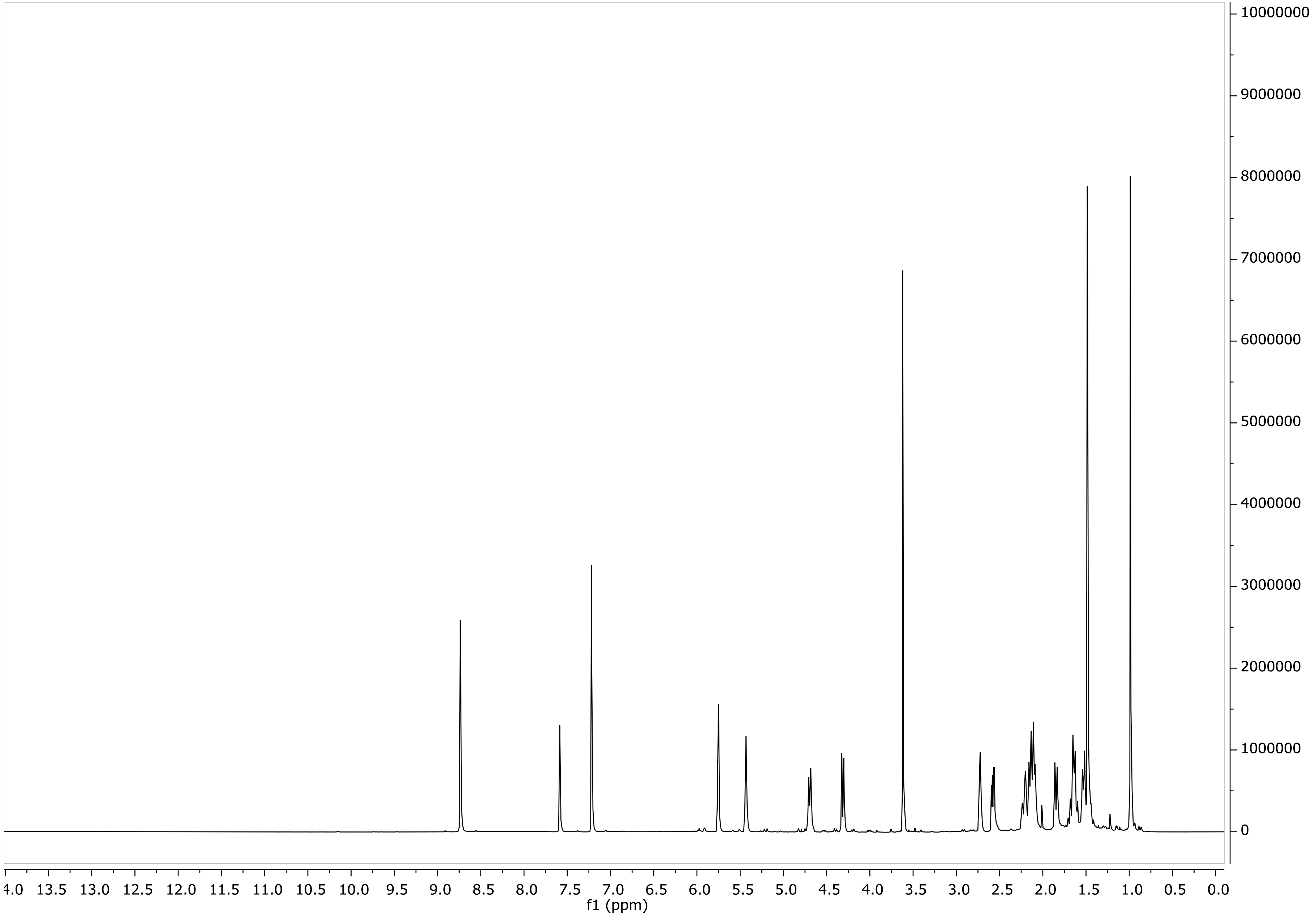


Figure S73. $^{1}$H NMR spectrum of **1** in pyridine-$d_5$ at 500 MHz.

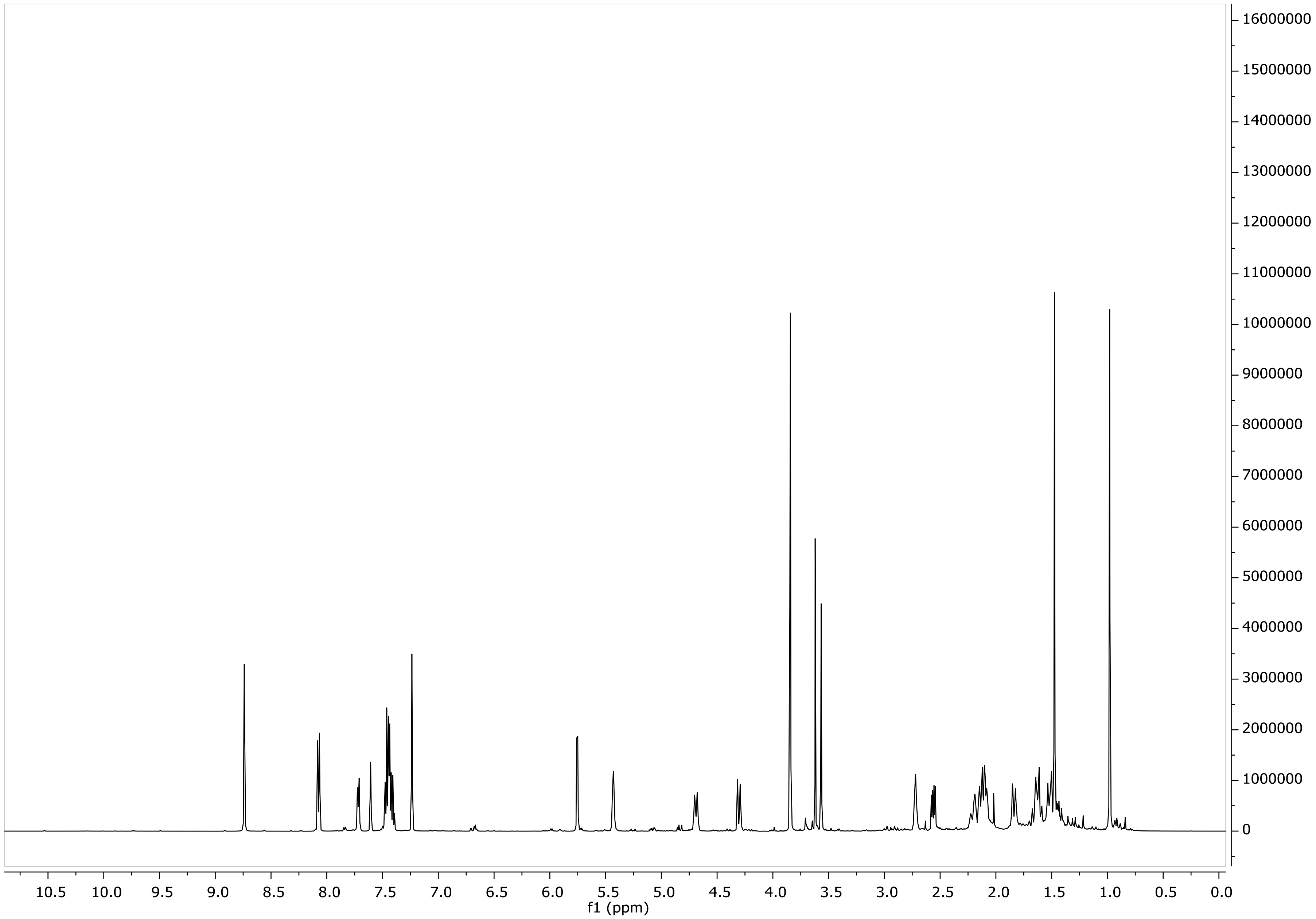


Figure S74. $^{1}$H NMR spectrum of 11-*O*-(*S*)-MTPA ester of **1** in pyridine-$d_5$ at 500 MHz.

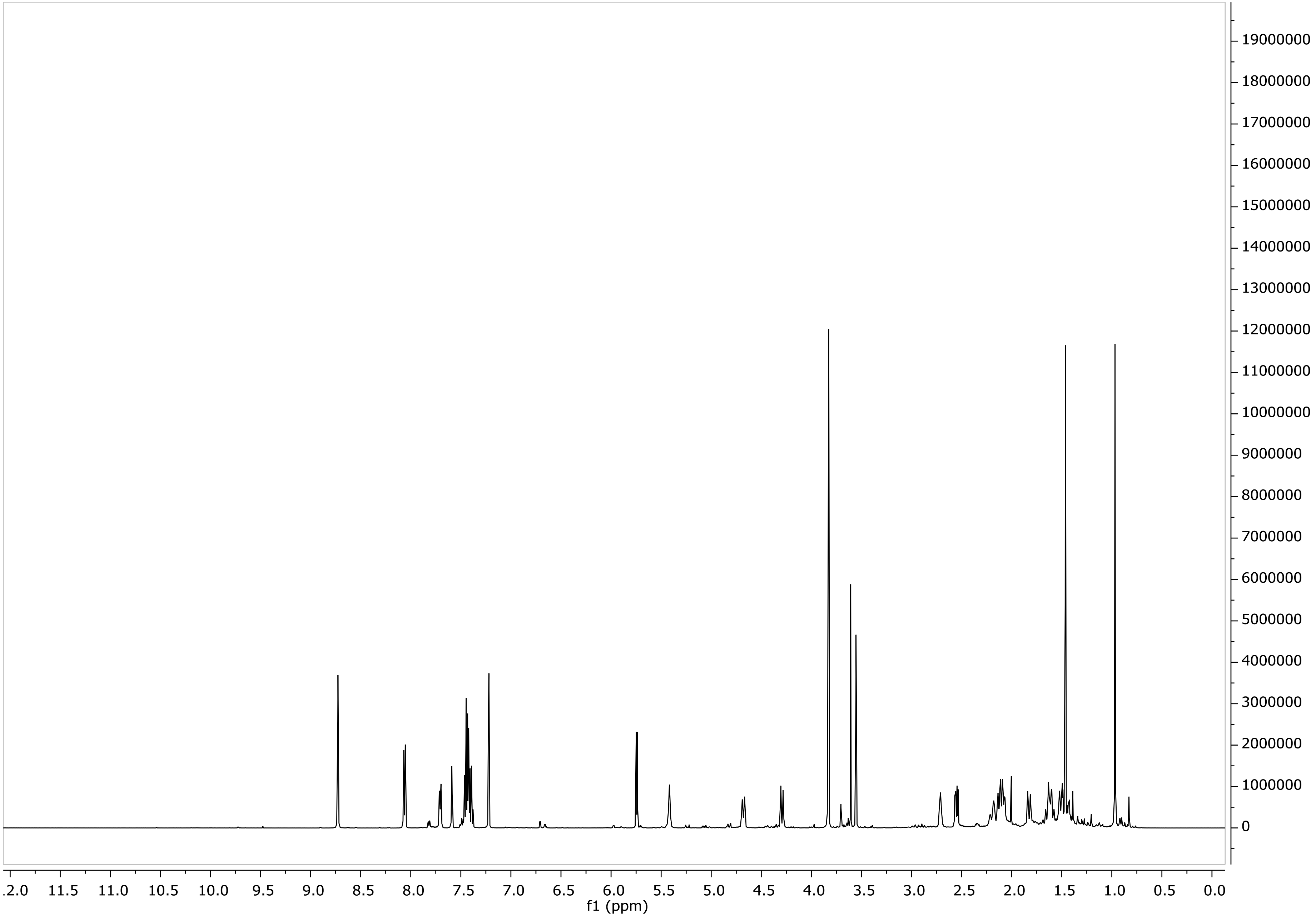


Figure S75. $^{1}$H NMR spectrum of 11-*O*-(*R*)-MTPA ester of **1** in pyridine-$d_5$ at 500 MHz.

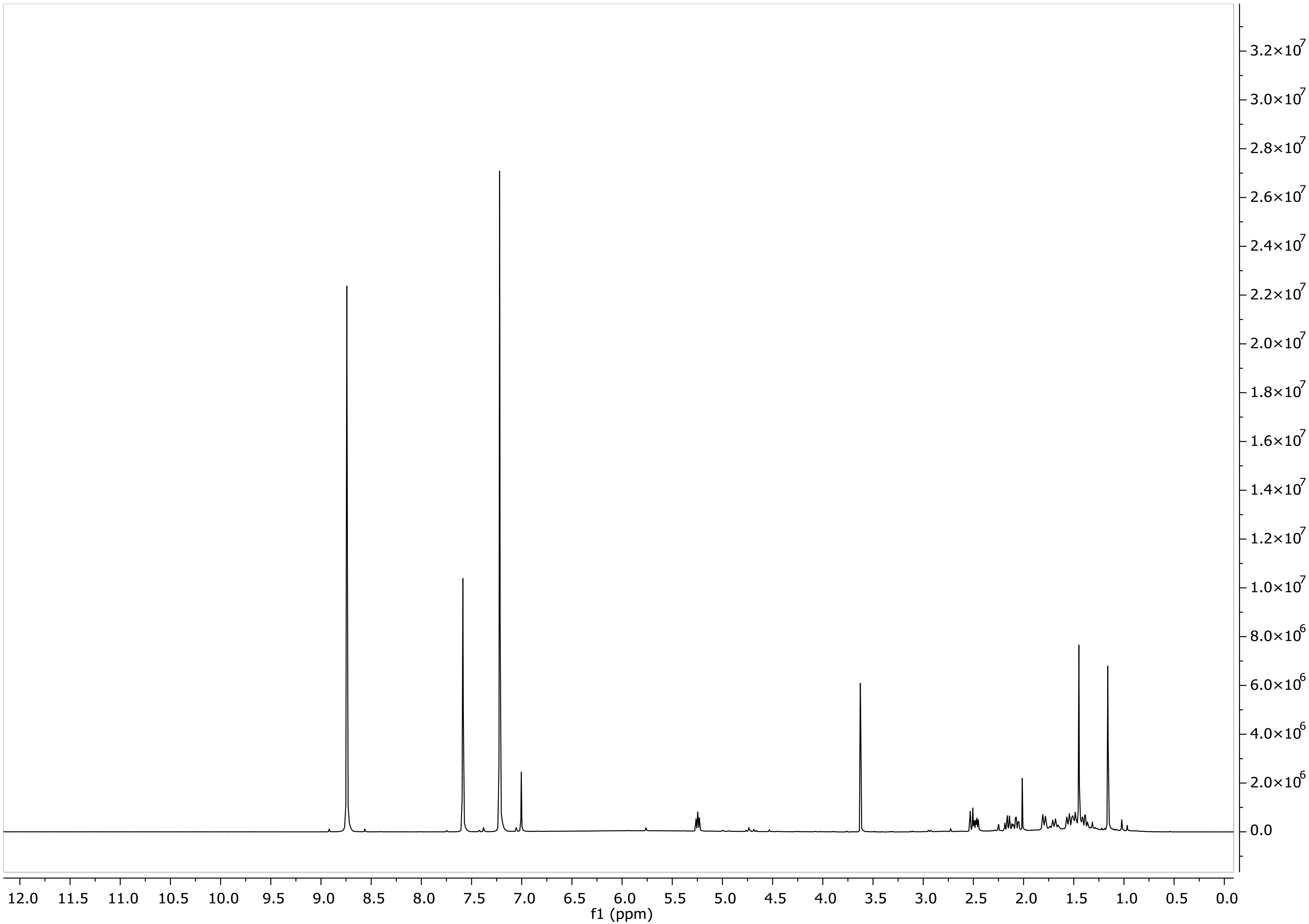


Figure S76. $^{1}$H NMR spectrum of **2** in pyridine-$d_5$ at 500 MHz.

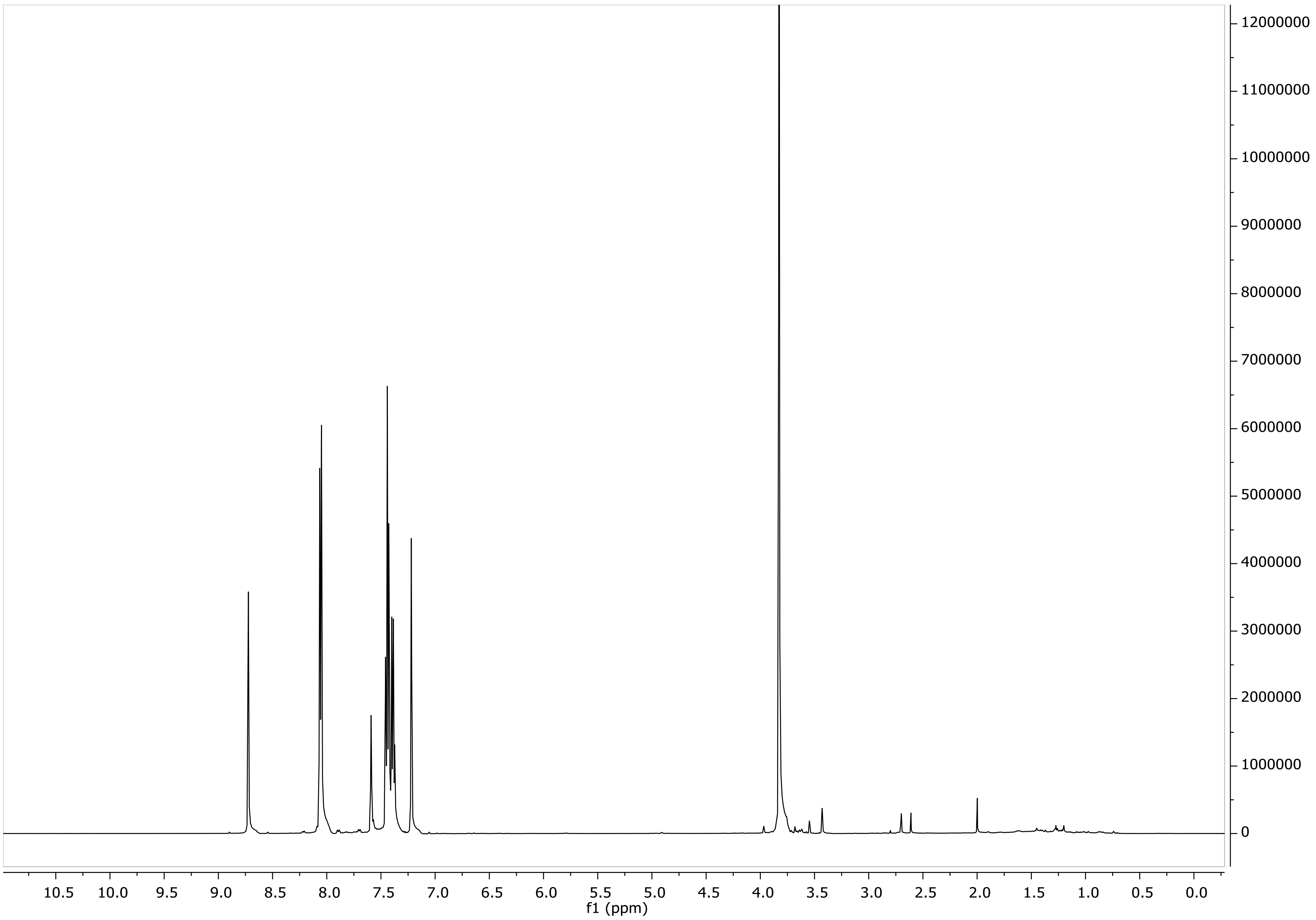


Figure S77. $^1$H NMR spectrum of 11-*O*-(*S*)-MTPA ester of **2** in pyridine-$d_5$ at 500 MHz.

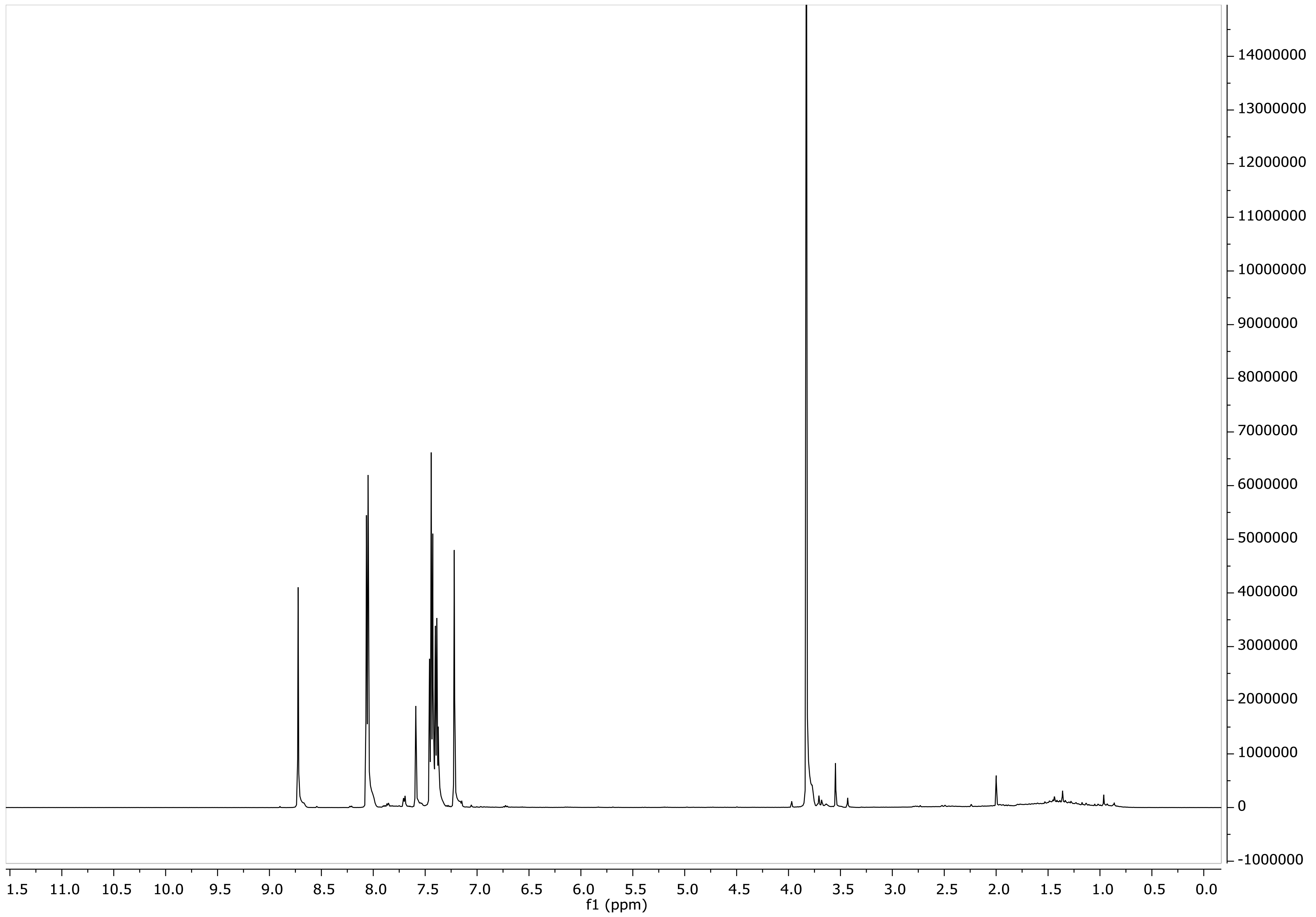


Figure S78. $^{1}$H NMR spectrum of 11-*O*-(*R*)-MTPA ester of **2** in pyridine-$d_5$ at 500 MHz.

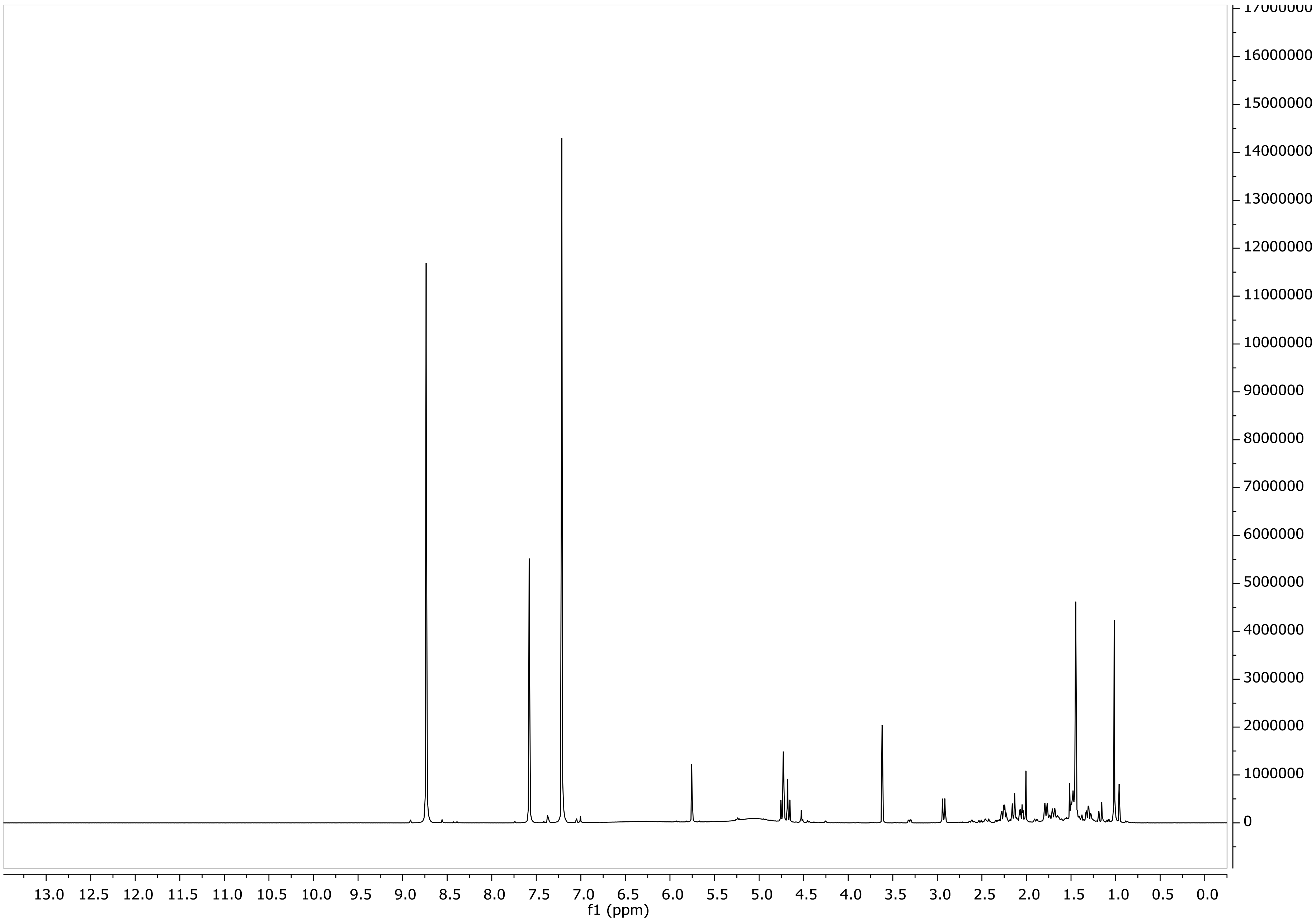


Figure S79. $^{1}$H NMR spectrum of **3** in pyridine-$d_5$ at 500 MHz.

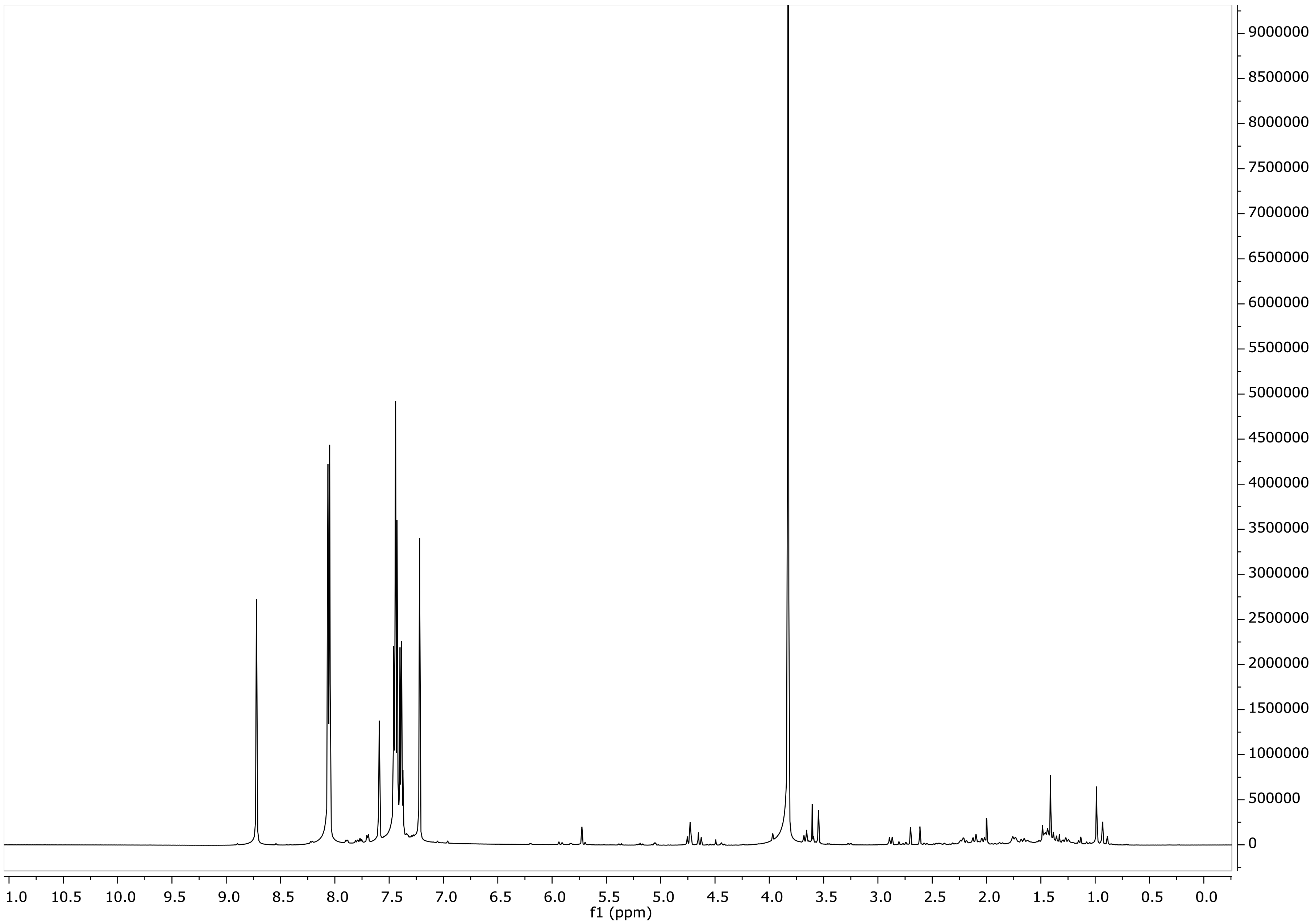


Figure S80. $^{1}$H NMR spectrum of 11-*O*-(*S*)-MTPA ester of **3** in pyridine-$d_5$ at 500 MHz.

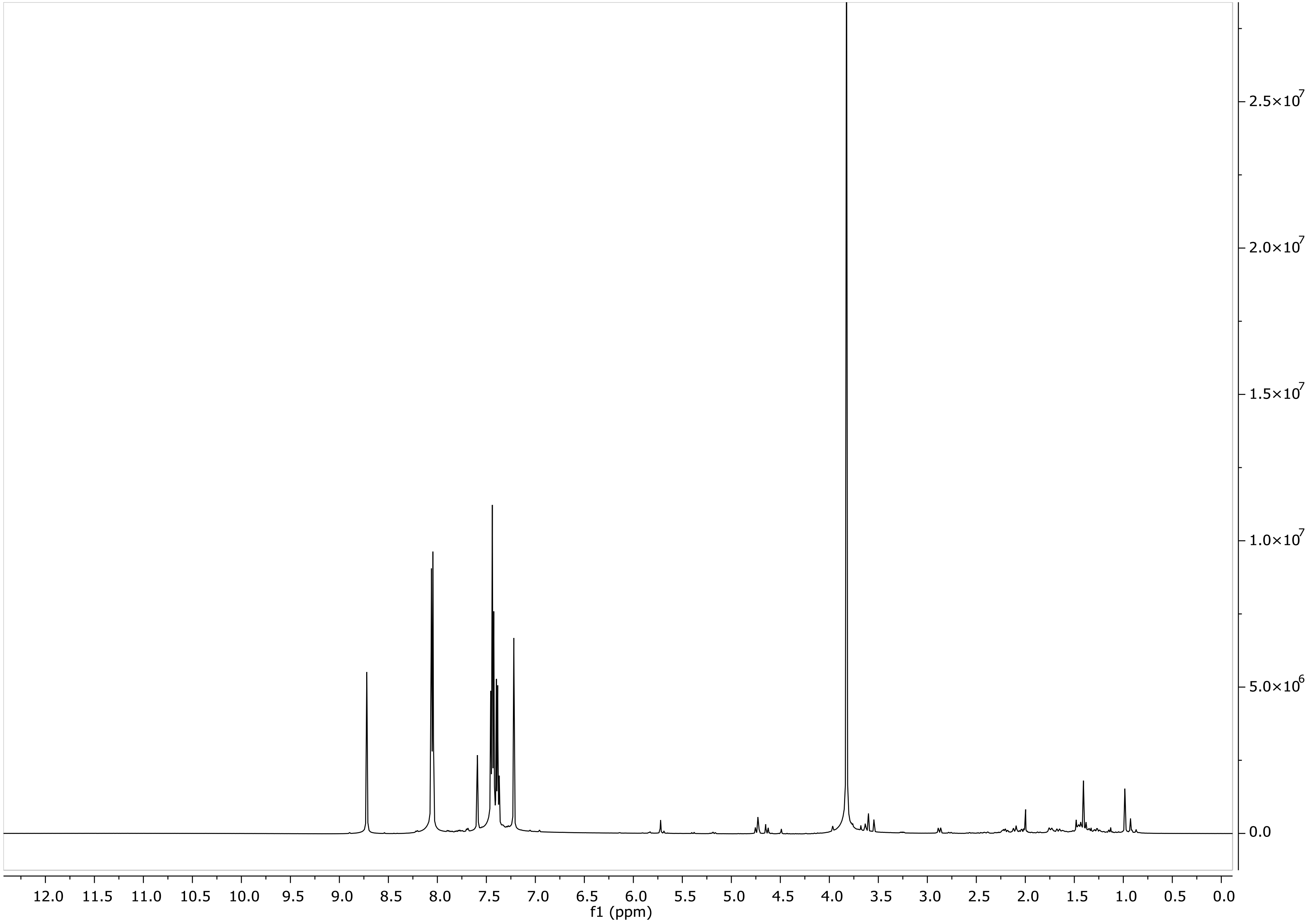


Figure S81. $^1$H NMR spectrum of 11-*O*-(*R*)-MTPA ester of **3** in pyridine-$d_5$ at 500 MHz.

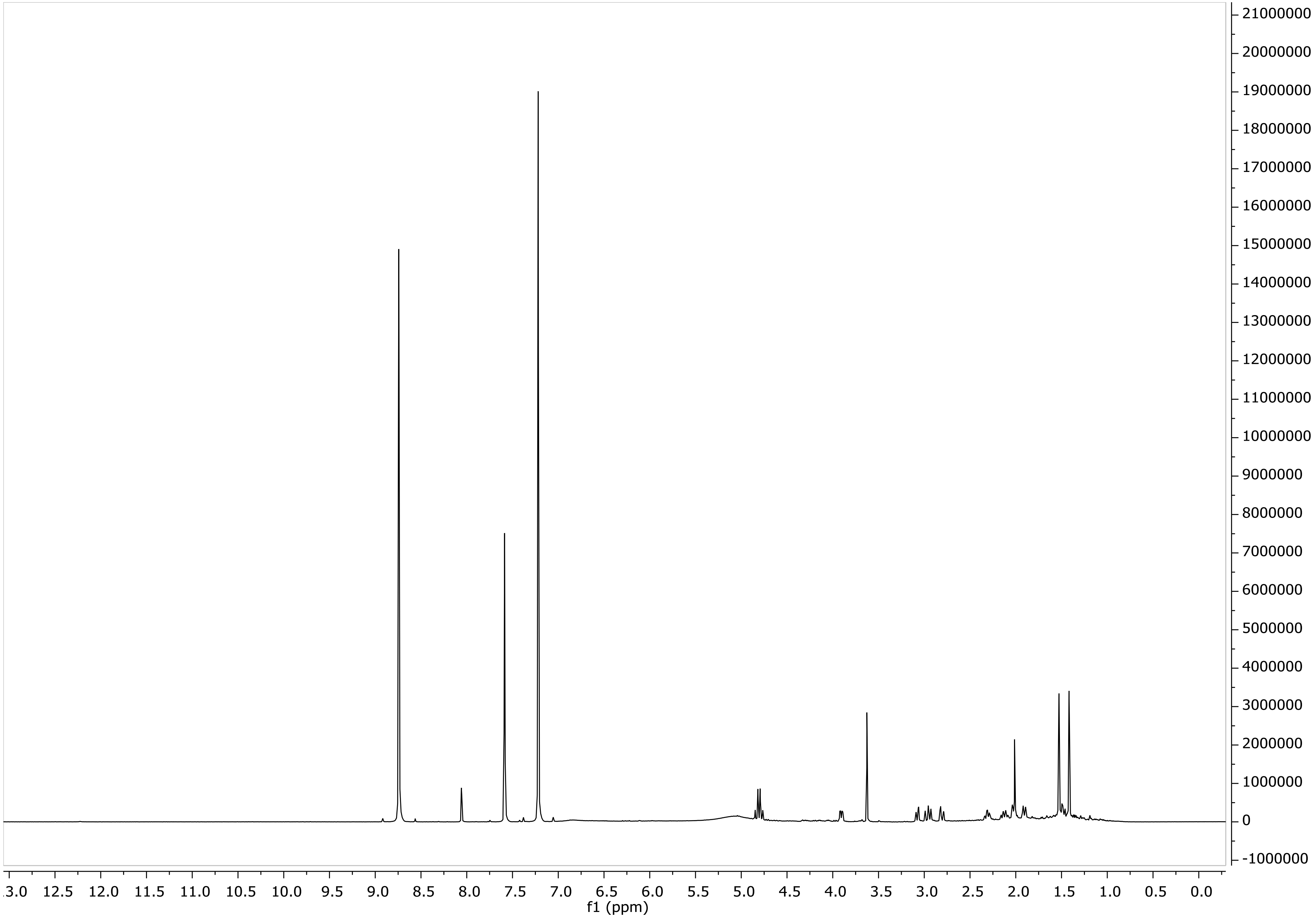


Figure S82. $^{1}$H NMR spectrum of **4** in pyridine-$d_5$ at 500 MHz.

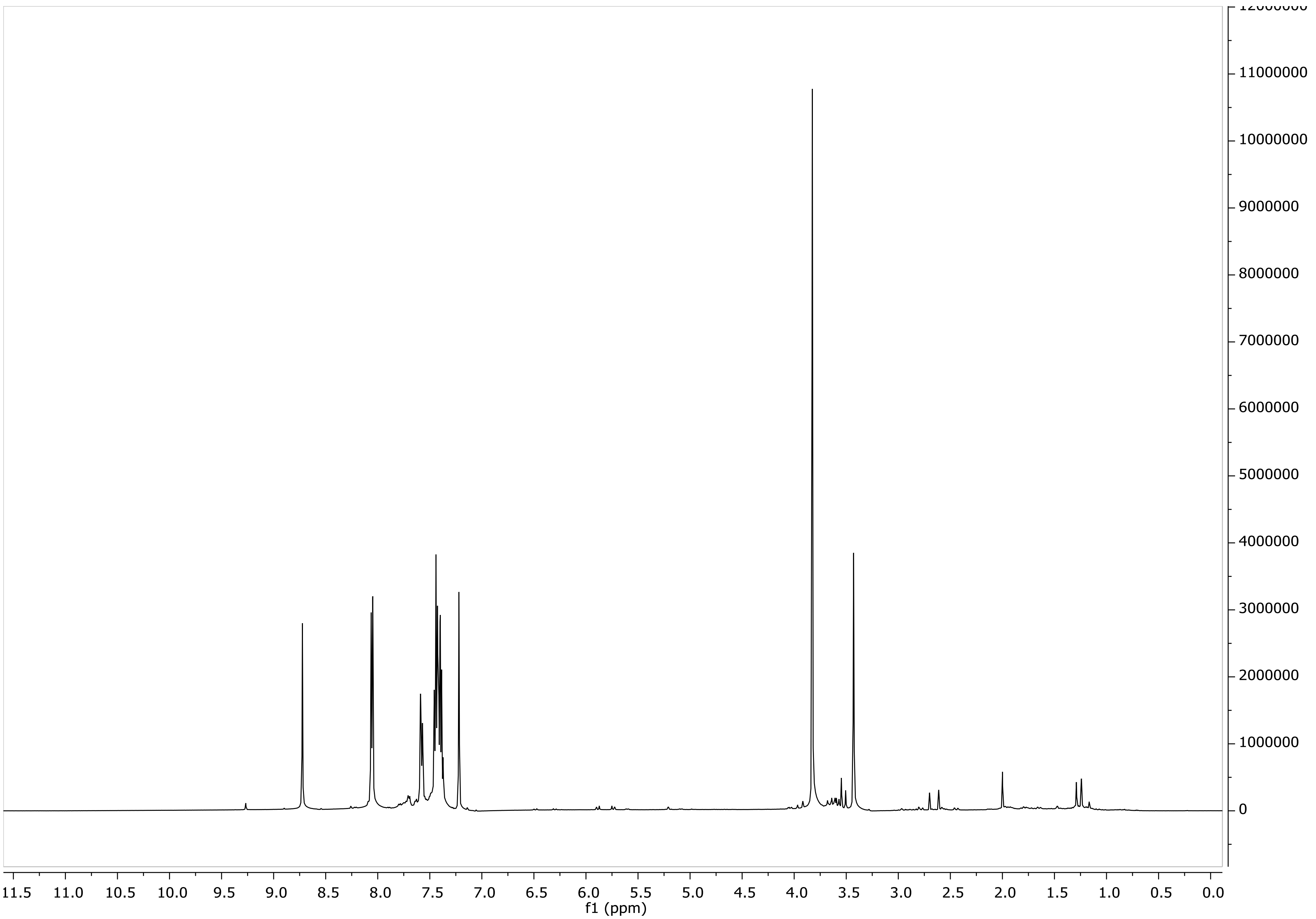


Figure S83. $^{1}$H NMR spectrum of 11-*O*-(*S*)-MTPA ester of **4** in pyridine-$d_5$ at 500 MHz.

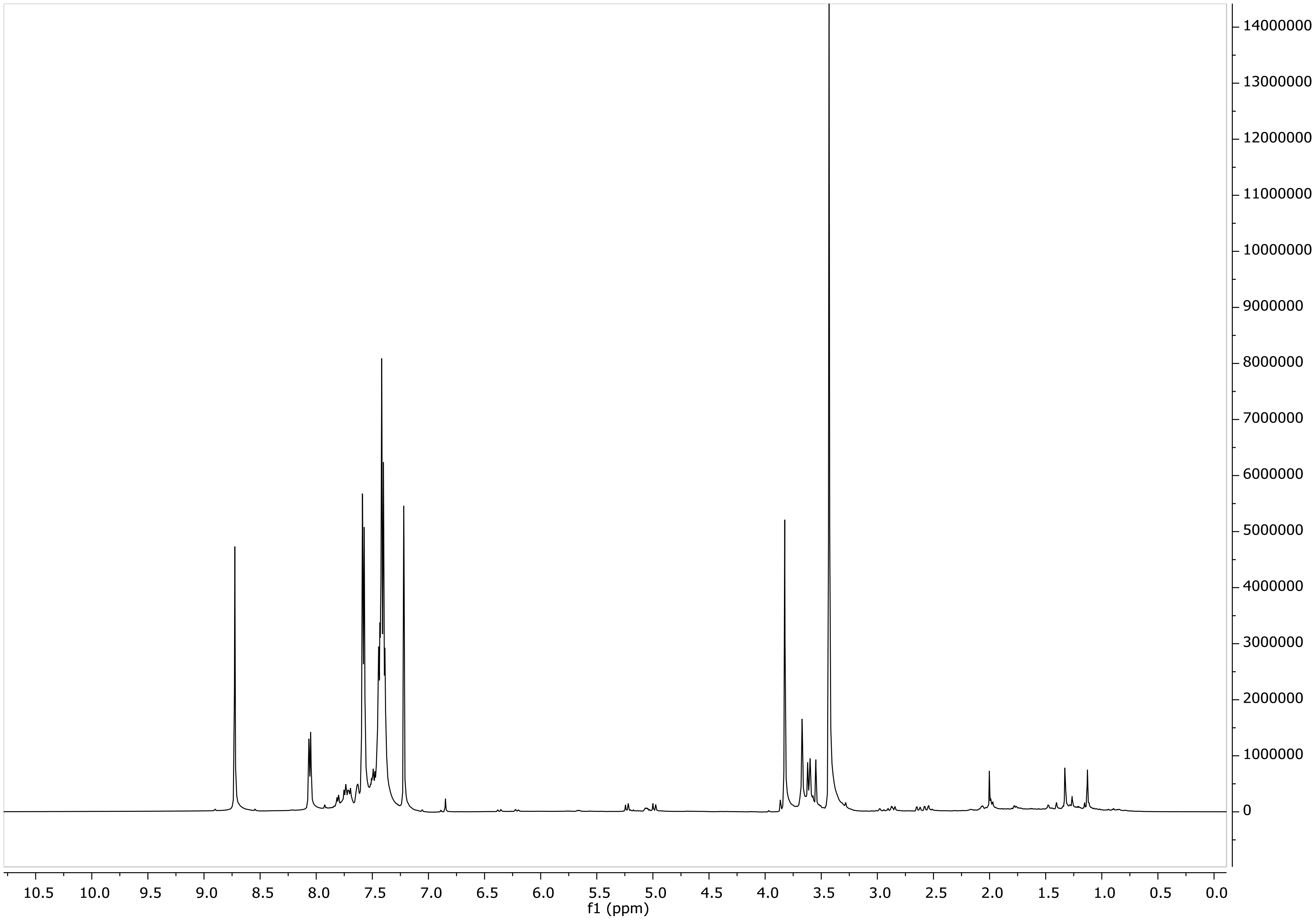


Figure S84. $^{1}$H NMR spectrum of 11-*O*-(*R*)-MTPA ester of **4** in pyridine-$d_5$ at 500 MHz.
